# Integrating metabolomics and high-throughput phenotyping to elucidate metabolic and phenotypic responses to early-season drought stress in Nordic spring wheat

**DOI:** 10.1186/s12870-025-06914-y

**Published:** 2025-07-30

**Authors:** Ronja Wonneberger, John Charles D’Auria, Kerstin Neumann, Pernille Bjarup Hansen, Jon Arne Dieseth, Linda Kærgaard Nielsen, Tarja Niemelä, Firuz Odilbekov, Fluturë Novakazi, Therése Bengtsson, the CResWheat Consortium, Mehran Patpour, Mogens Støvring Hovmøller, Outi Manninen, Merja Veteläinen, Muath Alsheikh, Susanne Windju, Pernilla Vallenback, Marja Jalli, Annika Johansson, Ahmed Jahoor, Janni Hedensvang Jørgensen, Jihad Orabi, Jeppe Reitan Andersen, Morten Lillemo, Min Lin, Rasmus Lund Hjortshøj, Charlotte Damsgård Robertsen, Marwan Alamrani, Rodomiro Octavio Ortiz Rios

**Affiliations:** 1 Swedish University of Agricultural Sciences, PO Box 190, Lomma, 23422 Sweden; 2 Leibniz Institute of Plant Genetics and Crop Plant Research, Corrensstraße 3, Gatersleben, 06466 Germany; 3 Nordic Seed A/S, Kornmarken 1, Galten, 8464 Denmark; 4 Graminor AS, Hommelstadvegen 60, 2322 Ridabu, Norway; 5 Sejet Plant Breeding, Nørremarksvej 67, Horsens, 8700 Denmark; 6 Boreal Plant Breeding Ltd, Myllytie 10, Jokioinen, 31600 Finland; 7 Lantmännen ek. För., Udda Lundqvists väg 11, Svalöv, 268 31 Sweden; 8 Present Address: University of Rostock, Satower Str. 48, Rostock, 18059 Germany

**Keywords:** Climate change, Crop resilience, Drought stress, High-throughput phenotyping, Metabolomics, Spring wheat

## Abstract

**Background:**

Understanding the metabolic responses of wheat to drought stress is essential for developing strategies to enhance its resilience under water-deficit conditions. In this study, we investigated the metabolic and phenotypic responses of twelve Nordic spring wheat genotypes subjected to drought stress over 28 days in a high-throughput phenotyping facility. By integrating metabolic profiling with phenotypic assessments, we aimed to identify key metabolites and traits associated with drought tolerance.

**Results:**

We identified nearly 200 metabolites that were differentially accumulated across four time points, including early drought and recovery phases. Of these, 25% were organic acids, 16.2% sugars and derivatives, 16.2% amino acids and derivatives, and 10.4% alkaloids, while the rest were mainly lipids, nucleotides and derivatives, and phenolic acids. Furthermore, 32 metabolites showed significant correlations with 17 phenotypic traits, highlighting potential biomarkers for drought tolerance. These metabolic markers could be utilized in screening programs to accelerate the breeding of drought-resilient spring wheat. Our findings suggest that metabolomic changes during drought stress and recovery involve critical pathways linked to osmoprotection, antioxidant activity, and energy metabolism, which differentiate tolerant from non-tolerant genotypes.

**Conclusion:**

This study demonstrates the effectiveness of combining metabolomics with high-throughput phenotyping to dissect plant stress responses. By identifying key metabolic pathways and potential biomarkers for drought tolerance, our findings provide a valuable foundation for breeding climate-resilient wheat varieties. Moreover, this integrative approach enhances our understanding of plant adaptation to abiotic stress, contributing to future efforts in sustainable agriculture and food security.

**Supplementary Information:**

The online version contains supplementary material available at 10.1186/s12870-025-06914-y.

## Background

Wheat (*Triticum aestivum* L.) is among the three most important cereal crops worldwide, providing a primary source of calories and protein for 30% of the global population [1, 2]. To meet the needs of a growing population, wheat production must increase by 50% over the next 25 years, with stable yields being essential for global food security [3]. However, wheat cultivation faces numerous biotic and abiotic challenges, with drought being one of the major limiting factors, causing average yield losses of 27.5% and severely compromising product quality [4].

Many important wheat-growing regions, including the Mediterranean, the Middle East and parts of Australia, regularly experience drought, which is expected to worsen with climate change [5]. The Nordic countries, which have traditionally benefitted from a temperate climate favourable for spring wheat cultivation, are predicted to see some positive impacts of climate change on food production. These include longer growing seasons, higher temperatures, and an increase in both yield and arable land [6]. However, climate change is also anticipated to lead to more frequent occurrences of extreme weather events. The 2018 drought, coupled with extreme temperatures in the Nordic countries, severely reduced cereal yields, adversely affecting the economic situation of farmers and leading to feed shortages for livestock production [7, 8]. Although annual precipitation and heavy rainfall events are predicted to increase, the annual drought period is expected to extend by 1–2 days per year in southern Scandinavia [6]. In the boreal zone of central Fennoscandia, early season droughts, which are already a problem, are expected to become more severe [9, 10].

Drought stress causes various physiological, morphological, and biochemical changes in plants. Reduced plant-available water in the soil decreases water uptake through the roots, leading to a loss of cell turgor. To mitigate this, the plant closes its stomata, a process mediated by the hormone abscisic acid (ABA), to limit water loss through evapotranspiration [11]. Reduced cell turgor can result in stunted growth, as turgor is required for cell elongation [12]. Stomata closure results in a reduction of CO_2_ concentration in the tissue as well as an accumulation of reactive oxygen species (ROS) such as H_2_O_2_ in the mitochondria, peroxisomes and chloroplasts. This, together with a reduced specificity of the enzyme ribulose-1,5-biphosphate carboxylase/oxygenase (Rubisco) for CO_2_ leads to a reduction of photosynthetic activity and an increase in photorespiration [13, 14]. Photosynthesis decreases further through reduced photosynthetically active area, e.g. leaf and stem area. ROS must be neutralized by detoxifying enzymes before they can cause lasting damage to the cells, otherwise, tissue damage, senescence and cell death may occur [15]. Plants also produce soluble, low-molecular-weight compounds to control the cell’s osmotic potential [16]. These osmolytes or osmoprotectants regulate and stimulate water uptake to maintain turgor, reduce ROS levels and stabilise membranes, enzymes and proteins. Osmolytes are mainly soluble carbohydrates, raffinose family oligosaccharides (RFOs), polyols, amino acids, amines and the amino acid derivatives betaines [16, 17].

One common early metabolic response to drought stress is the upregulation of RFOs such as raffinose, stachyose, and verbascose, followed by an increase in other sugars like fructose, galactose, sucrose, glucose, and erythritol [18]. Amino acids such as proline and the branched-chain amino acids (BCAAs) leucine, valine and isoleucine usually accumulate later. Proline is one of the most important amino acids accumulating in response to drought. It acts as an osmoprotectant and possibly also as a ROS scavenger [19–21]. Gamma-aminobutyric acid (GABA) is another non-protein amino acid that acts as an osmoprotectant and antioxidant and is linked to the tricarboxylic acid (TCA) cycle via the so-called GABA shunt [22]. Amines play a role in responding to drought and other stresses by counteracting senescence, exhibiting antioxidant properties, and stabilizing cell walls, membranes, and nucleic acids [19]. The most important polyamines for drought responses are putrescine, spermidine and spermine.

Such physiological changes can be measured qualitatively and quantitatively on different levels, e.g. on the genetic, transcriptomic, proteomic or metabolomic level, with the transcriptome, proteome, and metabolome serving as intermediaries between genotype and phenotype. These “endophenotypes” integrate lower-level effects and often correlate better with phenotypic traits [23]. They can be better suited in situations where the use of genetic markers can be challenging, e.g. in polyploids, with polygenic traits or with traits with strong epistatic effects. The predictive power of metabolic markers can be at least equal to that of genetic markers, making metabolomics a promising tool for studying stress responses [24, 25].

As phenotyping for stress tolerance is still a major bottleneck in breeding programs, screening for biomarkers to identify drought-tolerant lines would be greatly beneficial. Once a correlation between biomarkers and the trait of interest has been established, lines could be screened for these biomarkers before the phenotype becomes apparent, thus saving time and costs for setting up drought experiments.

Despite their potential, metabolic markers are sensitive to environmental influences, necessitating controlled conditions. Recent technological advances have led to the establishment of high-throughput phenotyping (HTP) facilities [26–28]. Equipped with high-resolution and spectral cameras, these facilities allow for automated and reproducible collection of imaging-based data under controlled environments with minimal manual labor. High-throughput phenotyping experiments, with their controlled conditions that reduce environmental noise, are particularly well-suited for transcriptomic and metabolomic studies [29].

This study investigated early-season drought stress responses in selected Nordic spring wheat genotypes to identify metabolites that could serve as biomarkers for drought tolerance and stability of yield-related traits in breeding programs. Twelve genotypes were grown under controlled conditions and exposed to early-season drought at the automated plant phenotyping platform for barley (APPP-B) facility at IPK Gatersleben, Germany. Plants were photographed daily and growth parameters such as height and biomass, as well as spectral data, were collected daily. After harvest, morphological and yield-related traits were assessed. Metabolic profiling was performed at four time points, including the post-drought recovery stage. This study provides a comprehensive analysis of metabolic responses to drought, offering valuable insights for breeding programs.

## Material and methods

### Spring wheat material

Forty-three Nordic spring wheat breeding lines and seven cultivars (PPPW_001 to PPPW_050) from breeding programmes from Sweden, Norway, Denmark, and Finland were selected based on their performance (yield relative to a reference genotype) in breeders’ fields during the 2018 drought (Additional file 1). These included both high- and low-yielding genotypes. To ensure uniform seed quality and age, all genotypes were multiplied in a controlled greenhouse environment at the Swedish University of Agricultural Sciences (SLU) in Alnarp, Southern Sweden, in 2020. Days to heading and flowering were recorded for each plant as the number of days from sowing until 50% of the spike was visible and flowering occurred, respectively, on the second tiller.

### Drought-stress experiment at the APPP-B

The experiment was conducted on the APPP-B (LemnaTec-Scanalyzer 3D system; LemnaTec GmbH, Aachen, Germany) in a climate-controlled greenhouse at the Leibniz Institute of Plant Genetics and Crop Plant Research (IPK), Gatersleben, Germany (51°4902300 N, 11°1701300 E, altitude 112 m). Plants were randomized and photographed daily in an imaging chamber equipped with top and side-view cameras (0°, 45°, and 90°) and a balance-watering station for precise drought control.

**Fig. 1:**
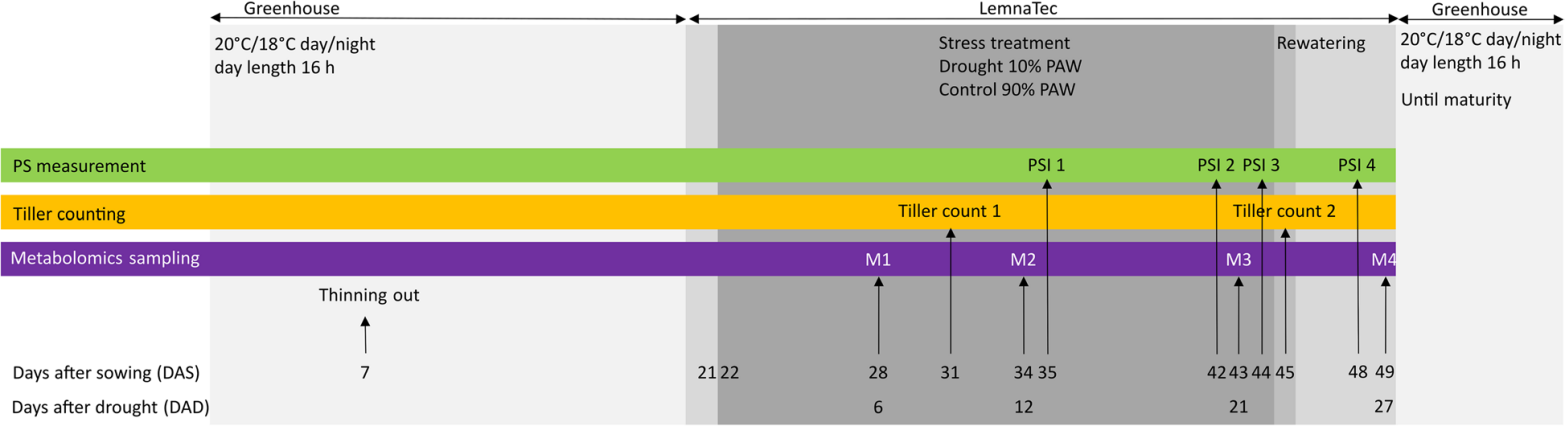
Experimental setup of the drought stress experiment. Medium grey indicates the phase of daily imaging in the APPP-B facility (21–49 days after sowing, DAS), dark grey indicates the drought stress phase (22–44 DAS), light grey the entire duration of the experiment. In the first experiment, photosynthetic measurements were taken at 41 DAS, and metabolite profiling was not performed

The drought experiment followed an established setup, ensuring a critical level of stress for effective genotype differentiation [30] (Fig. 1). Plants were sown in 2L pots (two seeds/pot) containing an equal weight of standard garden soil (Klasmann-Deilmann GmbH, Geeste, Germany). At seven days after sowing (DAS), pots were thinned to one plant per pot. Pre-cultivation was done for three weeks in a greenhouse at 20°C/18°C day/night temperature and a 16-h photoperiod with manual watering three times weekly.

At 21 DAS, plants were transferred to the phenotyping platform with the same temperature and light conditions as before, 7 g of long-term fertilizer with a composition of 19% total nitrogen, 9% P_2_O_5_ and 10% K_2_O and a plant support was added to each pot, and plants were automatically watered to 70% plant available water (PAW) [30]. The imaging and drought stress experiment started at 22 DAS (= 1 day after the start of drought treatment, DAD). Control plants were watered up to 90% PAW, while the PAW watering threshold of drought-treated plants was reduced to 10%. The stress treatment lasted until 45 DAS (24 DAD), after which plants were re-watered with 300 ml water first and then to 90% PAW from 46 DAS (25 DAD) onwards, like the control plants. Imaging ended at 49 DAS (28 DAD), and plants were transferred back to the greenhouse chamber at 50 DAS (29 DAD) until maturation and harvest.

### Pre-evaluation and time-course study

A pre-screening with 50 genotypes was performed to evaluate the entire panel during well-watered and drought conditions for the selection of a subset of 12 lines for a time-course study. In the pre-screening, five replicates per genotype and treatment were evaluated for imaging and post-harvest traits described below. Due to time constraints on access to the phenotyping platform, it was not possible to analyse all post-harvest traits from the pre-screening before starting the time-course study. Consequently, the twelve genotypes were selected based on the following criteria: (1) similar flowering time, (2) high or low biomass at the end of the drought period, 3) high or low levels of PAW at 44 DAS (23 DAD) under drought, and 4) high or low tiller number gain during drought (Additional file 1).

In the time-course study, 20 and 23 replicates per genotype were grown under the same conditions as the pre-screening study under control and drought conditions, respectively. Leaf samples were collected at four time points from plants that had not been sampled at previous time points (M); M1—early drought (7 DAD, stressed plants at on average 48.4% PAW), M2—mid drought (13 DAD, 28.3% PAW), M3—severe drought (22 DAD, 10% PAW) and M4—the fourth day of the recovery phase (28 DAD, previously stressed plants at 90% PAW). Five replicates per genotype were sampled at all time points under control treatment and at M1 and M4 under stress treatment. However, to account for greater environmental variability during more severe drought, six and seven replicates per genotype were sampled under drought conditions at M2 and M3, respectively. Samples were collected from the youngest fully developed leaf of the main tiller, which corresponded to the flag leaf in some genotypes. Each sample was immediately frozen in liquid nitrogen, homogenized, aliquoted into 2 mL Eppendorf tubes, and stored at − 80 °C until further use.

### Traits evaluated

Imaging was conducted as earlier described [30, 31] to measure spectral biomass as the primary trait, along with plant height, width, and compactness (Additional file 2, Additional file 3, Additional file 4, Additional file 5). Images were analysed using IAP (Integrated Analysis Platform) version 2.3.0 [32]. From the side and top view areas, a digital biomass volume (expressed in voxels) was calculated, referred to hereafter as"biomass"[33]. Additionally, various colour ratios (red, yellow, and brown to green) representing the percentage of red, yellow, or brown pixels relative to green pixels in the images were quantified to assess stress-induced changes in plant colour.

Photosynthesis measurements were performed at four time points using a Pulse Amplitude-Modulated (PAM) fluorometer (Photon Systems Instruments, PSI, Czech Republic), at 14 DAD (stressed plants at on average 26% PAW), 21 DAD (10% PAW), 23 DAD (10% PAW), and 27 DAD (three days after re-watering) and analysed using the manufacturer’s software Plant Data Analyzer (version 3). This instrument measures chlorophyll fluorescence from the top to evaluate the quantum yield (QY) of photosystem (PS) II (Φ_PSII_) [34]. To measure the efficiency of PSII under different light intensities, the plants were first adapted to a high light intensity (800 µm m^−2^ s^−1^, QY-Lss1) for five minutes prior to a saturating light flash of 4000 µm m^−2^ s^−1^. For the second measurement the plants were adapted to low light intensity (80 µm m^−2^ s^−1^, QY-Lss2) before a second flash of 4000 µm m^−2^ s^−1^. The ratio of the two QYs was calculated to assess the adaptability (plasticity) of PSII to different light intensities (Ratio_QY_LH) [31].

Tiller number was counted at 31 DAS (9 DAD) (TillerNumber1) and 45 DAS (24 DAD) (TillerNumber2), with the difference between the two time points recorded as TillerNumberGain. At maturity, the plants were evaluated for height and yield-related traits, including total plant weight, spike weight, number of spikes per plant, number of fertile and infertile spikes per plant, and the number of grains per plant. Additional traits, including thousand grain weight (TGW), grain area, grain length, and grain width, were measured using a Marvin Seed Analyzer (GTA Sensorik GmbH, Neubrandenburg, Germany).

### Metabolite analysis

Flag leaves were flash frozen in liquid nitrogen and ground in 10 mL plastic tubes together with a grinding ball for 2 min per sample in automatic Labman’s cryogenic grinding system (Labman, North Yorkshire, UK). Polar and semipolar metabolites were extracted from 15 mg of deep-frozen homogenized plant material using a polar metabolite extraction protocol according to Riewe et al. [35]. Extraction proceeded by adding 1 mL of chilled extraction buffer (2.5:1:1 v/v MeOH/CHCl_3_/H_2_O) containing 1 μL of a 2 mg mL^−1^ stock solution of ^13^C-sorbitol, and D4-alanine to the flash frozen and pulverized tissue. Following 15 min incubation at 4 °C, 0.4 mL H_2_O was added and centrifuged for 15 min at 4 °C and Vmax. Extraction was split into three batches and aliquots of 50 μL of polar phase into GC glass vials. Additionally, GC vials containing aliquoted samples were placed in a Speedvac overnight, crimped, and stored in sealed plastic bags with silica gel at −80 °C until analysis. Dried extracts were in-line derivatized directly prior to injection [36] using a Gerstel MPS2-XL autosampler (Gerstel, Mühlheim/Ruhr, Germany) with the front inlet temperature set at 200 °C, analysed in splitless mode. The analysis was performed on a LECO Pegasus BT time-of-flight mass spectrometer (LECO, St. Joseph, MI, USA) connected to an Agilent 8890 gas chromatograph with helium as the carrier gas at 1.0 mL min^−1^ flow and linear velocity as flow control mode. The capillary column used was an Agilent DB-35MS (30 m × 0.25 mm × 0.25 μm). Fatty acid methyl esters (FAME MIX: C8-C30) were used in the determination of the retention time index [37].

### Data processing and statistical analysis of phenotypic traits

The R package ‘StatgenHTP’ v1.0.5 [38] was used to remove outliers from the image-based traits, both at individual time points and across time points within each treatment. Due to the potential misclassification of sudden changes in phenotypic values caused by rewatering as outliers, filtering was applied only until 22 DAD. For all traits except biomass, the filtering parameters were set as follows: confIntSize = 5 and nnLocfit = 0.5. For biomass, filtering was conducted using confIntSize = 7 and nnLocfit = 0.55. Outlier removal was conducted in two rounds. Subsequently, data points exceeding two standard deviations for each genotype × trait × day × treatment combination were excluded. Plants with more than 20% of data points (= 5 days) filtered out across time points for any given trait were removed entirely for that particular trait. For post-harvest traits, data outside two and three standard deviations in the pre-screening and time-course experiment were excluded, respectively. After outlier removal, Spearman’s rank correlation coefficient (ρ) and broad sense heritability across experiments (H^2^) were calculated in R. H^2^ was calculated as follows:

$${H}^{2}=\frac{{\upsigma }_{g}^{2} }{({\upsigma }_{g}^{2}+\frac{{\upsigma }_{gxe}^{2}}{e}+\frac{{\upsigma }_{E}^{2}}{re})}$$

with σ^2^_g_ being the genetic variance, σ^2^_(g_ _×_ _e)_ the genotype-by-experiment interaction, σ^2^_E_ the error variance, *e* the number of experiments and *r* the number of replicates.

In the pre-screening, grain size traits were assessed separately for the main tiller and the remaining tillers, whereas in the time-course experiment these traits were evaluated for the entire plant. Consequently, Spearman’s ρ and H^2^ could not be calculated across experiments for these traits.

A two-way analysis of variance (ANOVA) was performed to determine the effect of genotype, drought and the genotype × drought effect on phenotypes. To evaluate trait stability under both control and drought, the relative loss of each trait under drought relative to control was calculated as follows:

$$\mathrm{Loss}\;\mathrm{of}\;\mathrm{trait}\;\left[\%\right]=\left(1-\frac{\mathit t\mathit r\mathit a\mathit i{\mathit t}_{\mathit d\mathit r\mathit o\mathit u\mathit g\mathit h\mathit t}}{\mathit t\mathit r\mathit a\mathit i{\mathit t}_{\mathit c\mathit o\mathit n\mathit t\mathit r\mathit o\mathit l}}\right)\times10$$

### Data processing and statistical analysis of metabolic data

Polar metabolite features were identified and annotated using LECO ChromaTOF software, which includes the Statistical Compare package and the electron impact spectra library from the Golm Metabolome Database (GMD, gmd.mpimp-golm.mpg.de). Metabolite feature intensities were normalized for fresh weight, internal standards, and individual detector responses to correct for potential extraction batch and measurement day effects using the R-package ‘TargetSearch’ [39]. Only features showing a > twofold change in leaf samples relative to blank samples were kept (Additional file 6).

Unless otherwise specified, data pre-processing and statistical analysis of the normalized, outlier-corrected data were conducted in R [40]. Using the R package ‘MetaboAnalystR’ [41], metabolites quantified in fewer than 80% of the samples were removed, and missing metabolite data were imputed with a value of 1/5 of the minimum value for the respective metabolite in the dataset. The 25% metabolites with the highest relative standard deviation were removed. The remaining data were median normalized, log_10_-transformed and Pareto-scaled to approximate a normal distribution.

The general effect of drought stress on the metabolome was analysed using the ‘prcomp()’ function in R and visualized in Principal Component Analysis (PCA) plots. A t-distributed stochastic neighbor embedding (t-SNE) analysis was performed using the R package ‘Rtsne’ v0.17as an additional, independent method to reduce the dimensionality of the metabolomic data for visualization [42, 43].

Differentially accumulated metabolites between treatments, sampling time points, or genotypes were identified using row-normalized data generated in ‘MetaboAnalystR’. Statistical significance was determined using Student’s unpaired t-test with a False Discovery Rate (FDR)-adjusted *p*-value < 0.05, assuming equal variances. Fold changes (FC) in metabolite peak intensities were calculated by dividing values under drought by values under control conditions, followed by log_2_-transformation. Orthogonal partial least squares discriminant analysis (OPLS-DA) was conducted using ‘MetaboAnalyst’ v6.0 [44] to identify metabolites associated with the differentiation between treatments or genotypes. Metabolites contributing most strongly to the OPLS-DA model were identified based on their variable influence on projection (VIP) scores. Metabolite names were matched against databases using the Chemical Translation Service [45] to retrieve their Kyoto Encyclopedia of Genes and Genomes (KEGG) terms. Pathway enrichment analysis was performed using the R package ‘FELLA’ [46], which identifies enriched pathways using KEGG database entries.

### Identification of metabolic markers correlated to phenotypic traits

Spearman’s ρ and R^2^ were calculated to identify significant correlations (*p* < 0.001) between phenotypes and metabolite peak intensities under drought or control conditions at 22 DAD. Partial least square regression (PLSR) was performed using the R package ‘pls’ [47] with a fourfold cross-validation to evaluate the predictive ability of metabolites for phenotypes. The twelve genotypes were divided into training (*n* = 9) and test sets (*n* = 3). Partial least square regression was used to predict phenotypes in the test set based on the metabolite data. Metabolites were ranked according to their regression coefficients in each iteration. This process was repeated until phenotypic values for all genotypes were predicted, and the rank product of each metabolite was calculated across iterations. Metabolites with a low rank product were considered good predictors of the respective phenotype. Only plants for which both phenotypic and metabolic data was available at 22 DAD (hence, five control-treated and seven drought-treated plants per genotype) were used for the Spearman’s ρ, *R*^2^ and PLSR analyses.

## Results

### Data quality across experiments

To evaluate imaging data quality, H^2^ and Spearman’s *ρ* values across experiments were calculated for the twelve genotypes analysed in both experiments (Additional file 7, Additional file 8). Overall, H^2^ exceeded 0.5 for most imaging-based traits, often reaching 0.8–0.9 (Additional file 7), with biomass, compactness, lab_b_mean and lab_l_mean showing the highest heritabilities (H^2^ > 0.7) on most days. Correspondingly, Spearman’s ρ for these traits exceeded 0.5 (Additional file 8). The hsv_h_red2green data from control conditions showed very low heritability early in the experiment, with H^2^ = 0 until 37 DAS (15 DAD) and negative Spearman’s ρ. The width measurements under drought showed negative correlations and very low H^2^ in the late drought phase.

Post-harvest traits such as SpikeNumber, TillerNumber1, TillerNumber2, and TillerNumberGain demonstrated strong repeatability, with Spearman’s *ρ* > 0.89 under control conditions and slightly lower values (> 0.76) under drought (Additional file 9). Heritabilities for these traits were high (H^2^ > 0.79). Most traits had H^2^ and Spearman’s *ρ* values above 0.7, with a few exceptions, such as SpikeWeight, InfertileSpikes, Length_SpikeBase_FlagLeaf, FertileGrains, and PeduncleLength. A malfunction in the watering system during the second experiment briefly reduced the percentage PAW for control plants to 64% at 14 DAD, though levels quickly recovered (Additional file 10). A slight reduction in percentage PAW (70%) for control plants was also noted at 13 DAD, coinciding with the third metabolic profiling time point. Overall, the data quality was high despite minor technical challenges.

For subsequent analyses, only the data from the time-course experiment, for which metabolic data was available, were considered.

### Effects of treatment and genotype on phenotypes

Except for awn length, drought treatment significantly affected all 38 imaging-based and post-harvest phenotypes (Table 1, Additional file 11). Biomass showed the most pronounced drought effect, with significant differences emerging at 9 DAD (*p* < 0.05, Additional file 10, Additional file 11). Most image-based traits started to show treatment-specific differences between 9 DAD (lab_l_mean) and 20 DAD (hsv_h_red2green). Biomass loss averaged 62.4% on the last day of drought (44 DAS, 23 DAD) and even increased further to 65–71% post-rewatering (Additional file 12). Height decreased by 16% at 44 DAS and recovered slightly to 14.6% loss at 49 DAS (28 DAD). Several image-based traits (hsv_h_yellow2green, hsv_h_mean, lab_l_mean, lab_a_mean, lab_b_mean) fluctuated significantly around late drought and rewatering, complicating interpretation of these values.

**Table 1:** Analysis of variance (ANOVA)

**Trait**	**Genotype**	**Treatment**	**Genotype x Treatment**
Biomass^*^	2.81E-48	3.98E-159	5.81E-29
TillerNumberGain	1.73E-73	1.10E-123	5.01E-17
hsv_h_brown2green^*^	5.74E-44	4.58E-119	3.61E-08
TillerNumber2	1.25E-86	2.58E-116	1.10E-14
PlantWeight	3.97E-38	9.45E-83	0.0985
TGW	1.38E-129	2.08E-80	1.62E-24
Width^*^	1.21E-05	1.48E-79	4.71E-06
lab_a_mean^*^	3.38E-89	8.00E-79	0.0042
GrainDensity	4.40E-26	4.92E-72	0.0821
GrainWeight	4.49E-26	5.06E-72	0.0824
Height^*^	5.82E-41	1.13E-70	6.17E-07
lab_b_mean^*^	6.68E-107	5.81E-64	0.00316
GrainArea	2.05E-128	1.64E-53	1.06E-27
GrainNumber	3.64E-102	5.41E-47	0.0059
GrainWidth	1.75E-135	1.02E-41	3.68E-33
RachisNodeNumber	3.07E-54	4.99E-40	0.0162
hsv_h_yellow2green^*^	8.51E-41	3.80E-37	1.10E-05
GrainLength	1.16E-163	9.75E-34	0.0288
NumberFertileSpikes	1.02E-44	1.12E-33	0.0313
hsv_h_red2green^*^	6.36E-12	5.60E-30	2.06E-07
FertileGrains	5.93E-42	1.27E-28	0.000598
hsv_h_mean^*^	7.88E-39	1.52E-27	0.0248
SpikeWeight	3.37E-41	2.90E-24	0.00179
SpikeNumber	1.21E-67	1.08E-23	0.155
Compactness^*^	7.06E-73	1.01E-19	0.0642
PH_TopSpike	2.74E-73	1.09E-16	0.000397
InfertileSpikes	8.75E-13	2.25E-15	0.00117
PH_BaseSpike	4.21E-60	1.85E-12	0.00297
SpikeLength	4.38E-108	7.90E-12	0.0138
PH_FlagLeaf	7.36E-75	5.21E-11	0.0225
GrainLengthWidth	7.72E-181	4.77E-08	1.10E-17
TillerNumber1	8.71E-32	8.47E-06	0.447
SpikeDensity	2.37E-72	1.08E-05	0.67
PH_TopNode	2.28E-60	2.84E-05	0.0831
lab_l_mean^*^	6.22E-85	0.000413	0.245
SpikeCulmRatio	3.50E-79	0.0147	0.075
InfertileGrains	5.90E-14	0.0422	8.98E-08
AwnLength	2.58E-167	0.0639	0.000595

^*^Imaging data corresponds to day 23 after the onset of drought (end of drought period)

**Fig. 2:**
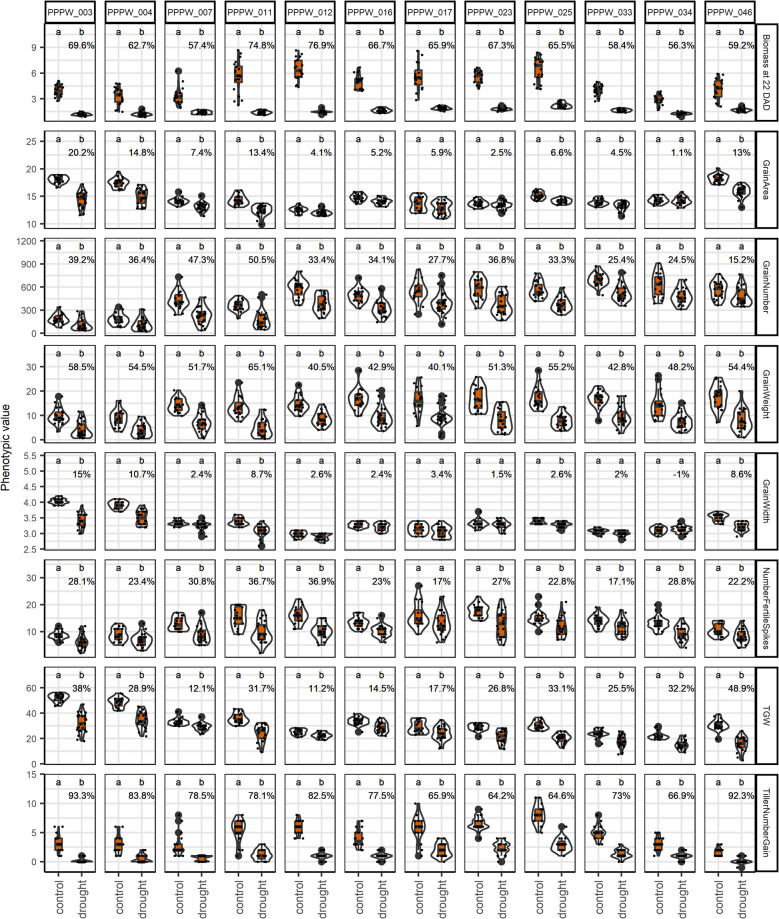
Genotype-wise response of selected phenotypes to treatments. Letters indicate significantly different means at *p* < 0.01 (Tukey’s honest significance test). Percentages represent trait loss relative to control. Phenotypic units are provided in Additional file 2. Values for biomass are shown in millions

**Fig. 3:**
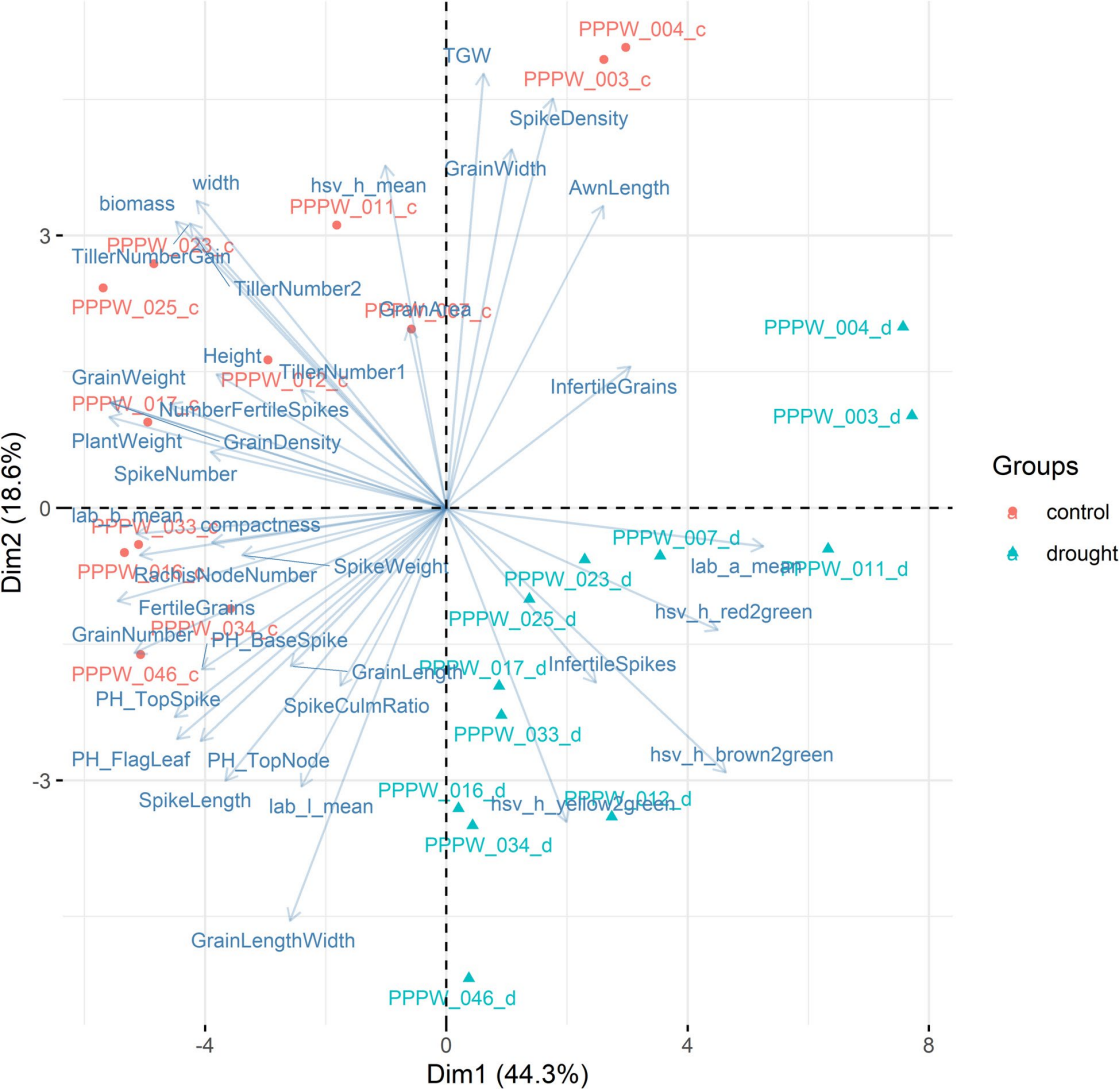
Biplot of genotype-wise treatment effect on phenotypes at 22 days after the onset of drought. Arrows represent phenotypes, with length and direction indicating their influence. Phenotypes perpendicular to treatment separation indicate strong treatment correlations

Among post-harvest phenotypes, TillerNumberGain, TillerNumber2, PlantWeight, TGW, and grain size and shape traits showed the strongest treatment effects (Table 1, Fig. 2). Biomass reduction under drought ranged from 56 to 77% at 22 DAD, while TillerNumberGain declined by 64–93%. GrainWeight and TGW were more severely impacted than NumberFertileSpikes and GrainNumber. No genotype demonstrated superior performance across multiple key traits under drought. Genotypic effects were significant for all phenotypes, particularly GrainLengthWidth, AwnLength, and GrainLength. Figure 3 illustrates that biomass, tiller traits, SpikeNumber, GrainWeight, PlantWeight, and colour-to-green ratios had a strong impact on the differentiation by treatment. This is evident from the direction of arrows representing phenotypes perpendicular to the treatment differentiation.

Grain and tiller traits, including GrainWidth, GrainArea, TGW, GrainLengthWidth, TillerNumberGain, and biomass, exhibited the strongest genotype-by-treatment interactions, indicating that the drought severity effects on these traits were highly genotype-dependent.

Interestingly, no significant drought impact on PSII plasticity was observed in most genotypes, even under severe drought at 22 DAD (Additional file 13).

### Accumulation of known drought-responsive metabolites over time

For biological validation of the experiment, we examined the temporal patterns of known key metabolites implicated in drought responses (Additional file 14). As expected, proline and nicotinamide levels rose during drought and dropped sharply upon rewatering, with control plants exhibiting similar but less pronounced trends. Putrescine levels were consistently lower under drought than control conditions and declined over time in most genotypes, while control plants showed no clear pattern. Histamine and spermidine decreased markedly during drought, and histamine increased slightly during recovery, while spermidine continued to decrease. In control plants, histamine levels increased over time, while spermidine remained relatively stable. Spermine levels increased slightly during drought in most genotypes, but were not significantly different from control plants. Raffinose, one of the earliest metabolites to accumulate under drought, showed genotypic differences in accumulation. Its levels decreased during recovery in most genotypes, though high raffinose levels were also observed in control plants, suggesting a role beyond stress-specific responses.

### General metabolic responses to drought over time

The metabolic response to drought was studied at four time points: 7, 13, and 22 DAD, and four days after rewatering (28 DAD). After data processing and filtering, 173, 177, 172, and 169 metabolites were retained for 7, 13, 22, and 28 DAD, respectively, with 167 metabolites present across all time points.

**Fig. 4:**
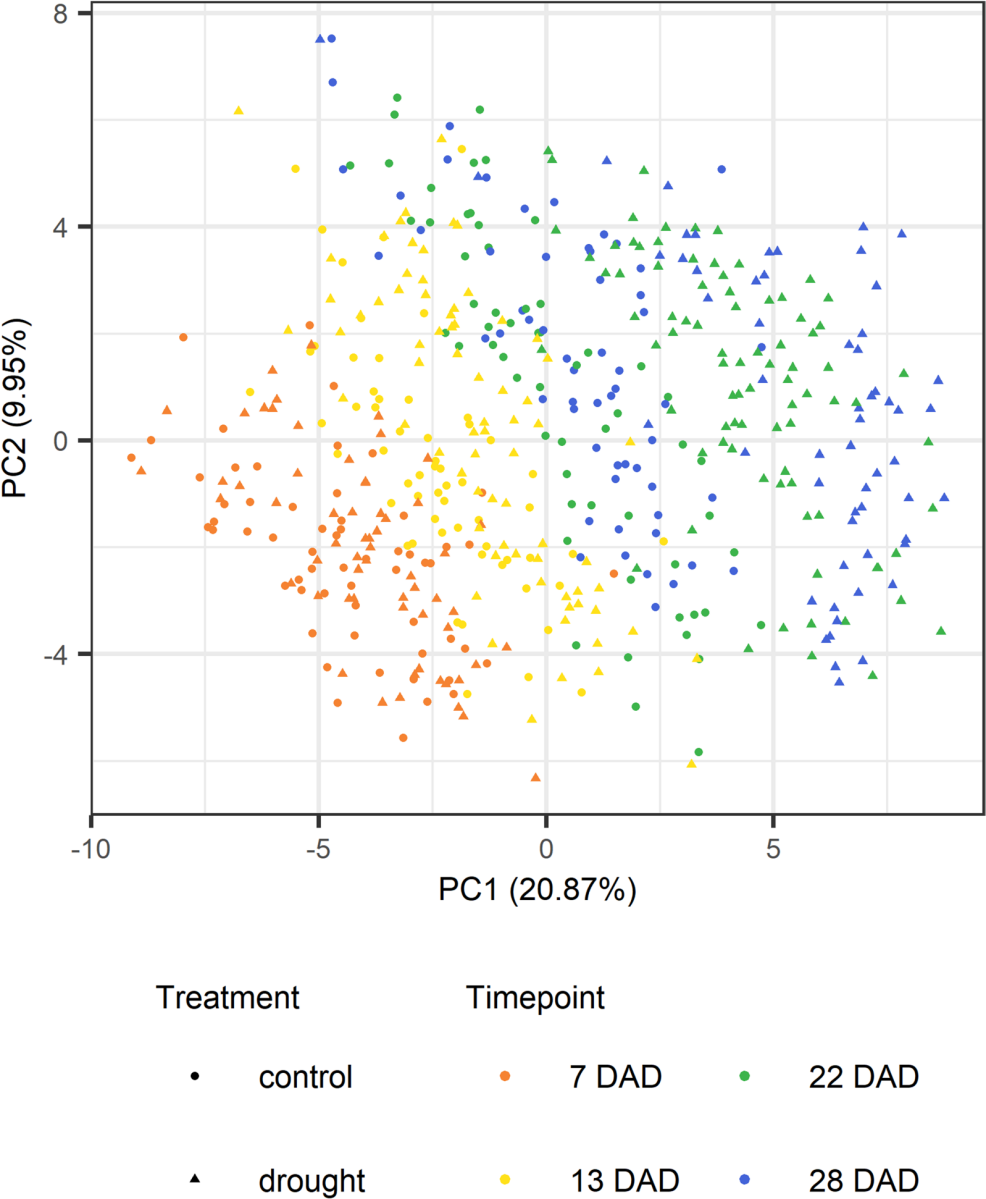
Principal component analysis (PCA) of metabolic profiles by plant and treatment at four time points. Samples were taken at 7, 13, 22, and 28 days after the onset of drought (DAD)

**Fig. 5:**
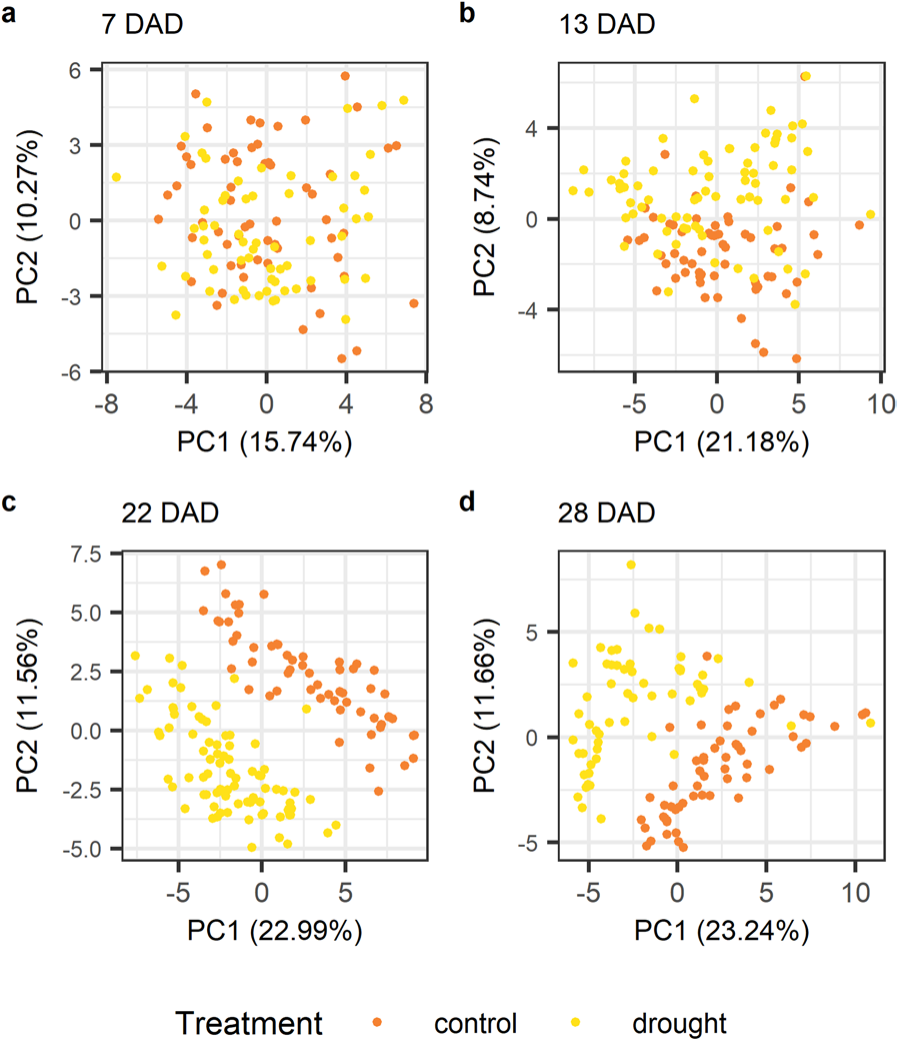
Principal component analyses (PCAs) of metabolic profiles by plant and treatment. **a** 7 days after the onset of drought (DAD), **b** 13 DAD, **c** 22 DAD and **d** 28 DAD

Principal component analysis (PCA) visualizations of metabolic profiles revealed temporal changes (Figs. 4 and 5). Samples from 7 and 13 DAD formed distinct adjacent clusters, while 22 and 28 DAD samples overlapped but clustered clearly by treatment. Metabolic differences between treatments emerged at 13 DAD, as samples started to form two distinct clusters, suggesting that some genotypes may respond to drought earlier than others. By 22 DAD, clustering by treatment was complete. Notably, even six days after rewatering (28 DAD), the clusters remained distinct, indicating that drought-induced metabolic changes persist beyond the resumption of normal watering.

The timing of observable metabolic changes (13 DAD) corresponded closely with the onset of significant differences in biomass accumulation between drought-stressed and control plants (9 DAD) (Additional file 10).

The t-SNE analysis (Additional file 15) confirmed the temporal pattern and the effect of drought stress on the metabolome becoming apparent between 13 and 22 DAD. Also the lasting effect after recovery was noticeable. In addition, it showed that although there is, as expected, some variation, biological replicates of the same genotype tended to cluster together, especially at 22 DAD. While at 7 DAD biological replicates of the same genotype cluster together regardless of the treatment effect, genotype clusters are separated by treatment effect at 22 DAD, and to some extent, even after rewatering.

### Differentially accumulated metabolites between control and drought-treated plants

**Fig. 6:**
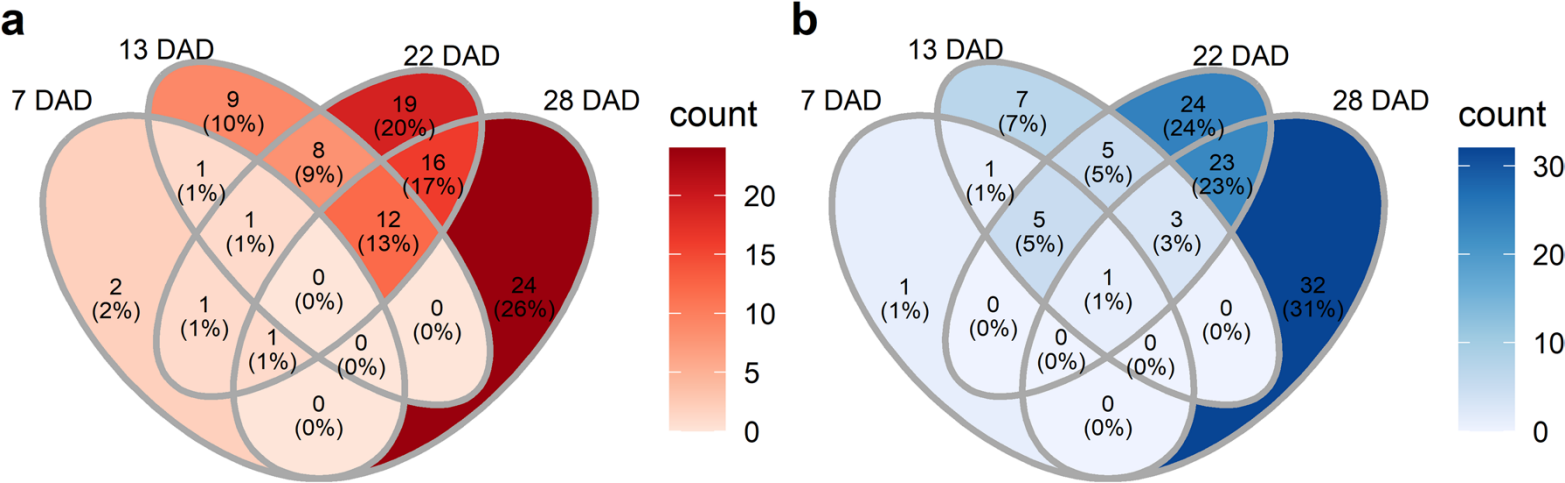
Venn diagrams depicting the number of differentially accumulated metabolites at each time point. Shown are the number of metabolites that **a**) increased and **b**) decreased under drought at different time points 7, 13, 22, and 28 days after the onset of drought, DAD). Colors indicate metabolite counts in each section

**Fig. 7:**
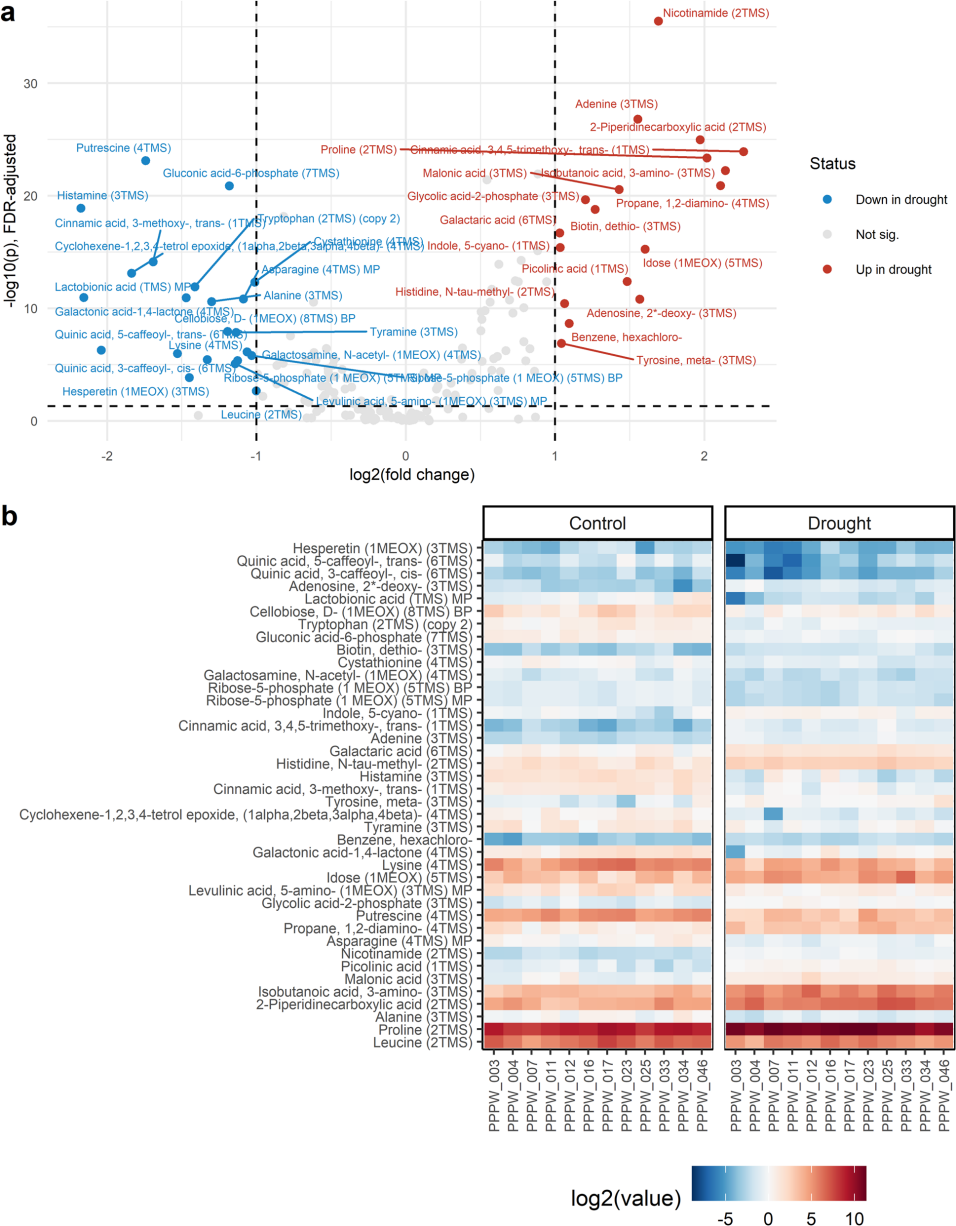
Metabolic responses to drought at 22 days after the onset of drought (DAD). **a** Volcano plot showing differentially accumulated metabolites (DAMs) accumulated (red) reduced (blue) under drought compared to control conditions (T-test, FDR-adjusted *p* < 0.05, −1 < fold change < 1). **b** Log_2_-transformed, row-normalized metabolite intensities

**Fig. 8:**
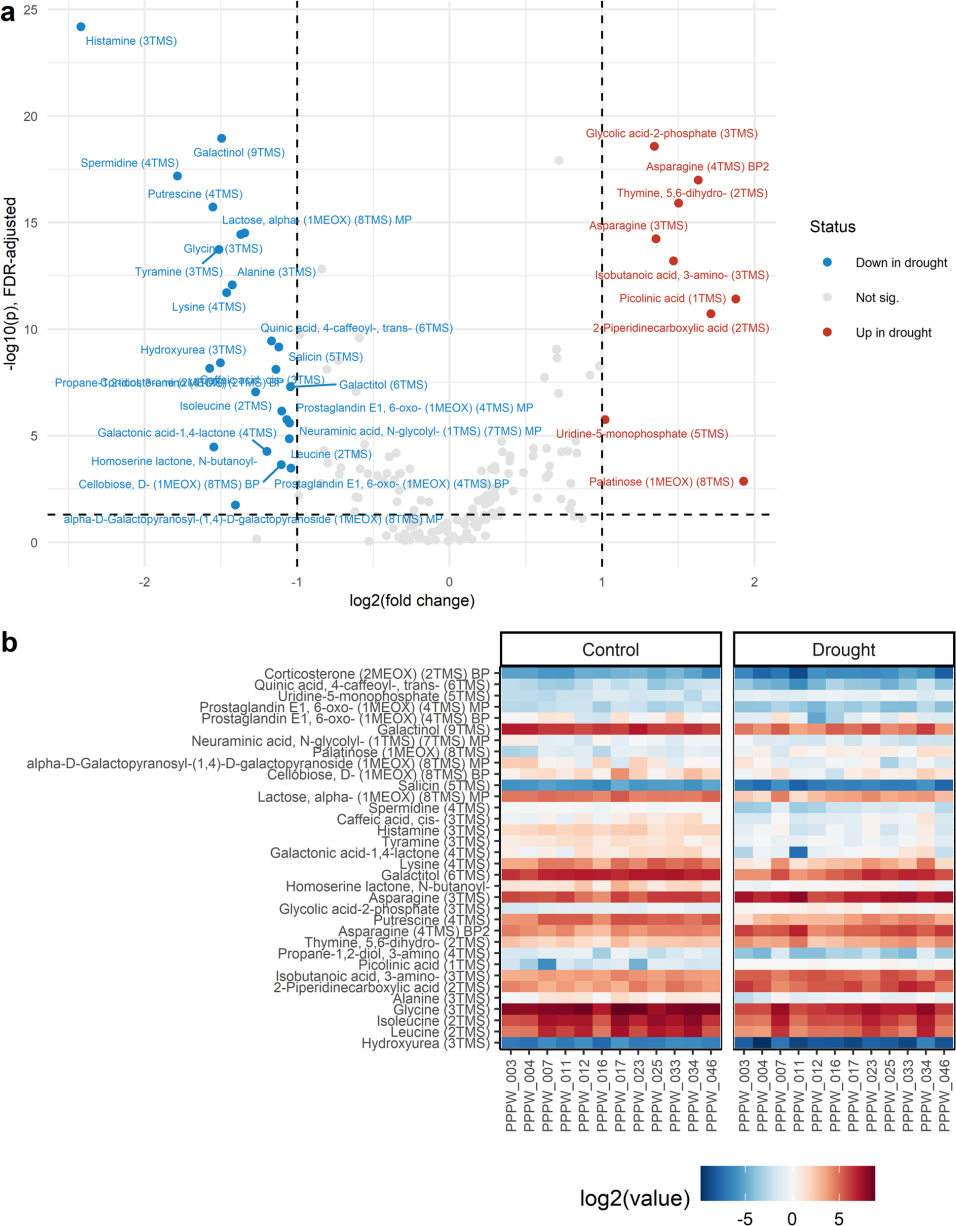
Metabolic responses to drought after rewatering (28 after the onset of drought, DAD). **a** Volcano plot showing differentially accumulated metabolites (DAMs) accumulated (red) reduced (blue) under drought compared to control conditions (T-test, FDR-adjusted *p* < 0.05, −1 < fold change < 1). **b** Log_2_-transformed, row-normalized metabolite intensities

**Fig. 9:**
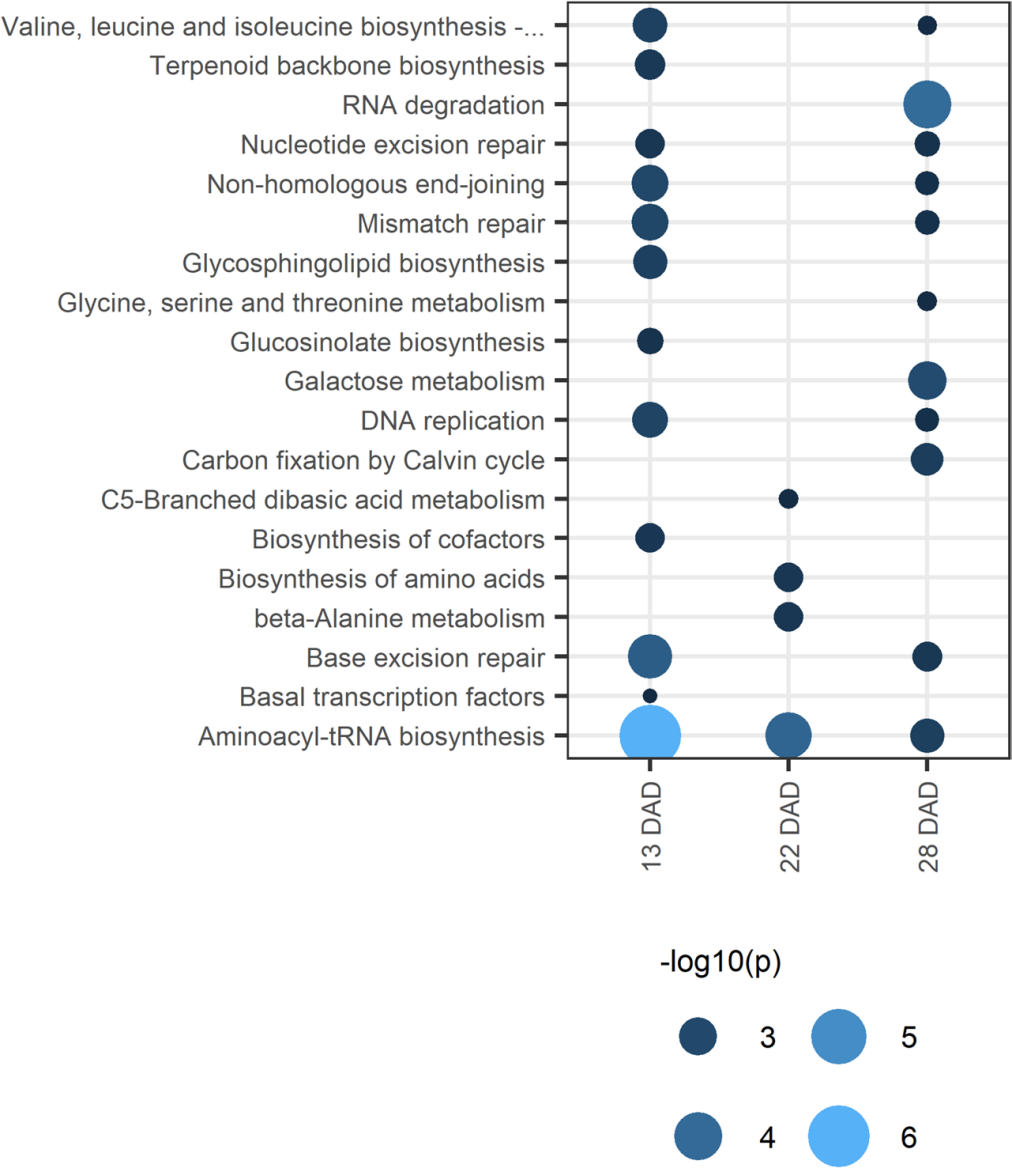
Pathway enrichment at 13, 22, and 28 after the onset of drought (DAD). Shown are pathways that were significantly enriched at *p* < 0.01. Note: Enrichment of glucosinolate pathways reflects KEGG database misannotations, as wheat does not produce glucosinolates

Metabolites showing a significant response to drought were identified using Student’s unpaired t-test, with FDR < 0.05 defining differentially accumulated metabolites (DAMs) (Additional file 16). The number of DAMs was 14 at 7 DAD, 53 at 13 DAD, 119 at 22 DAD, and 112 at 28 DAD (Fig. 6). Early drought responses included increased peak intensities of 3-methylpentananoic acid, succinic acid (7 DAD) and spermine (7 and 13 DAD), alongside decreased peak intensities of psicose (7 DAD) and 4-hydroxyphenylacetonitrile (7 and 13 DAD) (Additional file 16, Additional file 17). Trans-aconitic acid accumulated consistently under drought at 7, 13, and 22 DAD, while 2-mercaptoethanesulfonic acid, asparagine, aminomalonic acid, and 5–6-dihydrothymine decreased at these time points, indicating their involvement in long-term drought responses. Putrescine was the only metabolite consistently reduced at all sampling time points. At 22 DAD, the most severe drought phase, the metabolites showing the highest fold changes between treatments included trans-3,4,5-trimethoxycinnamic acid, 3-aminoisobutanoic acid, and 1,2-diaminopropane (increased) and histamine, lactobionic acid, and trans-5-caffeoylquinic acid (decreased) (Fig. 7). Nicotinamide and adenine were most significantly accumulated, while putrescine and gluconic acid-6-phosphate were most significantly reduced. At 28 DAD, the largest fold changes were observed for palatinose, picolinic acid, and 2-piperidinecarboxylic acid (increased) and histamine, spermidine, and 3-aminopropane-1,2-diol (decreased) (Fig. 8). Glycolytic acid-2-phosphate, asparagine, and 5,6-dihydrothymine increased most significantly, while histamine, galactinol, and spermine decreased most significantly in plants previously exposed to drought. Notably, DAMs at 22 and 28 DAD showed greater overlap than those at 13 and 22 DAD, highlighting the persistence of drought-induced metabolic alterations after rewatering. Pathway enrichment analysis revealed significant changes in specific pathways. The aminoacyl-tRNA pathway was the only pathway consistently altered at 13, 22, and 28 DAD (*p* < 0.01). The pathways at 13 and 28 DAD overlapped and were primarily associated with RNA and DNA replication, degradation, and repair, whereas the pathways identified at 22 DAD were predominantly related to amino acid biosynthesis and metabolism (Fig. 9).

### Drought stress-dependent metabolic differences in high- and low-performing genotypes

**Fig. 10:**
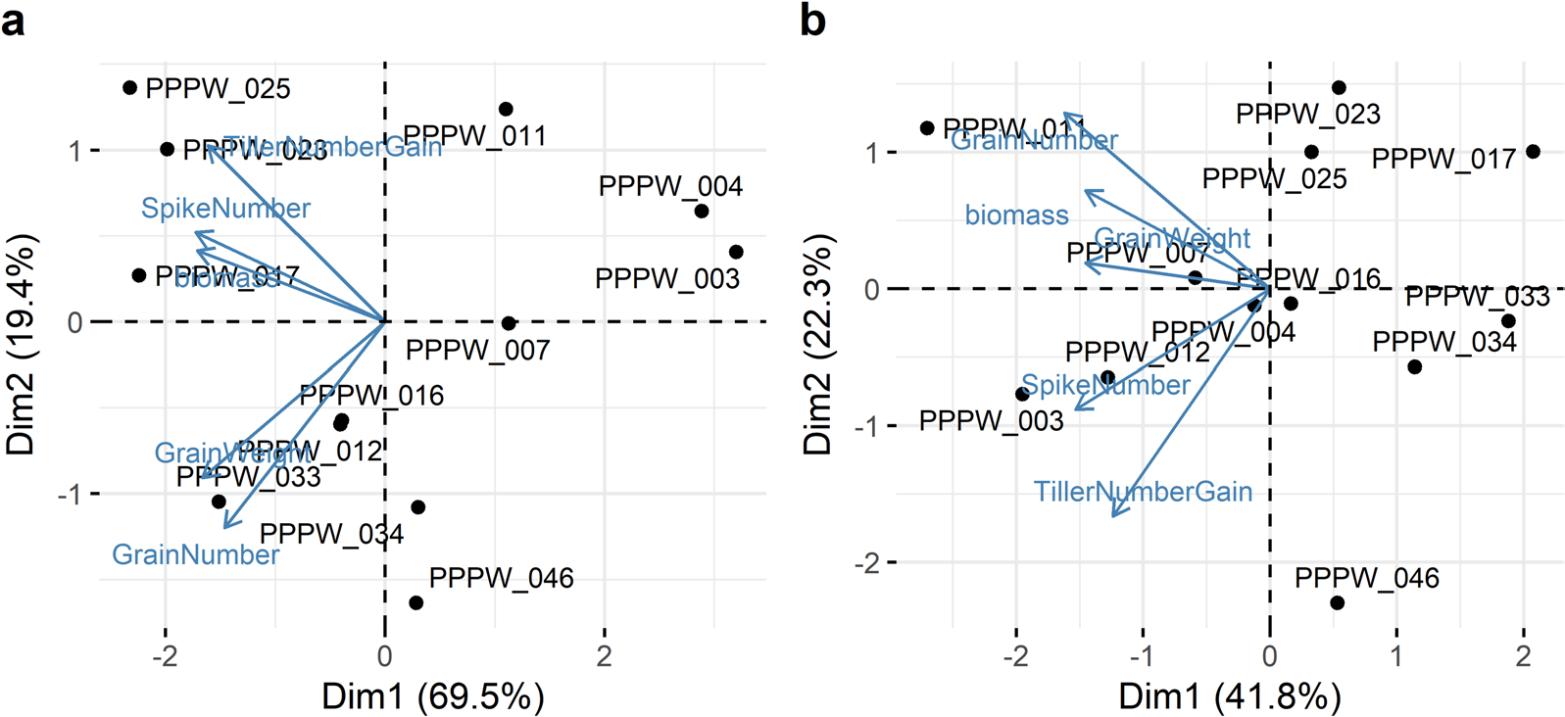
Criteria used for selection of tolerant and non-tolerant genotypes. a) Biplot of drought performance at 22 after the onset of drought (DAD), b) Biplot of % loss of trait at 22 DAD. Arrows represent phenotypes, with length and direction indicating their impact. Phenotypes perpendicular to treatment separation strongly correlate with treatment effects

As no genotypes exhibited clear superior performance in terms of yield-related traits under drought, we used three approaches to identify high and low-performing genotypes: First, we ranked genotypes by GrainWeight, GrainNumber, biomass at 22 DAD, TillerNumberGain and SpikeNumber and calculated the rank product. The highest performing genotypes were PPPW_017, PPPW_025, PPPW_033 and PPPW_023, while PPPW_003, PPPW_004, PPPW_007, and PPPW_011 were the lowest (Table 2). Next, the genotypes were placed in a PCA space based on their phenotypic values under drought, and high (PPPW_025, PPPW_023, PPPW_017, PPPW_033) and low (PPPW_003, PPPW_004, PPPW_011, PPPW_007) performing genotypes were identified based on their placement and the direction and value of the phenotypic effect vectors (Fig. 10a). The same approach was applied using the percentage loss in phenotypic values under drought compared to control conditions, where PPPW_017, PPPW_033 and PPPW_034 showed relatively stable performance, whereas PPPW_011, PPPW_003 and PPPW_012 showed larger losses (Fig. 10b). Based on these analyses, we defined PPPW_017 and PPPW_033 as drought-tolerant genotypes, and PPPW_007 and 011 as non-tolerant genotypes. We excluded PPPW_003 and PPPW_004 in this analysis due to their distinct phenotypic and metabolic profiles. These genotypes seem to produce larger but fewer grains than other genotypes, possibly due to a lower spike number as they exhibited shorter spikes, a lower rachis node number, and grain number, a higher spike density, a lower plant weight and grain weight, a relatively low spike number, a higher TGW, a higher grain width, a higher grain area and longer awns than most genotypes under both conditions (Additional file 3, Additional file 4). Additionally, the metabolic profile of these two genotypes was distinct from that of the other genotypes under control conditions already at 13 DAD, suggesting that their drought responses are potentially unrepresentative of the broader genotype pool (Additional file 18).

**Table 2:** Ranking of genotypes based on their performance under drought

**Genotype**	**GrainNumber**	Biomass^*^	**GrainWeight**	**TillerNumberGain**	**SpikeNumber**	**Total**
PPPW_017	4	2	1	3	2	1
PPPW_025	6	1	7	1	3	2
PPPW_033	1	5	2	4	4	3
PPPW_023	7	3	5	2	1	4
PPPW_012	5	7	4	6	5	5
PPPW_046	2	4	6	12	10	6
PPPW_016	8	6	3	8	7	7
PPPW_034	3	10	8	7	9	8
PPPW_011	10	8	10	5	6	9
PPPW_007	9	9	9	9	8	10
PPPW_004	11	11	11	10	11	11
PPPW_003	12	12	12	11	12	12

^*^Biomass (in voxels) at 22 days after the onset of drought

A per-genotype differential accumulation analysis revealed that six metabolites were differentially accumulated in the tolerant genotypes. Five of these (adenosine-5-monophosphate, trans-3,4,5-trimethoxycinnamic acid, glyceric acid, pyridoxal and sebacic acid) were increased under drought, while histamine was reduced (t-test at FDR < 0.05, OPLS-DA VIP > 1) (Table 3, Additional file 19a and Additional file 19b, Additional Table 20). In the two non-tolerant genotypes, glycerol-3-phosphate, hippuric acid, idose and 6-benzylaminopurine accumulated under drought, while fructose-6-phosphte showed reduced accumulation.

**Table 3:** Metabolites differentially accumulated in tolerant and non-tolerant genotypes under drought versus control conditions

	**Tolerant genotypes (PPPW_017, PPPW_033)**	**Non-tolerant genotypes (PPPW_007, PPPW_011)**
Accumulated	Adenosine-5-monophosphate (5TMS) BP Cinnamic acid, 3,4,5-trimethoxy-, trans- (1TMS) Glyceric acid (3TMS) Pyridoxal (1MEOX) (2TMS) MP Sebacic acid (2TMS)	Glycerol-3-phosphate (4TMS) Hippuric acid (2TMS) Idose (1MEOX) (5TMS) Purine, 6-benzylamino- (1TMS)
Decreased	Histamine (3TMS)	Fructose-6-phosphate (1MEOX) (6TMS) BP Fructose-6-phosphate (1MEOX) (6TMS) MP

Timepoint: 22 days after the onset of drought (FDR-adjusted *p*-value < 0.05; OPLS-DA VIP > 1)

A pairwise metabolite comparison of high performing vs. low performing genotypes under drought at 22 DAD (PPPW_033 vs. PPPW_007, PPPW_017 vs. PPPW_007, PPPW_033 vs. PPPW_011 and PPPW_017 vs. PPPW_011, respectively) revealed that N-danzyl-aziridine, 2'-deoxy-guanosine, and cis-3-caffeoylquinic acid accumulated more in the tolerant genotypes, while lactulose was reduced in the tolerant genotypes in three out of the four comparisons (Table 4, Additional file 19c and Additional file 19d).

**Table 4:** Differentially accumulated metabolites between tolerant and non-tolerant genotypes after 22 days of drought

**Number of pairwise analyses**	**Accumulated in tolerant genotypes**	**Decreased in tolerant genotypes**
4	Aziridine, N-dansyl-Guanosine, 2*-deoxy- (4TMS) Quinic acid, 3-caffeoyl-, cis- (6TMS)	-
3	-	Lactulose (1MEOX) (8TMS) MP

### Metabolites and metabolic pathways involved in recovery from drought stress

**Fig. 11:**
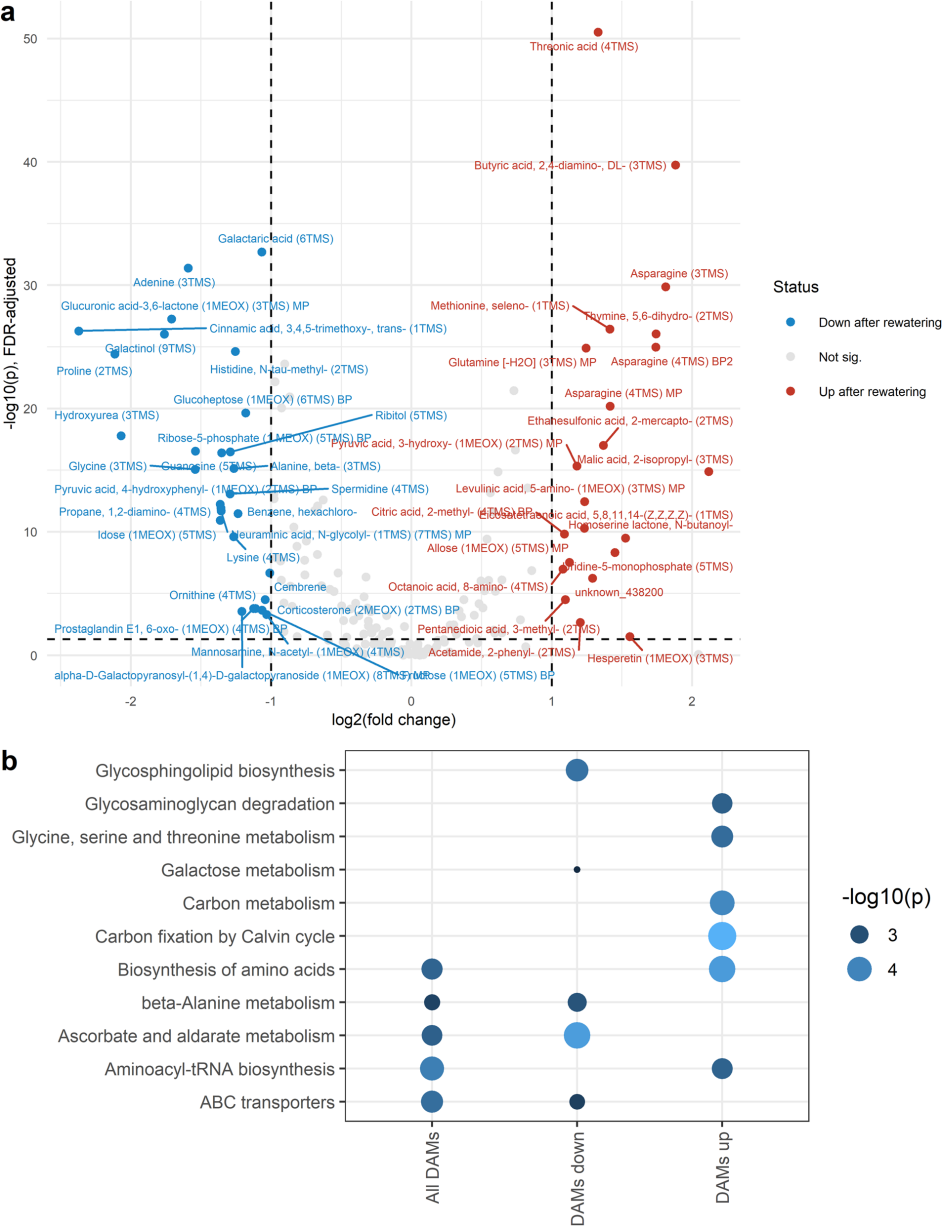
Metabolic responses to rewatering. **a** Volcano plot showing differentially accumulated metabolites (DAMs) accumulated (red) reduced (blue) at the rewatering stage (28 after the onset of drought, DAD) compared to drought at 22 DAD (T-test, FDR-adjusted *p* < 0.05, −1 < fold change < 1). **b** FELLA-based enrichment analysis of pathways. Dot size and color indicate pathway significance (-log_10_(*p*)). Note: Enrichment of glycosaminoglycan pathways reflects KEGG database misannotations, as plants do not produce glycosaminoglycans

Although many metabolites showed similar changes at both 22 DAD and 28 DAD, indicating a lasting impact of drought on parts of the metabolome, major changes occurred after rewatering. At 28 DAD, 49 metabolites were significantly enriched and 77 reduced compared to 22 DAD in drought-stressed plants, with highly significant *p*-values (-log10(FDR-adjusted *p*-value) < 50) (Fig. 11a, Additional file 21). The DAMs with the strongest fold changes were 2-isopropylmalic acid and cis-3-caffeoylquinic acid (log2(FC) > 2), and trans-3,4,5-trimethoxycinnamic acid, proline, and hydroxyurea (log2(FC) < −2). Threonic acid, DL-2,4-diaminobutyric acid, and asparagine showed the most significant increases after rewatering, while galactaric acid, adenine, and glucuronic acid-3,6-lactone were most significantly decreased.

The pathways associated with these DAMs were primarily linked to amino acid metabolism and biosynthesis, aminoacyl-tRNA biosynthesis, and ABC transporters (Fig. 11b). DAMs enriched after rewatering were associated with aminoacyl-tRNA biosynthesis, amino acid biosynthesis and metabolism, and carbon metabolism and fixation by the Calvin cycle, indicating that carbon metabolism had been compromised under drought and was starting to recover post-rewatering. Conversely, DAMs reduced after rewatering were linked to galactose metabolism, ascorbate and aldarate metabolism, glycosphingolipid biosynthesis, beta-alanine metabolism, and ABC transporters.

### Identification of metabolites correlated with phenotypic performance

The potential of metabolites to predict phenotypic performance under both control and drought conditions was assessed using correlation analysis and PLSR. Metabolite peak intensities at 22 DAD under both conditions were related to phenotypic values under the same condition, and to the relative changes between control and drought (loss of trait). A positive correlation between metabolites and phenotypes indicates that higher metabolite peak intensities correspond to higher phenotypic values, while a positive correlation between metabolites and loss of trait values suggests that higher metabolite peak intensities are associated with greater relative decreases in phenotypic value under drought, indicating lower phenotypic stability or reduced drought tolerance.

Table 5 summarizes the 39 significant correlations (*p* < 0.001) between metabolite peak intensities and selected phenotypes across four datasets: metabolite peak intensities under control vs. phenotypes under control (CC), metabolite peak intensities under drought vs. phenotypes under drought (DD), metabolite peak intensities under control vs. loss of trait (LC), and metabolite peak intensities under drought vs. loss of trait (LD). In total, 17 phenotypic traits were significantly associated with 32 metabolites in at least one dataset. These included key plant performance traits such as GrainNumber, GrainLength, GrainWidth, GrainArea, SpikeLength, SpikeDensity, biomass, and plant height. Grain size traits showed the highest number of associations (GrainArea: *n* = 5, GrainLength: *n* = 4, GrainWidth: *n* = 4). Notably, TGW was not associated with any metabolite at *p* < 0.001.

**Table 5:** Significant correlations between metabolite concentrations and selected phenotypes

Metabolite	Trait	Spearman’s ρ	*p* value	FDR-adj. *p* value	R^2^	Rank (PLS)^a^
CC (metabolite intensities under control vs. phenotypes under control)						
alpha-D-Glucopyranosyl-(1,6)-D-mannitol (9TMS)	SpikeLength	−0.90	*****	*****	0.7	1
Ribonic acid (5TMS)	PlantWeight	0.90	*****	**	0.8	2
alpha-D-Glucopyranosyl-(1,6)-D-mannitol (9TMS)	SpikeDensity	0.89	****	*	0.71	1
Raffinose (11TMS)	GrainNumber	−0.88	***	*	0.78	1
Urea (2TMS)	Compactness	0.88	***	*	0.83	1
alpha-D-Glucopyranosyl-(1,6)-D-mannitol (9TMS)	SpikeCulmRatio	−0.87	***	ns	0.75	1
Erythronic acid (4TMS)	PlantWeight	0.87	***	*	0.61	4
Propane, 1,2-diamino- (4TMS)	GrainArea	0.87	***	ns	0.41	5
Adenosine-5-monophosphate (5TMS) BP	GrainWidth	−0.84	***	ns	0.73	2
Isobutanoic acid, 3-amino- (3TMS)	AwnLength	−0.85	***	ns	0.63	8
alpha-D-Galactopyranosyl-(1,4)-D-galactopyranoside (1MEOX) (8TMS) MP	GrainNumber	−0.85	***	ns	0.43	9
alpha-D-Galactopyranosyl-(1,4)-D-galactopyranoside (1MEOX) (8TMS) MP	SpikeDensity	0.85	***	ns	0.54	6
Histamine (3TMS)	Width	−0.85	***	ns	0.51	4
Prephenic acid (1MEOX) (3TMS)	hsv_h_brown2green	−0.85	***	ns	0.49	3
DD (metabolite intensities under drought vs. phenotypes under drought)						
Raffinose (11TMS)	AwnLength	0.91	*****	*****	0.58	17
Gluconic acid, 2-amino-2-deoxy- (7TMS)	GrainArea	0.91	*****	*****	0.84	2
Putrescine (4TMS)	hsv_h_brown2green	−0.89	****	*	0.54	6
Biotin, dethio- (3TMS)	hsv_h_brown2green	−0.87	***	*	0.59	1
Guanosine, 2*-deoxy- (4TMS)	PlantWeight	0.87	***	ns	0.61	2
Shikimic acid (4TMS)	GrainLength	0.87	***	*	0.59	2
Orotic acid (3TMS)	GrainLength	0.87	***	*	0.57	4
Quinic acid (5TMS)	GrainLength	0.87	***	*	0.58	6
Anthranilic acid (2TMS)	PH_BaseSpike	0.86	***	ns	0.7	1
Tartaric acid (4TMS)	SpikeCulmRatio	0.86	***	ns	0.67	2
Fructose-6-phosphate (1MEOX) (6TMS) BP	SpikeWeight	0.86	***	ns	0.51	1
Trehalose, beta,beta*- (8TMS)	GrainArea	0.86	***	ns	0.59	9
Anthranilic acid (2TMS)	PH_TopSpike	0.85	***	ns	0.66	1
Fructose-6-phosphate (1MEOX) (6TMS) BP	PH_TopSpike	0.85	***	ns	0.58	2
LC (metabolite intensities under control conditions vs. loss of trait)						
Calystegine B2 (1MEOX) (4TMS)	PlantWeight	−0.87	***	ns	0.6	2
Xylose (1MEOX) (4TMS) MP	GrainArea	−0.87	***	*	0.82	1
Xylose, D- (1MEOX) (4TMS)	GrainArea	−0.87	***	*	0.86	2
Glucuronic acid (1MEOX) (5TMS) MP	Biomass	−0.87	***	ns	0.5	4
Ribose-5-phosphate (1 MEOX) (5TMS) MP	GrainWidth	−0.86	***	ns	0.78	7
Ribose-5-phosphate (1 MEOX) (5TMS) BP	GrainWidth	−0.86	***	ns	0.78	8
Xylose, D- (1MEOX) (4TMS)	GrainWidth	−0.85	***	ns	0.84	2
LD (metabolite intensities under drought vs. loss of trait)						
Ethanesulfonic acid, 2-mercapto- (2TMS)	GrainLength	0.87	***	ns	0.61	1
Gulonic acid, 2-oxo-, DL- (1MEOX) (5TMS)	GrainLengthWidth	0.86	***	ns	0.48	4
Pregn-5-ene-3,21-diol-20-one (1MEOX) (2TMS)	GrainLengthWidth	0.85	***	ns	0.43	6
Maltose (1MEOX) (8TMS) MP	Biomass	−0.85	***	ns	0.7	2

Metabolic and imaging trait data from five control-treated and seven drought-treated plants per genotype 22 days after onset of drought (DAD)

^a^Partial least square regression

Significance levels: * *p* < 0.05; ** *p* < 0.01; *** p < 0.001; **** *p* < 0.0001; ***** *p* < 0.00001

Although no clear enrichment of chemical metabolite classes was observed, all six organic acids and three sugars/derivatives in the DD dataset showed positive associations with their respective phenotypes. Most metabolites linked to traits under drought conditions were positively correlated, while metabolites associated with loss of trait under control conditions were negatively correlated, suggesting that higher intensities of the analysed metabolites are generally linked to improved performance under drought.

One of the strongest associations was found between alpha-D-glucopyranosyl-(1,6)-D-mannitol and SpikeLength in the CC data (Spearman’s ρ = −0.91, FDR-adjusted *p*-value < 0.00001, *R*^2^ = 0.7). This metabolite was also the best predictor for SpikeLength based on its regression coefficient. Under drought, 2-amino-2-deoxygluconic acid showed a strong positive correlation with GrainArea (Spearman’s ρ = −0.90, FDR-adjusted *p*-value < 0.00001, *R*^2^ = 0.81) and was a very good predictor of this trait. Raffinose was strongly correlated with AwnLength under drought (Spearman's ρ = −0.90, FDR-adjusted *p*-value < 0.00001, *R*^2^ = 0.54). While its predictive ability for AwnLength was rather poor (rank 17), it was the strongest predictor for GrainNumber under control conditions (rank 1, Spearman’s ρ = −0.88, FDR-adjusted *p*-value = 0.03, *R*^2^ = 0.78). Other relevant associations for grain traits included highly significant correlations between GrainLength and the organic acids shikimic acid, orotic acid, and quinic acid (Spearman’s ρ = 0.87, FDR-adjusted *p*-value = 0.3, *R*^2^ = 0.57–0.59) under drought, as well as between loss of GrainArea and xylose and D-xylose (LC, Spearman’s ρ = −0.87, FDR-adjusted *p*-value = 0.04, *R*^2^ = 0.82–0.86).

## Discussion

### Early-season drought strongly affects yield-relevant traits

A key metabolic pathway affected by drought is photosynthesis. Stomatal closure, a typical drought response, reduces CO₂ availability, exposing photosystems to ROS, which compromises cellular integrity and photosynthetic efficiency. However, PSII efficiency and adaptability remained unaffected by drought in our study, aligning with Lauterberg et al. [48], who observed only a minor reduction in QY-Lss1 and QY-Lss2 and a slight increase in their ratio, in emmer wheat under drought (20% PAW). Similarly, Živčák et al. [49] found stable maximal PSII quantum efficiency in drought-stressed wheat until ~ 70% relative water content. These findings suggest PSII efficiency in wheat is largely maintained under the drought conditions in this and similar studies. In a study at the same facility, chickpeas showed reduced QY-Lss1 after 27 DAS [31]. In that study, the initial PAW was 70% and therefore, a critical drought stress was reached earlier, resulting in a more pronounced effect on PSII efficiency. Similarly, an effect on barley was identified under the same conditions (K. Neumann, personal communication), which may be explained by the higher biomass of barley at this stage compared to wheat, which made barley more susceptible to drought stress.

Despite the apparent resilience of PSII, drought severely impacted key yield-related traits directly or indirectly linked to photosynthesis, including TillerNumberGain, hsv_h_brown2green, PlantWeight and TGW. A higher brown-to-green ratio suggests a greater proportion of senescing leaf biomass, further reducing the photosynthetically active area. The treatment effect was less pronounced in hsv_h_red2green and hsv_h_yellow2green, suggesting that drought primarily induced senescence rather than carotenoid or anthocyanin production, which protects photosystems from oxidative damage [50, 51]. Lower correlations and heritability in some imaging-based traits are consistent with previous reports using the same system and may reflect individual differences in maturation or senescence [31]. The hsv_h_red2green ratio is usually very low, making the number of red pixels in the image more sensitive to outliers, which reduces heritability. Additional noise is introduced by the plants being moved and photographed from a different side every day, and thus, an uneven distribution of red plant parts may influence the results. Fluctuations in width measurements during the late drought stage may be influenced by changes in leaf angles induced by wilting or the time of day when the imaging took place, which conceivably have a larger effect on longer leaf sheaths present in older plants than on shorter leaf sheaths in younger plants. Additionally, watering and imaging times were randomized each day, and therefore, the time between watering and imaging may have introduced small daily fluctuations in water status of the plants. Overall, the high heritability and correlations across experiments confirm the suitability of HTP facilities for minimizing environmental effects in –omics studies.

### Metabolic changes during drought

Differences in sample size between sampling time points and treatments at 22 and 28 DAD may have affected statistical power for DAM detection at these time points. However, an FDR < 0.05 and the log_2_(FC) cutoff should ensure that false positives were reduced to a minimum.

At 7 DAD, most traits did not show significant drought-induced effects, and PCA plots did not reveal clustering by treatment, suggesting low stress intensity despite an average PAW of 48.4% in stressed plants. Accumulation of RFOs accumulation, an early drought response, was minimal, with raffinose showing only a slight increase at 7 DAD and low but significant accumulation at 13 DAD, possibly because accumulation had already occurred prior to M1 or because stress was not yet severe enough to trigger raffinose accumulation. Similarly, proline, another early drought marker, did not substantially accumulate until after 13 DAD, indicating moderate stress intensity at 7 and 13 DAD, which is supported by the lack of treatment effect in the PCA plots.

However, some metabolites were differentially accumulated at this early stage. Six were upregulated, and eight were downregulated under early drought, with two (3-methyl-pentanedioic acid and 2-phenylacetamide) showing a log_2_(FC) > 1. Glutaric acid, a derivative of 3-methyl-pentanedioic acid (not evaluated in this study), accumulated in drought-tolerant rice and in sesame undergoing drought stress [52, 53].

Among the DAMs at 7 and 13 DAD were putrescine and spermine, two polyamines involved in drought responses. Putrescine, derived from arginine or ornithine, is a precursor for both spermidine and spermine [54]. These three polyamines play a role in many fundamental processes, but also in stress-related functions such as osmolyte balance, stomatal regulation and free radical scavenging to protect thylakoid membranes from oxidative damage, thus helping in maintaining photosynthetic activity and chlorophyll function [54–56]. Exogenous spermine and putrescine applications can alleviate drought effects on plant development, improve photosynthetic activity and water use efficiency, and increase the production of other stress-related compounds such as proline, soluble sugars, anthocyanins, and phytohormones such as ABA [57, 58].

Putrescine levels decreased under drought across all time points, while spermine significantly accumulated at 7 and 13 DAD. Putrescine decreased until 22 DAD under drought, with no clear pattern in controls. In *Rosa damascena*, putrescine accumulated to high levels after one day of drought (50 or 25% field capacity), and at six days was only slightly increased compared to the control at 100% field capacity [59]. In wheat, putrescine significantly accumulated in drought-tolerant and especially in drought-non tolerant genotypes between two and 12 days after polyethylene glycol (PEG)-induced drought, while spermine and spermidine increased, especially in the tolerant genotype [60]. This may indicate that putrescine is an early accumulating polyamine. Putrescine levels declined more strongly in tolerant lines (PPPW_017 and PPPW_033) from 7 to 22 DAD, while spermine levels peaked earlier in tolerant lines (13 vs. 22 DAD), suggesting that the timing of the conversion of putrescine to spermine and spermidine may be key for early drought responses. Indeed, spermine appears to be one of the most effective polyamines for alleviating drought stress, while spermidine synthesis is a process that is upregulated in several cereals and Arabidopsis under drought [57, 61]. Notably, the tolerant genotypes did not show higher overall polyamine intensities or greater accumulation under drought than non-tolerant genotypes, indicating that tolerance may be linked to metabolite dynamics rather than absolute concentrations. Further studies on a larger sample size and more time points are required to validate these observations.

At 13 DAD, several other known drought markers accumulated. Notably, nicotinamide accumulated significantly under drought relative to control at all time points except for 7 DAD and strongly increased under drought and decreased after rewatering. Interestingly, controls exhibited a similar pattern but at lower intensities. As a biostimulant, nicotinamide promotes plant growth either alone or in combination with phytohormones like auxin, cytokinin or gibberellin [62]. As a component of the coenzyme nicotinamide adenine dinucleotide (NAD), it is crucial for signal transduction, metabolism, ATP production, redox homoeostasis, signalling and ROS scavenging [63]. Its role in drought response remains underexplored, although it has been associated with increased indole-3-acetic acid, gibberellic acid and cytokinins and decreased ABA levels [64]. In *Arabidopsis thaliana*, the overexpression of nicotinamidase 3, the enzyme which deaminates nicotinamide to nicotinic acid, as well as the exogenic application of nicotinic acid, led to an increase of biomass under drought [65]. Exogenous nicotinic acid application alleviated the negative effect of drought on plant performance in wheat and barley and even improved plant growth compared to well-watered conditions [57, 64].

Notable changes in amino acid peak intensities were apparent, a well-known response to drought [18, 66]. Glutamine, asparagine, 5–6-dihydrothymine, selenomethionine, alanine and cysteine significantly decreased under drought whereas leucine, isoleucine, histidine and tyrosine accumulated slightly. Leucine and isoleucine typically increase during drought, which is in concordance with our findings [18, 67]. The influence of drought on alanine levels is not clear, with some studies reporting an accumulation [68, 69] and others a decrease under drought [70]. Alanine shows differential accumulation in tolerant and sensitive soybean genotypes [71], suggesting that its accumulation can be directly related to the tolerance level. Its accumulation seems to depend on the time and severity of drought, as it decreased under mild stress but accumulated under severe stress in rice [72]. Glutamine, the amino acid exhibiting the highest negative fold change at 13 DAD, is a precursor for proline, histidine and arginine, GABA, and chlorophyll and can affect stomatal regulation [73]. Cysteine, which influences ABA production, decreased, potentially indicating an impact on ABA and thus stomatal activity [74]. The BCAAs leucine and isoleucine commonly accumulate during drought [18]. Exogenous BCAA application can reduce drought-stress symptoms and improve water content and recovery in rice [75]. The mechanism by which they confer drought tolerance is not fully understood but it is thought that they act as osmolytes or energy sources [67].

Proline, a well-known drought stress marker [76, 77], accumulates later than RFOs and sometimes only under severe drought [72]. Consistent with this, it did not accumulate substantially before 22 DAD in this study. Of the drought markers shown in Additional file 14, it had the highest initial intensities and showed the strongest drought-induced accumulation, peaking at 22 DAD and decreasing quickly at the rewatering stage to intensities similar to 13 DAD. Proline, a cyclic amino acid produced from ornithine and glutamate, plays a role in redox balance, signalling, cell homeostasis, osmotic adjustment, and stabilization of proteins and membranes under drought and other stresses [16, 19, 20]. Its accumulation is generally linked to drought tolerance. Some studies report differential accumulation between drought-tolerant and non-tolerant wheat genotypes, while others did not observe a difference, suggesting that absolute proline concentrations do not necessarily correlate with drought tolerance [78–80]. Instead, differences in synthesis rate and utilization might play a role, possibly in an ABA- and calcium-dependent manner [78]. After the end of drought, it usually decreases to levels similar to those in control plants, which is in concordance with our results [79].

In addition to several amino acid biosynthesis pathways, pathways related to DNA and RNA damage and repair were enriched as early as 13 DAD, highlighting the effect of drought-induced oxidative stress and ROS on nucleic acid integrity [81]. One product of pyrimidine oxidation is 5–6-dihydrothymine, which surprisingly was reduced at the first three time points but increased during recovery. The enrichment of the basal transcription factors pathway suggests substantial drought-induced impacts on the transcriptome. Transcriptional and RNA regulation are commonly affected in by drought stress in several species [61].

Several metabolites consistently accumulated or decreased under drought across several time points. Trans-3,4,5-trimethoxycinnamic acid, which accumulates under drought and scavenges ROS [82], was elevated at all time points except 7 DAD and will be discussed later. Trans-aconitic acid, an isomer of the tricarboxylic acid (TCA) cycle intermediate cis-aconitic acid, increased at 7, 13, and 22 DAD. It is commonly found in high concentrations in grasses, and exogenous treatment of soybean led to decreased root growth and affected photosynthesis [83]. It also increased in durum wheat under drought, but its precise role in drought response remains unclear [84]. Similarly, the role of 2-mercaptoethanesulfonic acid, another metabolite that accumulated at all three drought time points, is not yet understood. Aminomalonic acid accumulated less under drought at 7, 13, and 22 DAD. It is involved in salt stress responses in hulless barley [85, 86] and upregulated in drought-tolerant rice. It is further accumulated in drought-stressed bentgrass treated with acibenzolar-S-methyl, a salicylic acid analogue which can improve heat and drought tolerance [87], suggesting that it may be involved in alleviating drought stress. Asparagine also accumulated less at 7, 13 and 22 DAD. Initially thought to play a similar role in stress responses as proline (ROS scavenging), it showed a negative correlation with yield in greenhouse studies and it was suggested to be involved in promoting senescence in response to drought [88, 89]. The reasons for its reduced accumulation under drought in our study remain unclear, but it is possible that senescence was not yet pronounced enough to induce asparagine accumulation.

Overall, these findings suggest that early drought responses involve polyamine metabolism, amino acid shifts, and oxidative stress management. The differential accumulation of key metabolites across multiple time points highlights potential biomarkers for drought stress, warranting further exploration.

### Metabolic processes during recovery

At 28 DAD, five days post-rewatering, the drought markers histamine, spermidine and putrescine were reduced in previously stressed plants. Interestingly, several amino acids, including glycine, tyramine, alanine, lysine, isoleucine, leucine, methionine, histamine, and cytosine, accumulated less under drought at 28 DAD, while tyrosine, glutamine, histidine, selenomethionine, asparagine, and 5,6-dithiothymine increased. Asparagine, selenomethionine, 5,6-dithiothymine, glutamine, cysteine, and arginine increased at 28 DAD compared to 22 DAD, while proline, glycine, lysine, ornithine, isoleucine, leucine, and tyrosine decreased. The reduction of osmolyte levels such as polyamines and BCAAs during recovery is not yet understood but suggests profound drought-induced metabolic reprogramming. Rice plants overaccumulating BCAAs recover faster after drought, suggesting BCAAs as potential breeding targets [75].

Key enriched pathways between 22 and 28 DAD included beta-alanine metabolism (all DAMs), glycosaminoglycan degradation, glycine/serine/threonine metabolism (DAMs accumulated at recovery) and galactose metabolism, and ABC transporters (DAMs reduced at recovery). The beta-alanine pathway, enriched by spermine and spermidine (precursors of beta-alanine), also involved histidine and malonic acid. Galactose, a precursor of galactinol, which is necessary for early RFO production [90], is needed less under well-watered conditions, as RFO accumulation is limited. Further, plants are very sensitive to high galactose concentrations, and thus, excess galactose needs to be converted into other sugars quickly. Accordingly, galactinol was strongly and significantly reduced after rewatering (28 DAD vs. 22 DAD) and in recovering plants at 28 DAD. Drought-induced effects on galactose metabolism are conserved across several plant species, such as cereals [61]. Expectedly, carbon metabolism and the Calvin cycle were also enriched in recovering plants. Carbohydrate metabolism was identified as a conserved drought response in a microarray meta-analysis across Arabidopsis, rice, wheat and barley [61]. The ABC transporter pathway was enriched by amino acids (alanine, lysine, proline, leucine), sugars (fructose, lactose), and nucleosides (adenosine, guanosine). ABC transporters are part of conserved responses to abiotic and biotic stress across species [91]. They are membrane transporters that have a wide range of substrates, e.g. hormones such as ABA, to regulate stomatal closure, or wax and cutin to reduce water loss. The overexpression of ABC genes is linked to drought tolerance in *Arabidopsis thaliana*, faba bean, chick pea and other species [92–94]. In wheat, the ABC transporter Cer5 is involved in wax transport which has been linked to improved drought tolerance, but generally, the role of ABC transporters in drought stress in wheat is underexplored [95]. The enrichment of ABC transporters in DAMs reduced at recovery might be explained by the reduced need to manage the water status in recovering plants.

### Superior performance under drought is related to oxidative and osmotic stress responses and energy metabolism

None of the twelve genotypes showed superior drought tolerance, despite some (PPPW_007, PPPW_012, PPPW_046) being selected as tolerant under field conditions during the 2018 drought. In the pre-screening, PPPW_011, PPPW_012, PPPW_017, PPPW_023, PPPW_025, and PPPW_046 were classified as tolerant, while in the time-course experiment, PPPW_017 and PPPW_033 were considered tolerant and PPPW_007 and PPPW_011 non-tolerant. This discrepancy likely stems from differing selection criteria. The pre-screening relied on flowering time, tiller number, biomass, and PAW, while the time-course experiment assessed yield-related traits.

Several factors can explain the differences between field and controlled greenhouse evaluations. First, different subsets of genotypes were tested at different locations in the Nordic countries, where tolerance was assessed as yield relative to location-specific standard cultivars, the performance of which influenced the classification. Second, drought stress in the field was likely compounded by heat stress. Additionally, other abiotic and biotic factors like soil properties and pests and diseases may have had an influence in the field but were controlled in the greenhouse. Finally, field drought conditions likely differed in severity and duration from that in the greenhouse experiment.

The DAMs accumulated in tolerant genotypes at 22 DAD suggest a role of oxidative and osmotic stress responses (trans-3,4,5-trimethoxycinnamic acid, cis-3-caffeoylquinic acid) and energy homoeostasis (adenosine-5-monophosphate). The cinnamic acid-derivative trans-3,4,5-trimethoxycinnamic acid, a phenolic acid produced in the phenylpropanoid pathway, is decreased in salt stressed rice and after grapevine fabavirus infection of grapevine leaves, but its role in drought responses is not well-studied [96–98]. In cucumber leaves, exogenous application of cinnamic acid alleviates PEG-induced drought by increasing the activity of ROS-scavenging enzymes such as superoxide dismutase and ascorbate peroxidase, and by accumulating glutathione, ascorbate, proline, and soluble sugars [99]. Cinnamic acid was also reduced under drought in a drought-sensitive wheat genotype after 15 days of drought [77]. Cis-3-caffeoylquinic acid (chlorogenic acid), another phenolic acid, increased in all four pairwise analyses of tolerant vs. non-tolerant genotypes. Its role in drought-tolerance is unclear, but quinic acid is highly accumulated in drought-tolerant cherry rootstocks [100]. Quinic and chlorogenic acid concentrations decrease under drought, especially in drought-sensitive genotypes [77, 101]. In addition to its roles in osmotic adjustment, quinic acid is a product of a branch of the shikimic acid pathway, which produces phenylalanine, tyrosine and tryptophan, precursors for secondary metabolites like flavonoids and alkaloids involved in abiotic stress responses [102, 103]. Both the shikimic acid and phenylpropanoid pathway are involved in lignin and flavonoid synthesis [104–106]. Lignin biosynthesis is usually increased under drought, and lignification can stabilize cells and reduce water loss by improving water transport in the plant and reducing transpiration [107, 108]. Flavonoids alleviate drought stress symptoms by removing and inhibiting ROS [109].

The deoxyribonucleoside 2*-deoxyguanosine is an integral component of DNA. During oxidative stress, ROS hydroxylate guanine, which results in 8-hydroxy-2’-deoxyguanosine. This metabolite can form bonds with both cytosine and adenine, which leads to mutations in the DNA sequence [110]. The higher intensity of 2*-deoxyguanosine in tolerant genotypes could reflect a more efficient response to oxidative stress, possibly resulting in less DNA damage.

### Metabolites correlated with yield component traits might be helpful for predicting phenotypic performance

This study identified 39 significant metabolite-trait correlations (*p* < 0.001, Spearman’s ρ < −0.8 or > 0.8), suggesting that certain metabolites could serve as biomarkers for drought tolerance screenings and for predicting plant performance under stress. Most metabolites were positively correlated with traits or negatively with percentage loss of trait, indicating that higher metabolite levels corresponded to better and more stable drought performance. The grain size traits (area, length, width, and length/width ratio) had the most associations (*n* = 15), highlighting their potential for metabolite-based screenings. The strongest association was between 2-amino-2-deoxygluconic acid and grain area under drought. Exogenous treatment with ammonium gluconate alleviates drought-induced reductions of fresh weight, chlorophyll content, and leaf area in rice, improving water uptake, root lignification, and number of root tips as well as reducing aerenchyma formation, suggesting that gluconate can reduce damage caused by drought through root morphology modifications [111].

It is easily conceivable that improved water availability in the plant leads to bigger grains even under drought. Low xylose intensities under control conditions were associated with a greater loss of grain area and width. Xylose is a component of the hemicellulose xylan, an integral part of plant cell walls. Arabidopsis mutants with reduced xylose and galactose contents exhibited decreased drought tolerance, while mutants deficient in xylose but enriched in arabinose in the cell wall displayed improved drought tolerance [112]. Arabidopsis genotypes with low xylan and lignin contents also showed good drought tolerance, possibly in an ABA-dependent manner [113]. The content of certain fibres can change during drought. For example, arabinoxylan increased while β-glucan decreased in wheat grains under drought and heat stress, but these effects can be dependent on genotype and drought severity [114, 115]. The osmoprotectant trehalose, positively correlated with GrainArea under drought in this study, improves drought tolerance, e.g. in rice and wheat [116, 117], by scavenging hydroxyl radicals, maintaining superoxide dismutase activity, and regulating stomatal conductance [16, 118]. The association of shikimic and quinic acid with grain length under drought suggests an involvement of the shikimic acid pathway and its products. During drought, shikimic acid pathway intermediates such as shikimic and quinic acid were decreased in maize roots [119], while higher levels may indicate increased drought tolerance and hence better performance. Shikimic acid was reduced in the sorghum *by-1* mutant, which is characterized by lower biomass, grain yield, and grain width compared to the wild type, suggesting a general involvement of the shikimic acid pathway in grain development [120]. Interestingly, putrescine was negatively correlated to hsv_h_brown2green ratio, suggesting that a higher intensity of putrescine allows plants to stay green for longer under drought, as it positively affects chlorophyll metabolism in heat-stressed tomatoes [121]. However, differences in accumulation between species have been observed for several metabolites such as trehalose or sucrose (reviewed in Fàbregas and Fernie 2019), therefore care must always be taken when drawing conclusions across species.

### Limitations of this study and future research directions

The limited size of the genotype panel in this study warrants further testing in larger panels and diverse environments to validate their predictive ability for drought tolerance. Studying drought responses of a larger panel under field conditions would be desirable, however, the potential presence of additional stresses (e.g. heat stress) might also make it difficult to identify drought-specific responses. A lower of number of replicates in field trials might lead to less sensitivity to environmental effects.

Many of the metabolite-trait correlations were not significant after FDR correction. However, the high Speaman’s correlations, *R*^2^ values and the generally very high PLS ranks support our interpretation that these metabolites are promising candidates for metabolite-based selection. Further work could include exogenous application of candidate biomarkers to test their effect on drought tolerance. In this study we identified nearly 200 metabolites that were differentially accumulated under drought or at the recovery stage, but the role of many of these metabolites and associated pathways in drought tolerance is not yet understood. The study of transcriptomic and proteomic response to drought would be a valuable addition to the current study and contribute to a more comprehensive understanding of spring wheat’s response to early-season drought stress. The integration of additional -omics data could also serve as a validation of our results. Gene-editing of differentially expressed genes or key genes in pathways important for drought responses will ultimately allow the functional validation of key metabolites and associated pathways.

## Conclusion

In conclusion, none of the 12 tested genotypes exhibited superior drought tolerance, making classification by tolerance levels challenging. The chosen approach emphasized performance and stability of several yield-relevant traits and should therefore be robust. These traits were among the most affected by drought, underlining the challenge this abiotic stress poses for wheat production. Almost 200 metabolites were identified as differentially accumulated at four different time points between early drought and the recovery phase, as well as between tolerant and susceptible genotypes. While amino acid and polyamine synthesis were pivotal responses, sugar accumulation appeared less influential, possibly due to the limited number of sugars identified. Some metabolites, such as trans-aconitic acid and asparagine, were consistently altered under drought, but their roles remain unclear.

Metabolites such as xylose, trehalose, and shikimic acid intermediates showed promising associations with drought tolerance, particularly related to grain development and stress mitigation. These findings suggest that certain metabolites could serve as biomarkers for drought tolerance. However, due to the limited genotype sample, further studies across larger panels and diverse environments are necessary to validate these results.

In conclusion, this study underscores the complexity of drought tolerance in wheat and demonstrates the potential of combining metabolomics with high-throughput phenotyping to assess plant stress responses. These approaches offer valuable insights for identifying biomarkers and improving drought resistance in crops.

## Supplementary Information

Additional file 1. List of genotypes used in this study. Shown are PPPW codes, breeding company origins, pilot study phenotypes (biomass after 22 of drought (DAD), days to flowering, plant available water at 22 DAD, tiller number gain), 2018 field drought tolerance, and drought tolerance from this study. Lines used for time-course studies are highlighted in yellow. Unreleased lines are coded.

Additional file 2. Description of imaging-based and post-harvest traits evaluated in this study. Shown are the sampling environment, time point and a description of the data.

Additional file 3. Phenotypic values for imaging-based traits collected in this study. Data were filtered as described in Materials and Methods. Traits are described in Additional file 2.

Additional file 4. Phenotypic values for post-harvest traits collected in this study. Data were filtered as described in Materials and Methods. Traits are described in Additional file 2.

Additional file 5. Examples of images taken at the time point of strongest drought stress (45 days after sowing, DAS) and at the last day of imaging (48 DAS).

Additional file 6. Peak intensities of metabolites. Data were filtered as described in Materials and Methods.

Additional file 7. Broad sense heritability (H^2^) over time for imaging-based traits across experiments, separately for each treatment. The x-axis shows the days after the onset of drought (DAD).

Additional file 8. Spearman’s ρ between both experiments over time for imaging-based traits across experiments, separately for each treatment. The x-axis shows the days after the onset of drought (DAD).

Additional file 9. Spearman’s ρ and broad sense heritability (H^2^) for traits evaluated after harvest across experiments, separately for each treatment.

Additional file 10. Biomass accumulation (in voxels) over time for each genotype. Grey areas show 95% confidence intervals, and dashed curves indicate percent PAW over the experiment. Red dashed vertical line marks the end of the drought phase. The x-axis shows the days after the onset of drought (DAD). The y-axis shows biomass in voxels.

Additional file 11. Analysis of variance (ANOVA) results for the effects of genotype, treatment, and their interaction on each phenotype. *p*-values are reported for each factor. Phenotypic means were calculated per genotype and day.

Additional file 12. Percent loss of trait per day for imaging-based traits. Imaging traits are described in Additional file 2.

Additional file 13. PSI measurements for each genotype and treatment at four time points (14, 21, 23, and 27. The x-axis shows the days after the onset of drought, DAD): a) Quantum yield at high light intensity (800 μm/m²/s), b) Quantum yield at low light intensity (80 μm/m²/s), c) Ratio of the two quantum yields.

Additional file 14. Row-normalized peak intensities of known drought-responsive metabolites at the four sampling time points (7, 13, 22, and 28). The x-axis shows the days after the onset of drought, DAD) for each genotype and treatment.

Additional file 15. T-distributed stochastic neighbor embedding (t-SNE) analysis of metabolic profiles by individual plant and treatment. a) 7 days after the onset of drought (DAD), b) 13 DAD, c) 22 DAD and d) 28 DAD. Colours indicate biological replicates per genotype, shapes indicate treatment.

Additional file 16. Differentially accumulated metabolites (DAMs) identified at the different time points (7, 12, 22, and 28 days after the onset of drought, DAD), and between 22 and 28 DAD (rewatering). Shown are the log_2_(fold change), the mean normalized intensity under drought and under control, the t-test statistic, *p*-value and FDR-adjusted *p*-value.

Additional file 17. Metabolic responses to drought at 13 days after the onset of drought (DAD). a) Volcano plot showing differentially accumulated metabolites (DAMs) accumulated (red) reduced (blue) under drought compared to control treatment (T-test, FDR-adjusted *p*< 0.05, -1 < fold change < 1). b) Log_2_-transformed, row-normalized intensities of metabolites.

Additional file 18. Biplots placing the genotypes based on their metabolic profile at different time points (7, 13, 22, and 28 days after the onset of drought, DAD) under different conditions. Arrows represent metabolites, with length and direction indicating their impact.

Additional file 19. Differentially accumulated metabolites in tolerant and non-tolerant genotypes. Panels a and b: Metabolites differentially accumulated in tolerant (PPPW_017 and PPPW_033) and non-tolerant (PPPW_007 and PPPW_011) genotypes under drought compared to control conditions at 22 days after the onset of drought (DAD). Upset plots show the distribution of metabolites increased (a) and reduced (b) under drought in tolerant and non-tolerant genotypes. Panels c and d: Metabolites differentially accumulated between tolerant and non-tolerant genotypes under drought at 22 DAD. Upset plots show the number of metabolites increased (c) and reduced (d) under drought in tolerant vs. non-tolerant pairwise comparisons.

Additional file 20. Differentially accumulated metabolites in pairwise comparisons between tolerant and non-tolerant genotypes. Volcano plot showing differentially accumulated metabolites (DAMs) accumulated (red) reduced (blue) in the tolerant genotype (T-test, FDR-adjusted p < 0.05, -1 < fold change< 1). A) PPPW_003 vs. PPPW_011, b) PPPW_017 vs. PPPW_011, c) PPPW_033 va.PPPW_007, d) PPPW_017 vs. PPPW_007.

Additional file 21. Log_2_-transformed, row-normalized intensities of metabolites identified in Fig. 11a (differentially accumulated metabolites between 22 days after the onset of drought (DAD) and 28 DAD, after rewatering).

## Data Availability

All data generated or analysed during this study are included in this published article (Additional files 3-5). The code used for the analyses can be found at https://github.com/rwonneberger/Wonneberger_2025_Drought_stress_metabolomics_wheat (will be made public upon publication of the manuscript)
