# Supplementary material for: Integrating metabolomics and high-throughput phenotyping to elucidate metabolic and phenotypic responses to early-season drought stress in Nordic spring wheat
Source: BMC Plant Biol. 2025 Jul 30;25:987. doi: 10.1186/s12870-025-06914-y (PMC12309201; doi:10.1186/s12870-025-06914-y)
Supplement: Supplementary file 2 — Additional file 2. Description of imaging-based and post-harvest traits evaluated in this study. Shown are the sampling environment, time point and a description of the data. [file 12870_2025_6914_MOESM2_ESM.docx]

**Integrating metabolomics and high-throughput phenotyping to elucidate metabolic and phenotypic responses to early-season drought stress in Nordic spring wheat**

Ronja Wonneberger^1^, John Charles D’Auria^2^, Kerstin Neumann^2^, Pernille Bjarup Hansen^3^, Jon Arne Dieseth^4^, Linda Kærgaard Nielsen^5^, Tarja Niemelä^6^, Firuz Odilbekov^7^, Fluturë Novakazi^1^^, Therése Bengtsson^1*^ and the CResWheat Consortium

^1^ Swedish University of Agricultural Sciences, PO Box 190, 23422 Lomma, Sweden

^2^ Leibniz Institute of Plant Genetics and Crop Plant Research, [Corrensstraße 3, 06466 Gatersleben, Germany](https://www.google.com/maps/place/data=!4m2!3m1!1s0x47a59603fea3a5a1:0xa2fead3b1aa5904?sa=X&ved=1t:8290&ictx=111)

^3^ Nordic Seed A/S, Kornmarken 1, 8464 Galten, Denmark

^4^ Graminor AS, Hommelstadvegen 60, 2322 Ridabu, Norway

^5^ Sejet Plant Breeding, Nørremarksvej 67, 8700 Horsens, Denmark

^6^ Boreal Plant Breeding Ltd, Myllytie 10, 31600 Jokioinen, Finland

^7^ Lantmännen ek. för., Udda Lundqvists väg 11, 268 31 Svalöv, Sweden

^^^ Current address: University of Rostock, Satower Str. 48, 18059 Rostock, Germany

* Corresponding author

Additional File 2

| **Traits** | **Environment** | **Time** | **Description** |
| --- | --- | --- | --- |
| Biomass | APPP-B facility | 21-49 DAS* | Digital biomass, zoom-corrected geometry trait based on visible-light combined view [voxel] |
| hsv_h_brown2green | APPP-B facility | 21-49 DAS | Color-related trait based on visible light side view |
| hsv_h_red2green | APPP-B facility | 21-49 DAS | Color-related trait based on visible light side view |
| hsv_h_yellow2green | APPP-B facility | 21-49 DAS | Color-related trait based on visible light side view |
| hsv_h_mean | APPP-B facility | 21-49 DAS | Color-related trait based on fluorescence side view |
| lab_a_mean | APPP-B facility | 21-49 DAS | Color-related trait based on visible light side view |
| lab_b_mean | APPP-B facility | 21-49 DAS | Color-related trait based on visible light side view |
| lab_l_mean | APPP-B facility | 21-49 DAS | Color-related trait based on visible light side view |
| Width | APPP-B facility | 21-49 DAS | Zoom-corrected geometry trait based on fluorescence side view [mm] |
| Height | APPP-B facility | 21-49 DAS | Zoom-corrected geometry trait based on visible-light side view [mm] |
| compactness | APPP-B facility | 21-49 DAS | geometry trait based on visible-light side view |
| PH_TopSpike | Greenhouse | At maturity | Plant height to the top of the spike [cm] |
| PH_BaseSpike | Greenhouse | At maturity | Plant height to the base of the spike [cm] |
| PH_FlagLeaf | Greenhouse | At maturity | Plant height to the flag leaf sheath [cm] |
| PH_TopNode | Greenhouse | At maturity | Plant height to the topmost node [cm] |
| SpikeCulmRatio | Greenhouse | At maturity | Ratio of spike length and culm length |
| SpikeNumber | Greenhouse | At maturity | Number of spikes per plant |
| PlantWeight | Greenhouse | At maturity | Plant fresh weight (aboveground) [g] |
| SpikeWeight | Greenhouse | At maturity | Spike fresh weight [g] |
| SpikeLength | Greenhouse | At maturity | Length of spike [cm] |
| AwnLength | Greenhouse | At maturity | Length of awns [cm] |
| NumberFertileSpikes | Greenhouse | At maturity | Number of fertile spikes per plant |
| InfertileSpikes | Greenhouse | At maturity | Number of fertile spikes per plant |
| GrainNumber | Marvin Seed Analyzer | At maturity | Number of grains per plant |
| GrainWeight | Marvin Seed Analyzer | At maturity | Weight of all grains per plant [g] |
| TGW | Marvin Seed Analyzer | At maturity | Thousand grain weight calculated from the grain number and grain weight per plant [g] |
| GrainDensity | Marvin Seed Analyzer | At maturity | Grain Density evaluated by Marvin Seed Analyzer |
| GrainArea | Marvin Seed Analyzer | At maturity | Area of all grains per plant in mm^2^ |
| GrainWidth | Marvin Seed Analyzer | At maturity | Width of all grains per plant in mm |
| GrainLength | Marvin Seed Analyzer | At maturity | Length of all grains per plant in mm |
| GrainLengthWidth | Marvin Seed Analyzer | At maturity | Ratio of length and width of all grains per plant as a measure of grain roundness |
| SpikeDensity | Greenhouse | At maturity | Rachis node number divided by spike length |
| RachisNodeNumber | Greenhouse | At maturity | Rachis node number |
| FertileGrains | Greenhouse | At maturity | Number of fertile grains per plant |
| InfertileGrains | Greenhouse | At maturity | Number of infertile grains per plant |
| TillerNumber1 | APPP-B platform | 31 DAS | Tiller number per plant counted at 31 DAS |
| TillerNumber2 | APPP-B platform | 45 DAS | Tiller number per plant counted at 45 DAS |
| TillerNumberGain | APPP-B platform |  | Difference between tiller number at 45 DAS and tiller number at 31 DAS |

*DAS: Days after sowing
