# Supplementary material for: Integrating metabolomics and high-throughput phenotyping to elucidate metabolic and phenotypic responses to early-season drought stress in Nordic spring wheat
Source: BMC Plant Biol. 2025 Jul 30;25:987. doi: 10.1186/s12870-025-06914-y (PMC12309201; doi:10.1186/s12870-025-06914-y)

Integrating metabolomics and high-throughput phenotyping to elucidate metabolic and phenotypic responses to early-season drought stress in Nordic spring wheat

Ronja Wonneberger, John Charles D'Auria, Kerstin Neumann, Pernille Bjarup Hansen, Jon Arne Dieseth, Linda Kærgaard Nielsen, Tarja Niemelä, Firuz Odilbekov, Fluturë Novakazi, Therése Bengtsson and the CResWheat Consortium

Additional File 5 Examples of images taken at the time point of strongest drought stress (45 days after sowing, DAS) and at the last day of imaging (48 DAS)

PPPW_003
Control

PPPW_003
Drought

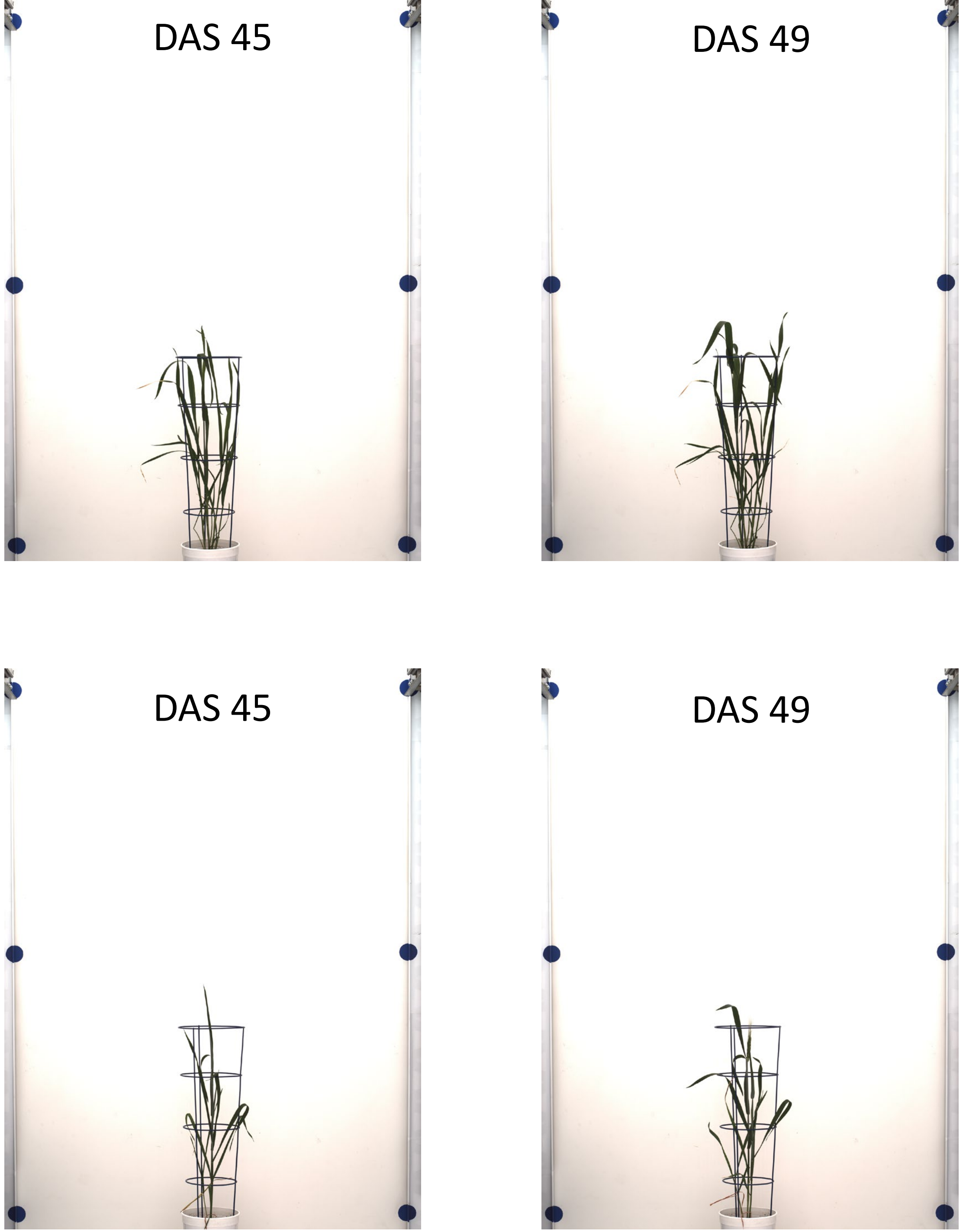

PPPW_004

Control

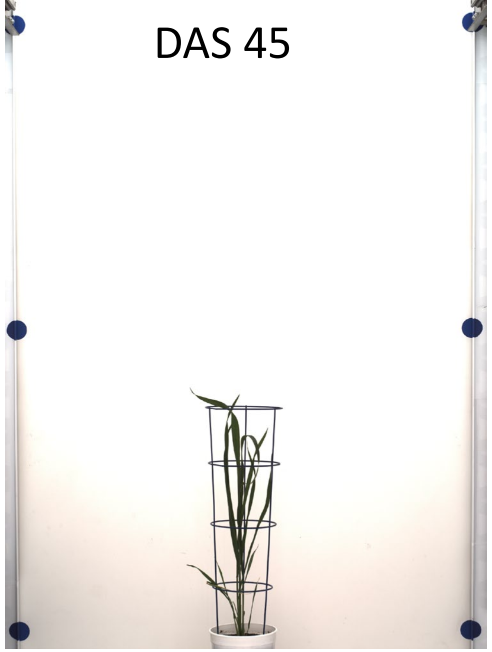

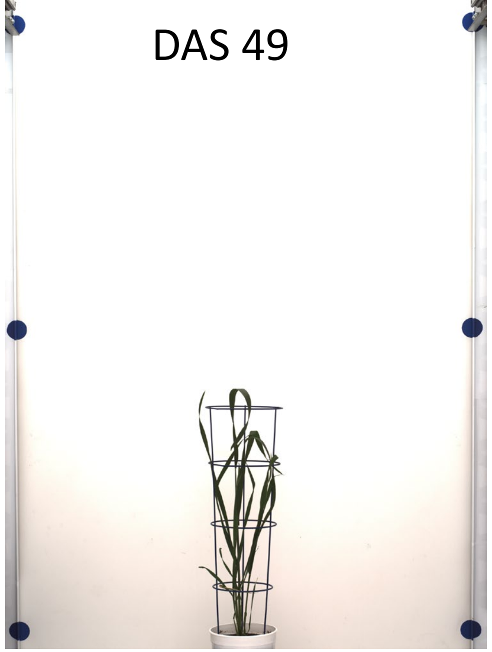

PPPW_004

Drought

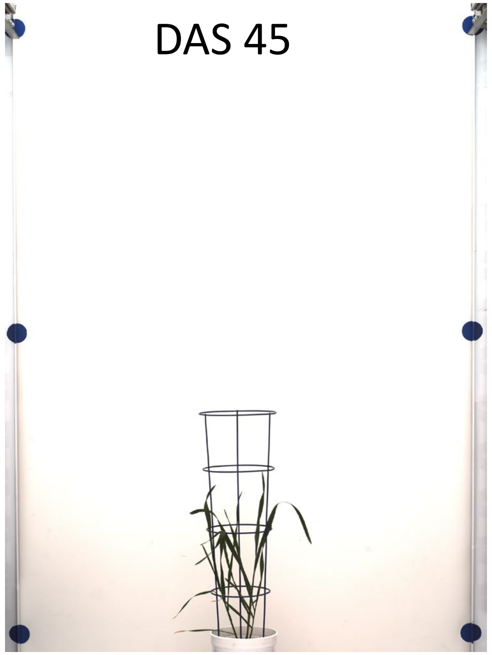

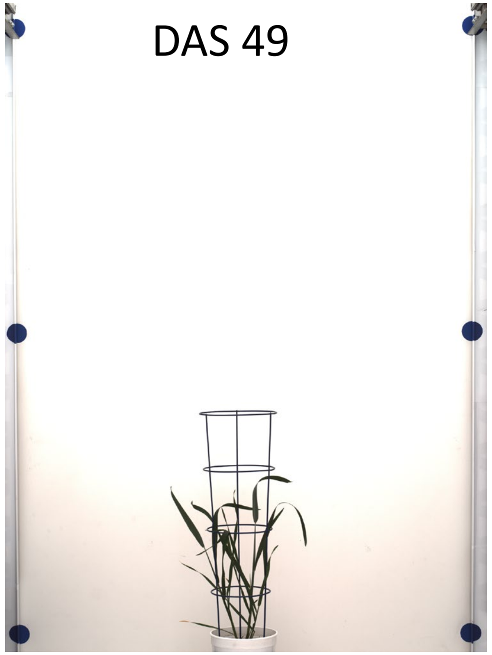

PPPW_007

Control

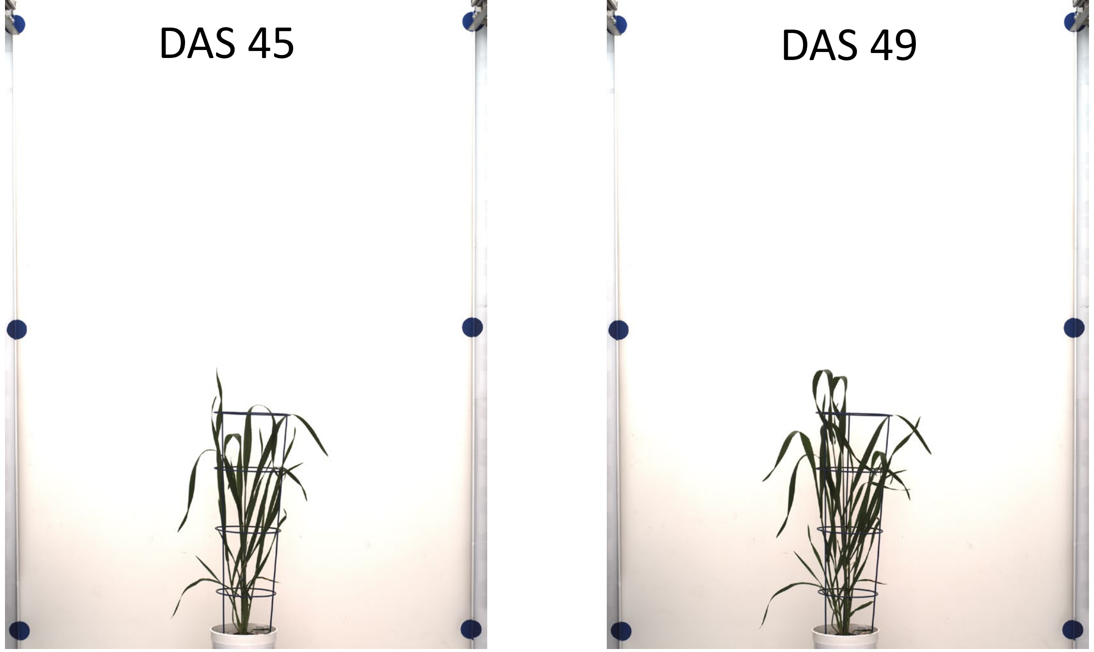

PPPW_007

Drought

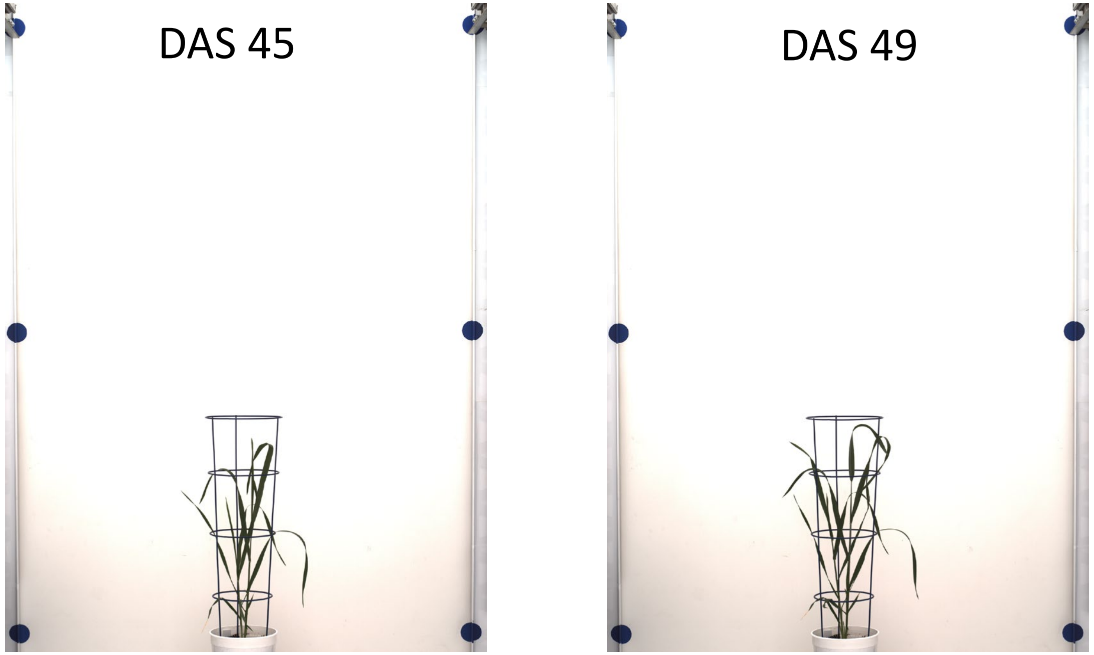

PPPW_011

Control

PPPW_011

Drought

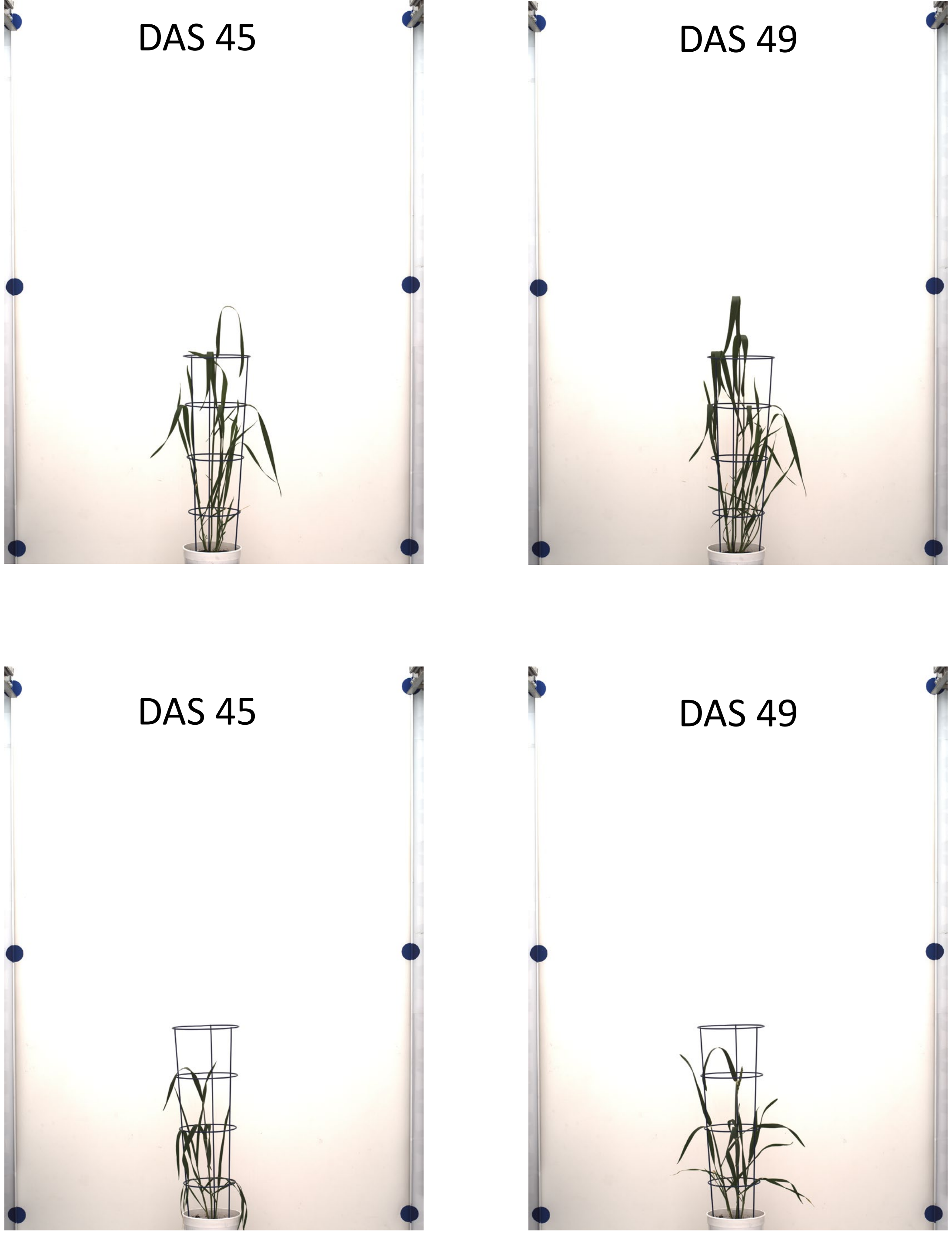

PPPW_012

Control

PPPW_012

Drought

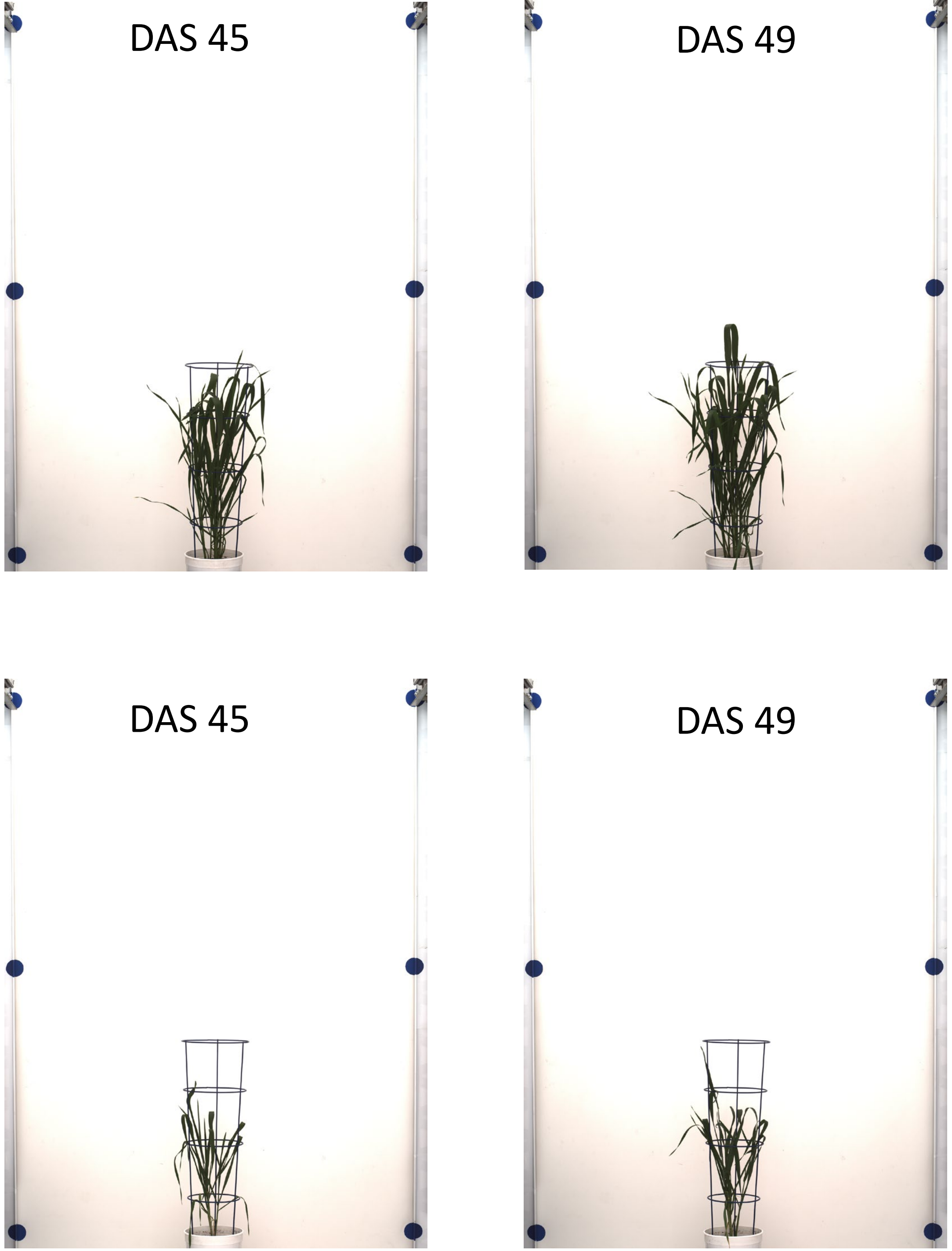

PPPW_013

Control

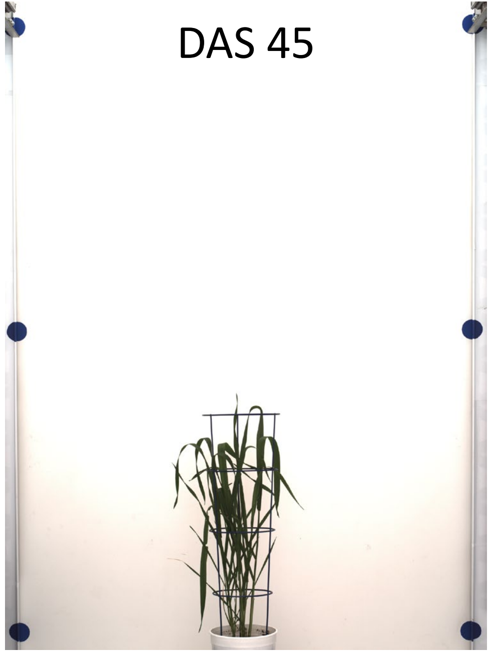

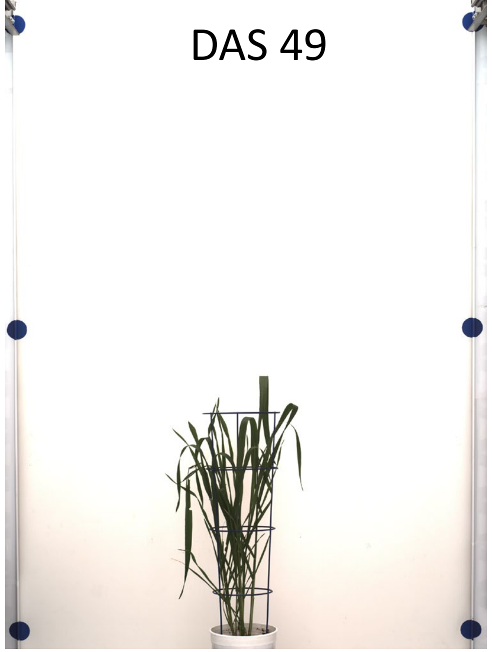

PPPW_013

Drought

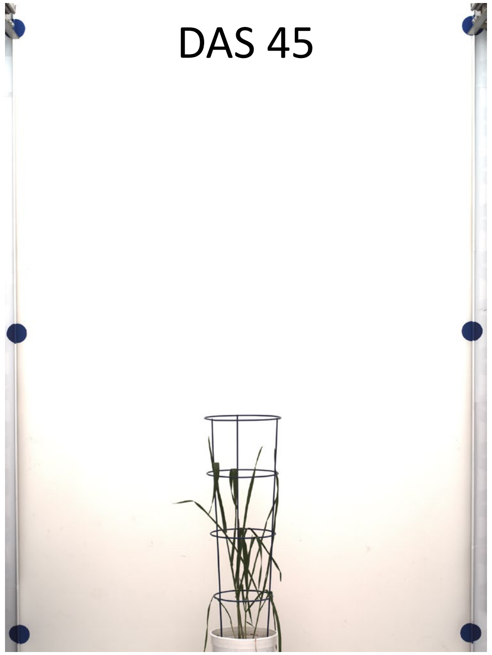

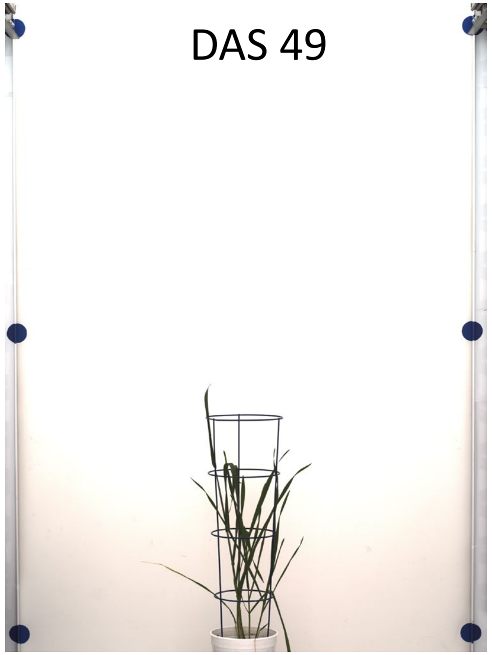

PPPW_017

Control

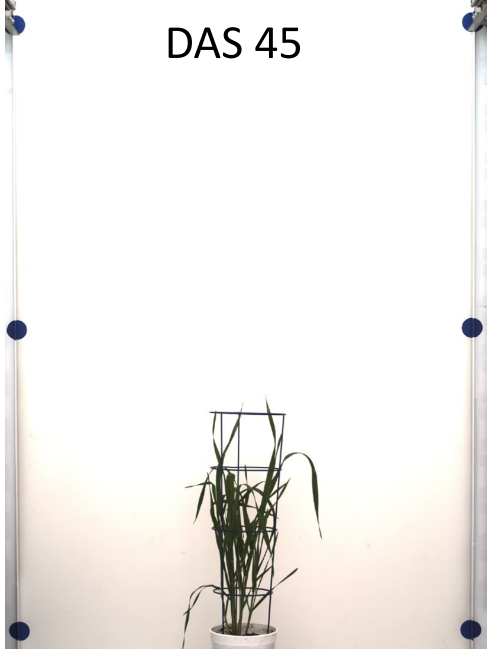

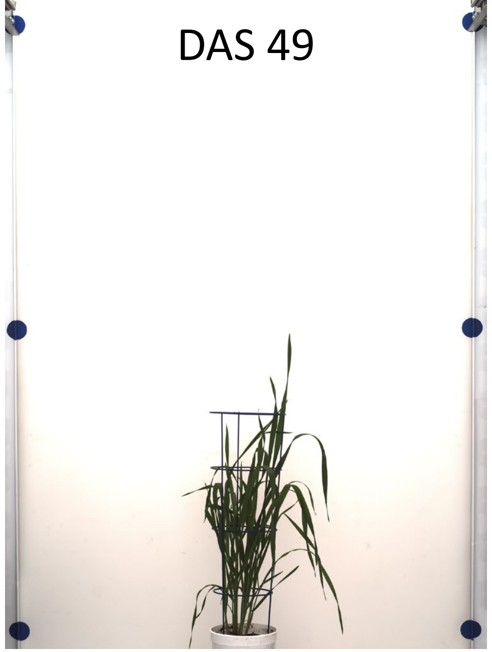

PPPW_017

Drought

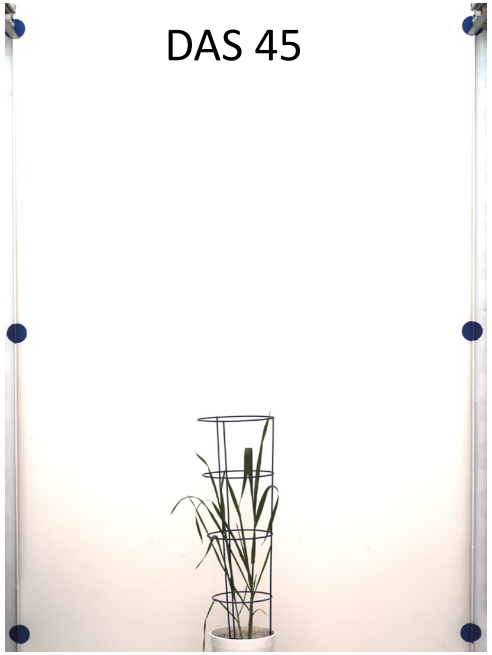

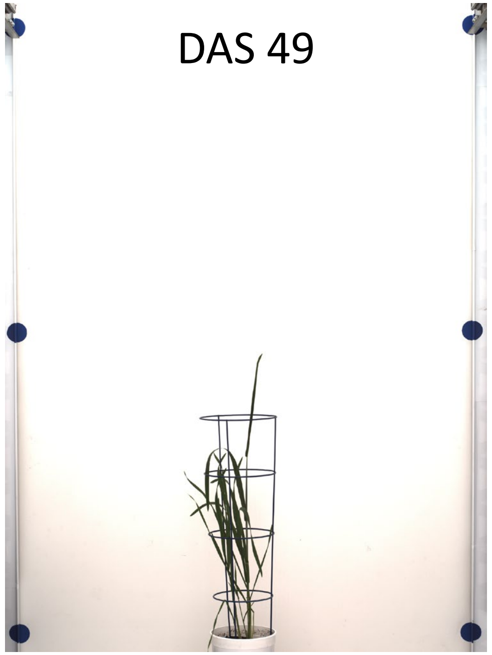

PPPW_023

Control

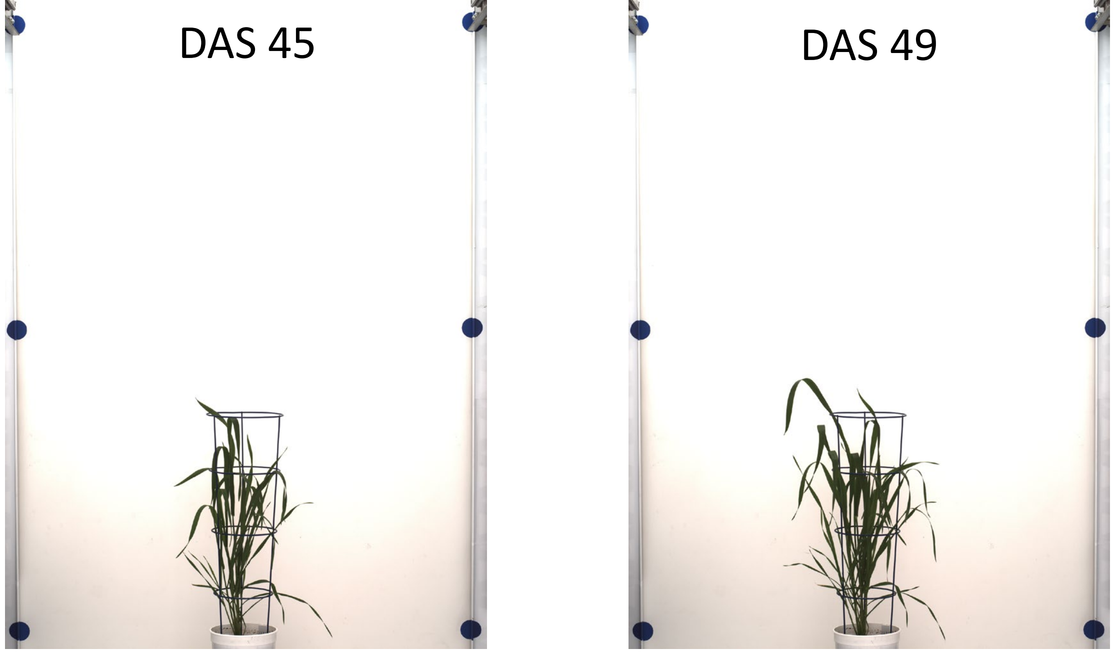

PPPW_023

Drought

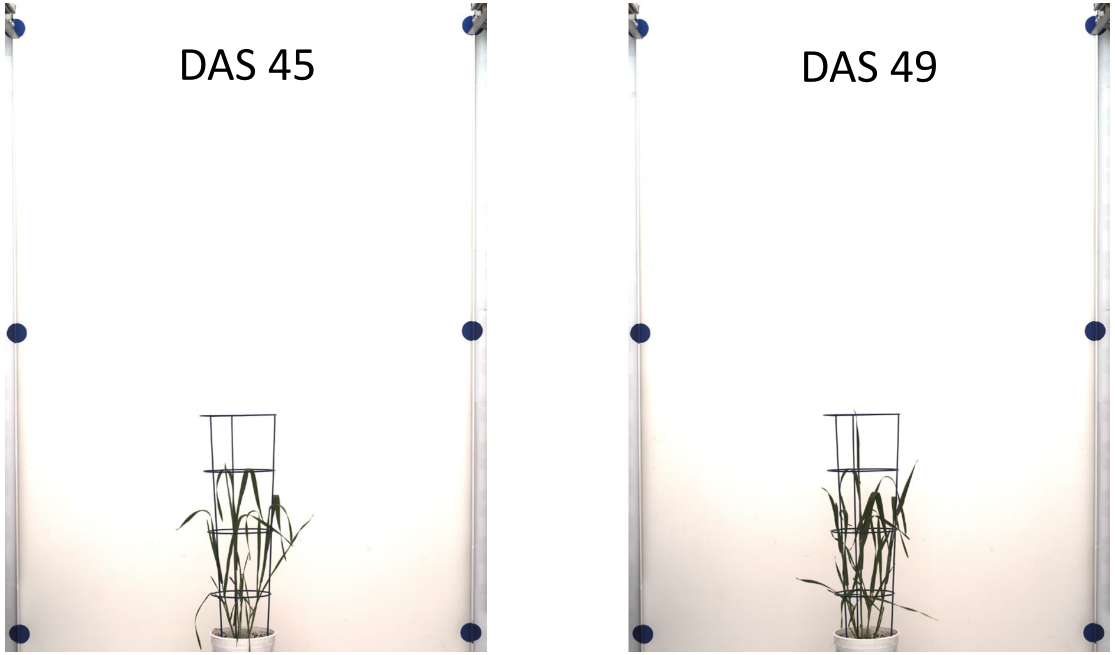

PPPW_025

Control

PPPW_025

Drought

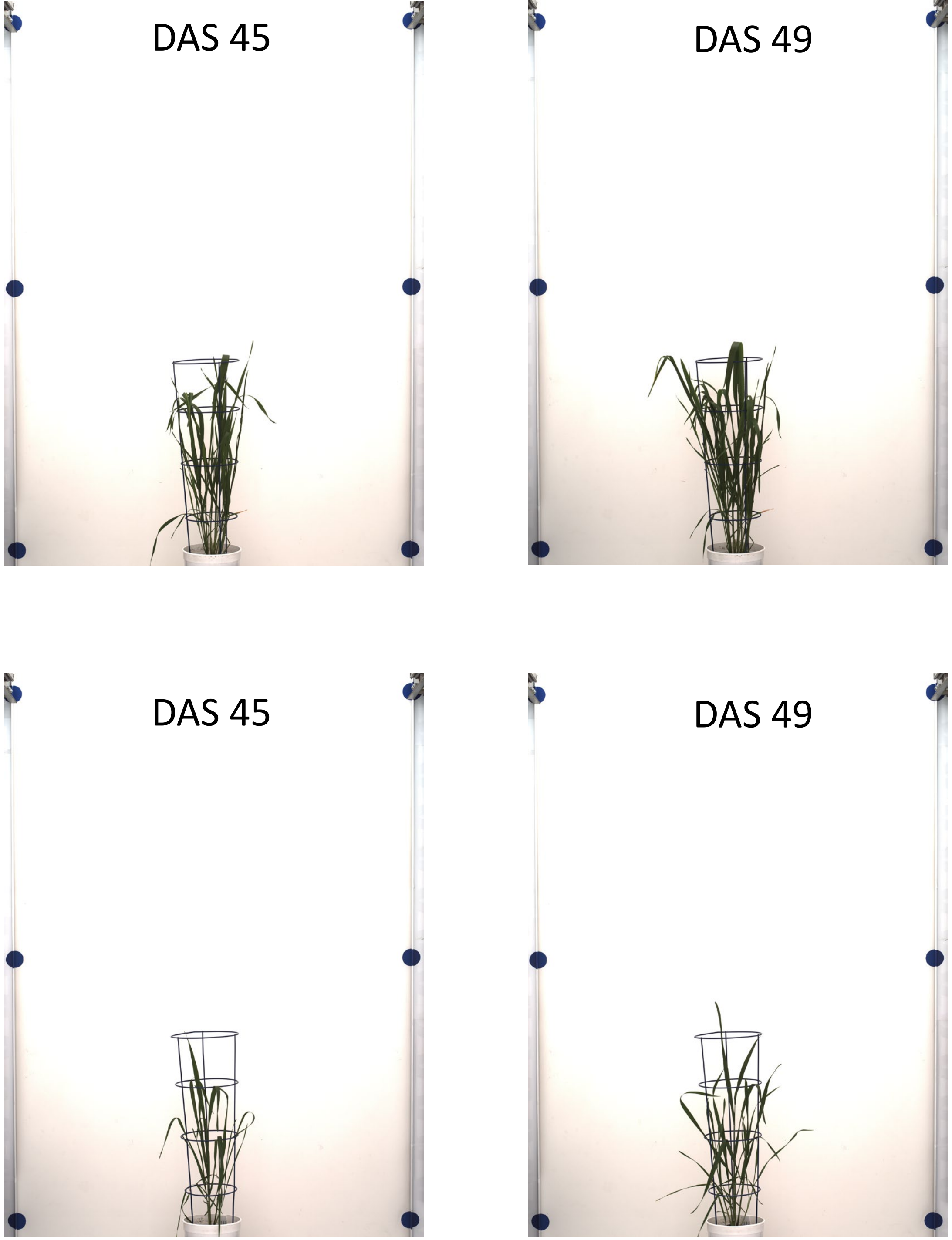

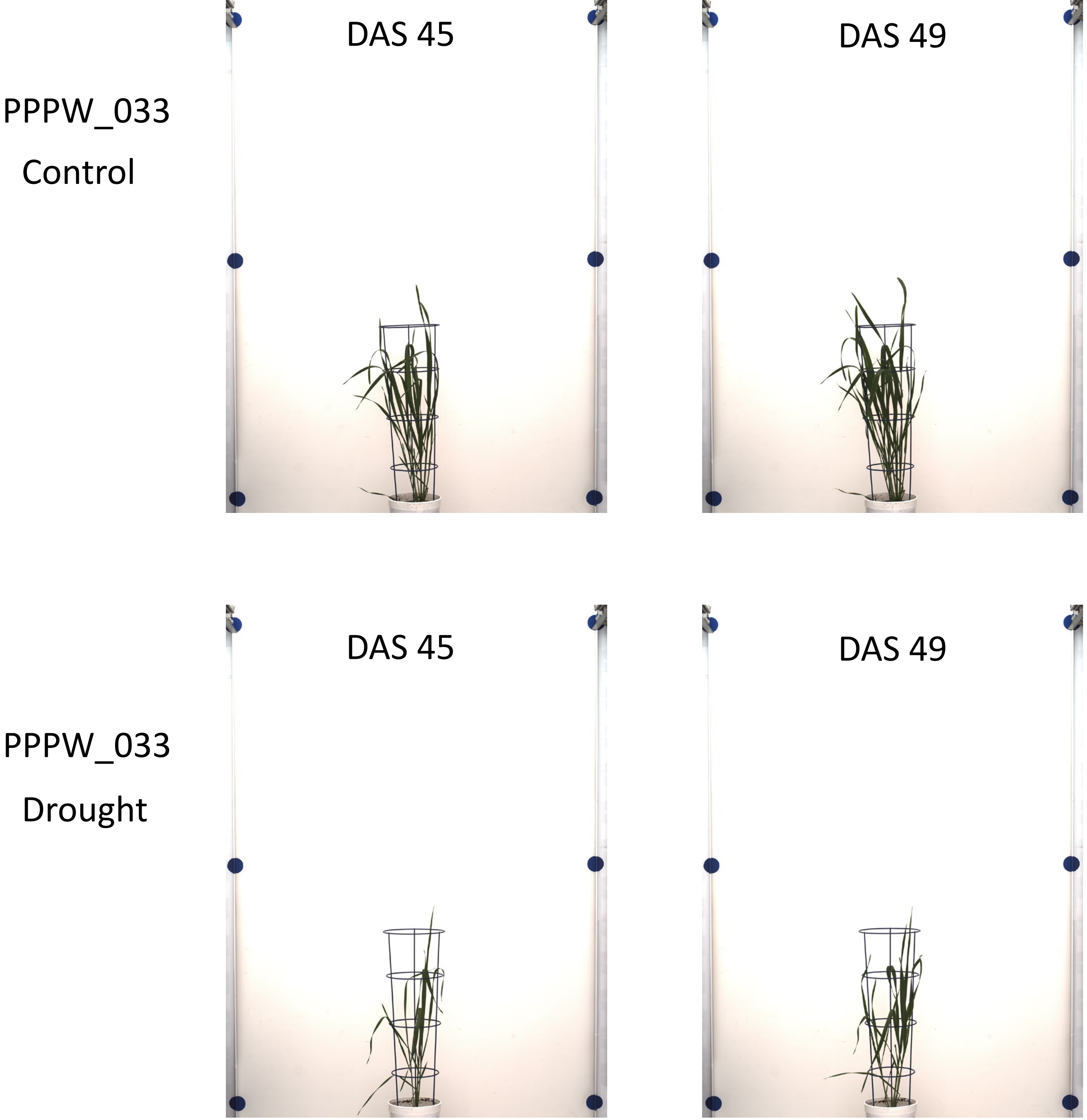
PPPW_033
Control
DAS 45
DAS 49
PPPW_033
Drought
DAS 45
DAS 49

PPPW_034
Control

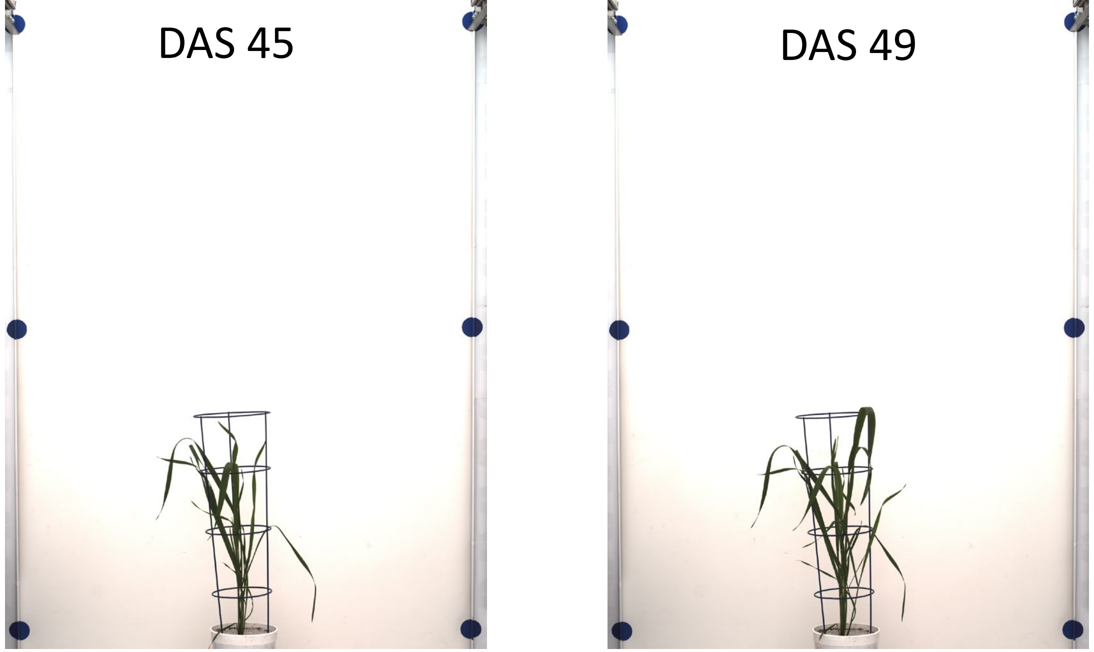

PPPW_034
Drought

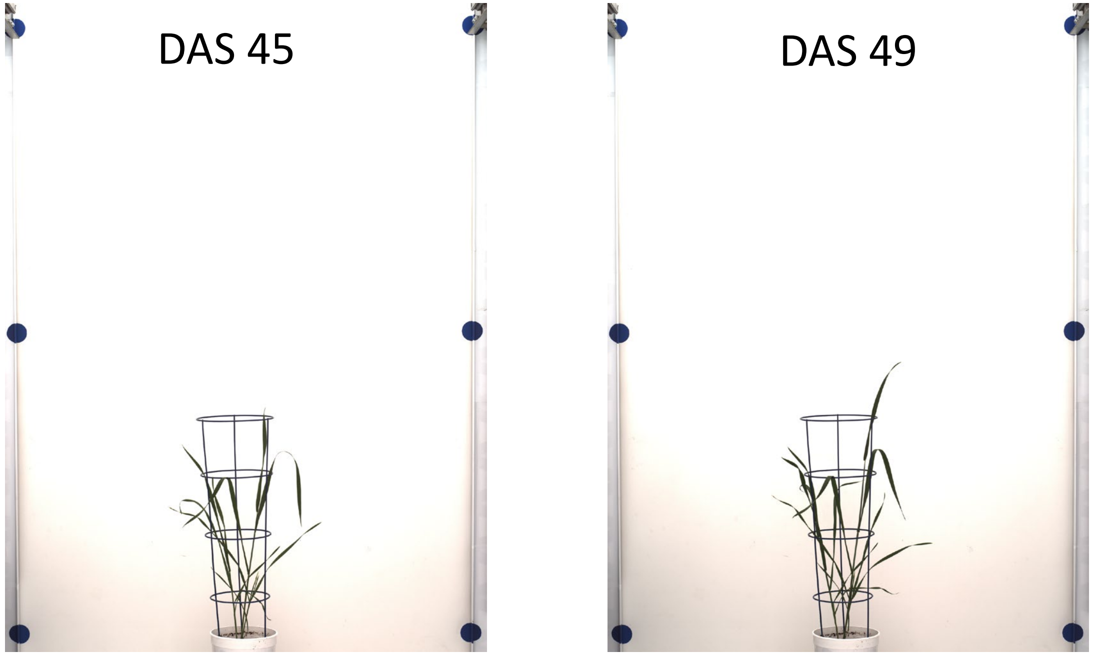

PPPW_046

Control

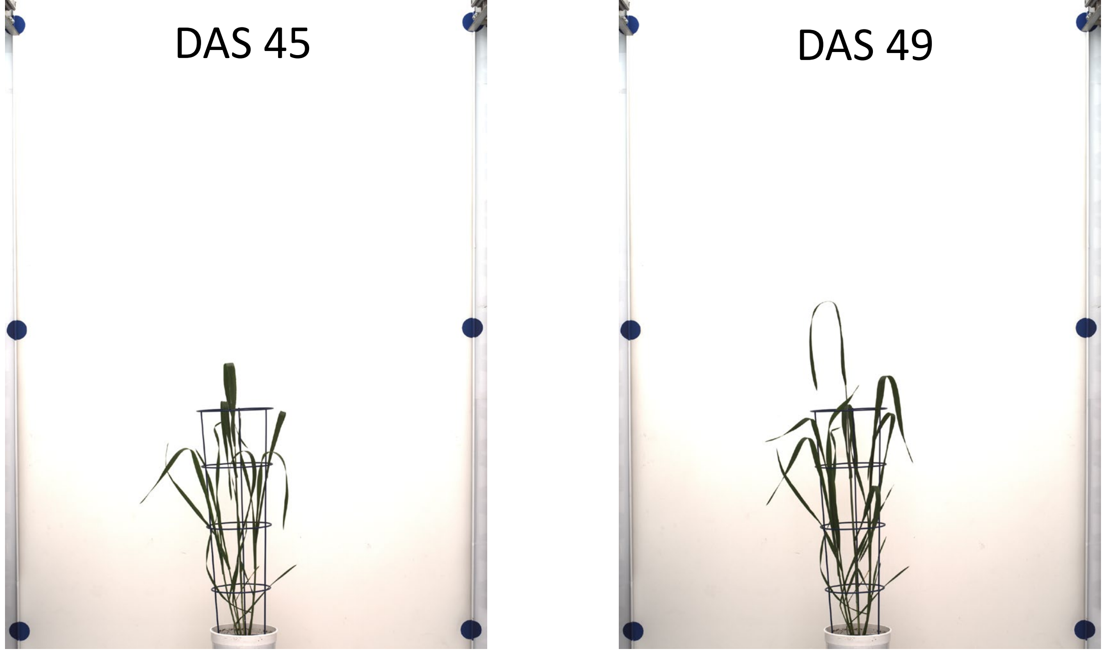

PPPW_046

Drought

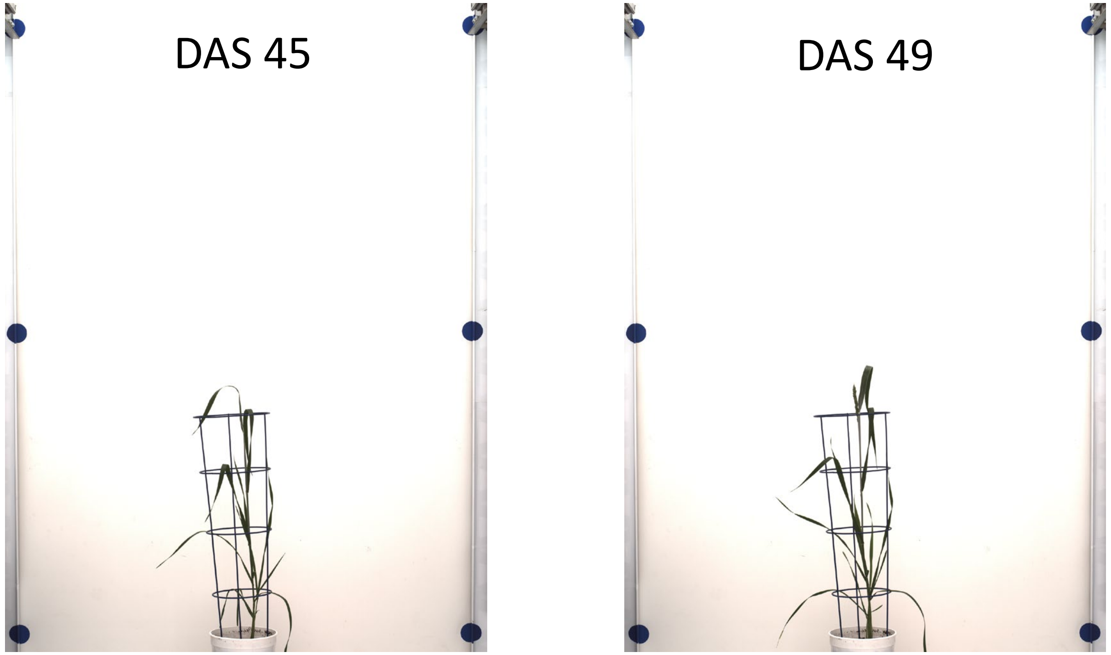

Supplement: Supplementary file 5 — Additional file 5. Examples of images taken at the time point of strongest drought stress (45 days after sowing, DAS) and at the last day of imaging (48 DAS). [file 12870_2025_6914_MOESM5_ESM.pdf]
