# Supplementary figures and images for: Integrating metabolomics and high-throughput phenotyping to elucidate metabolic and phenotypic responses to early-season drought stress in Nordic spring wheat
Source: BMC Plant Biol. 2025 Jul 30;25:987. doi: 10.1186/s12870-025-06914-y (PMC12309201; doi:10.1186/s12870-025-06914-y)

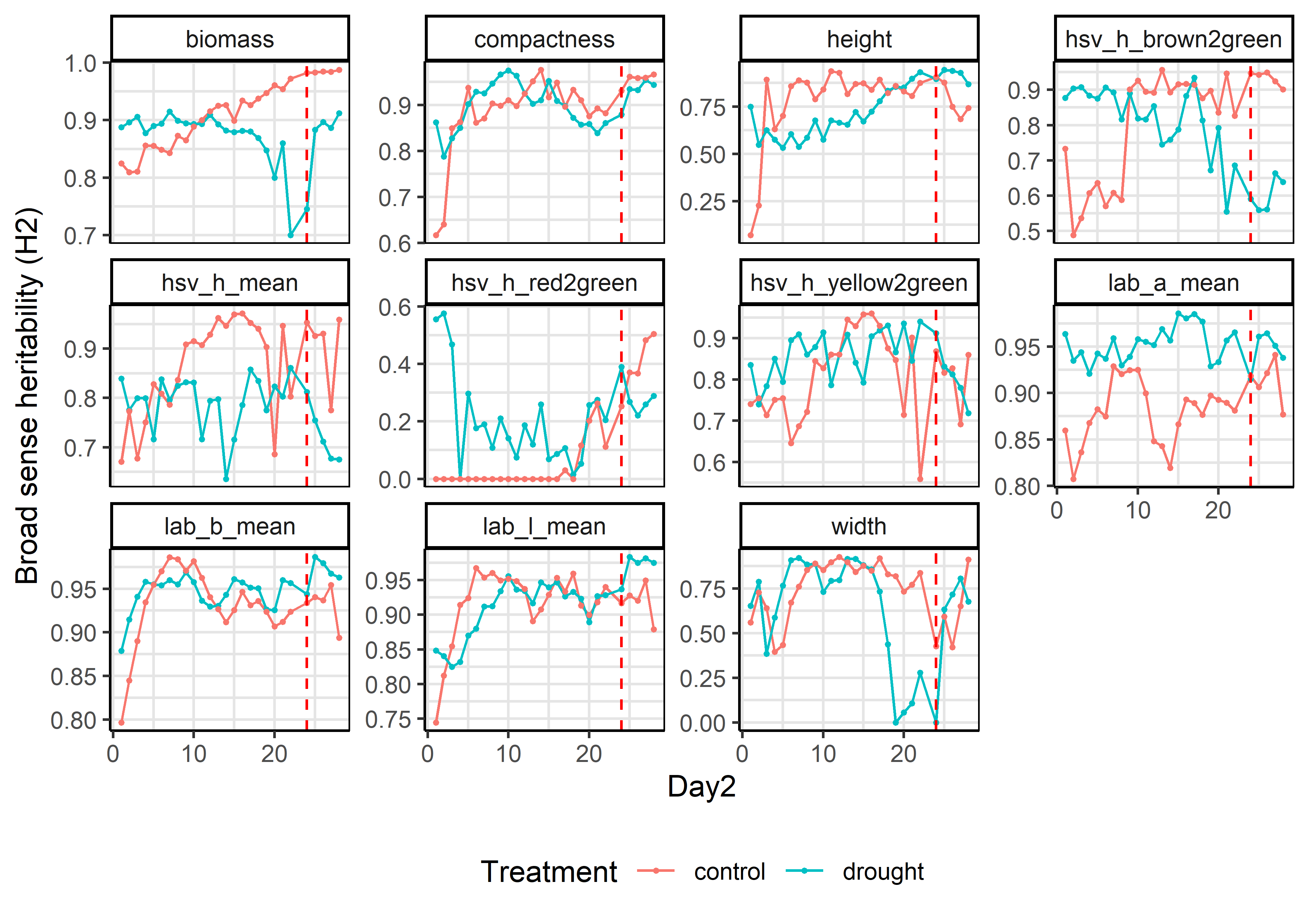

Supplement: Supplementary file 7 — Additional file 7. Broad sense heritability (H2) over time for imaging-based traits across experiments, separately for each treatment. The x-axis shows the days after the onset of drought (DAD). [file 12870_2025_6914_MOESM7_ESM.tiff]

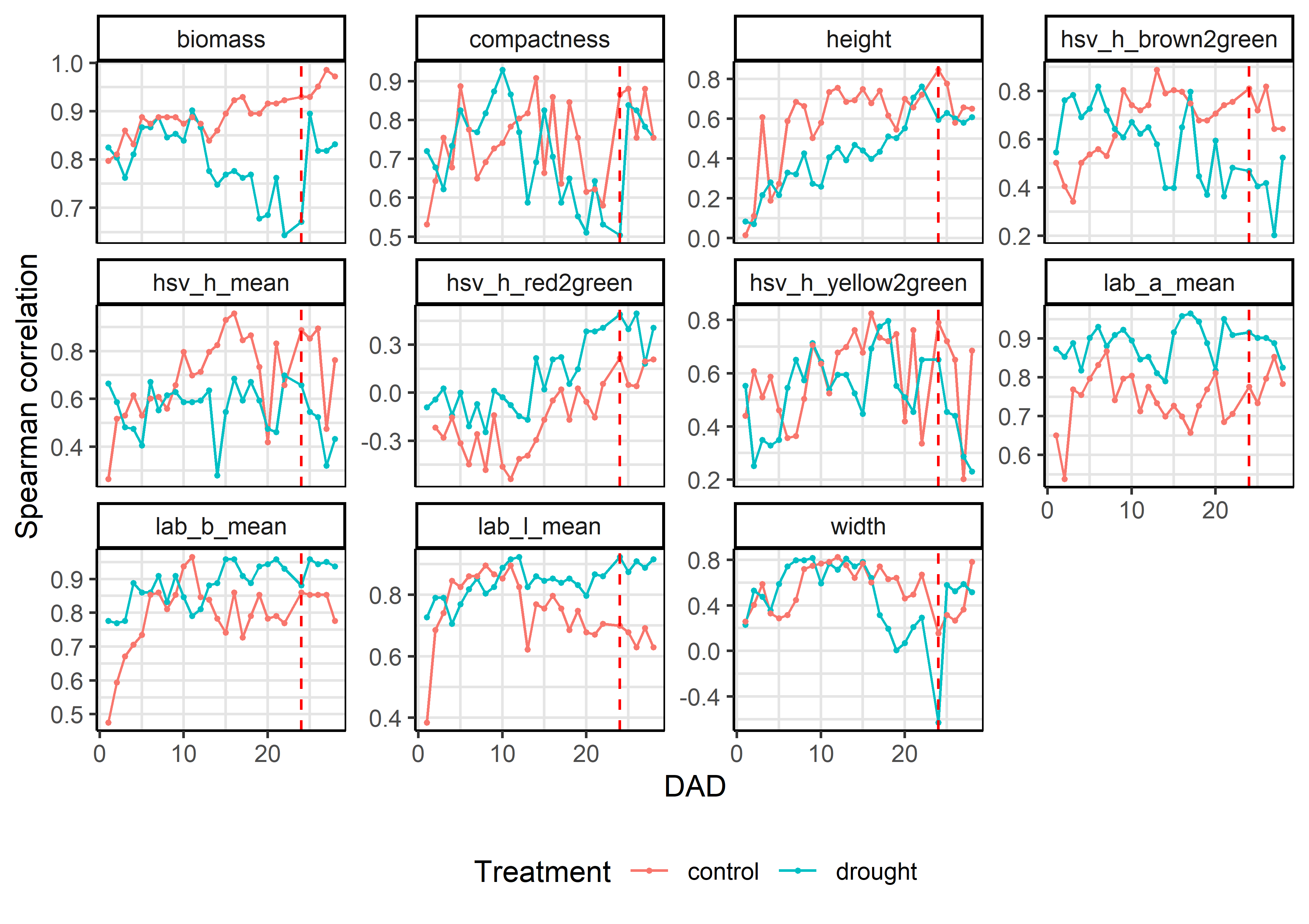

Supplement: Supplementary file 8 — Additional file 8. Spearman’s ρ between both experiments over time for imaging-based traits across experiments, separately for each treatment. The x-axis shows the days after the onset of drought (DAD). [file 12870_2025_6914_MOESM8_ESM.tiff]

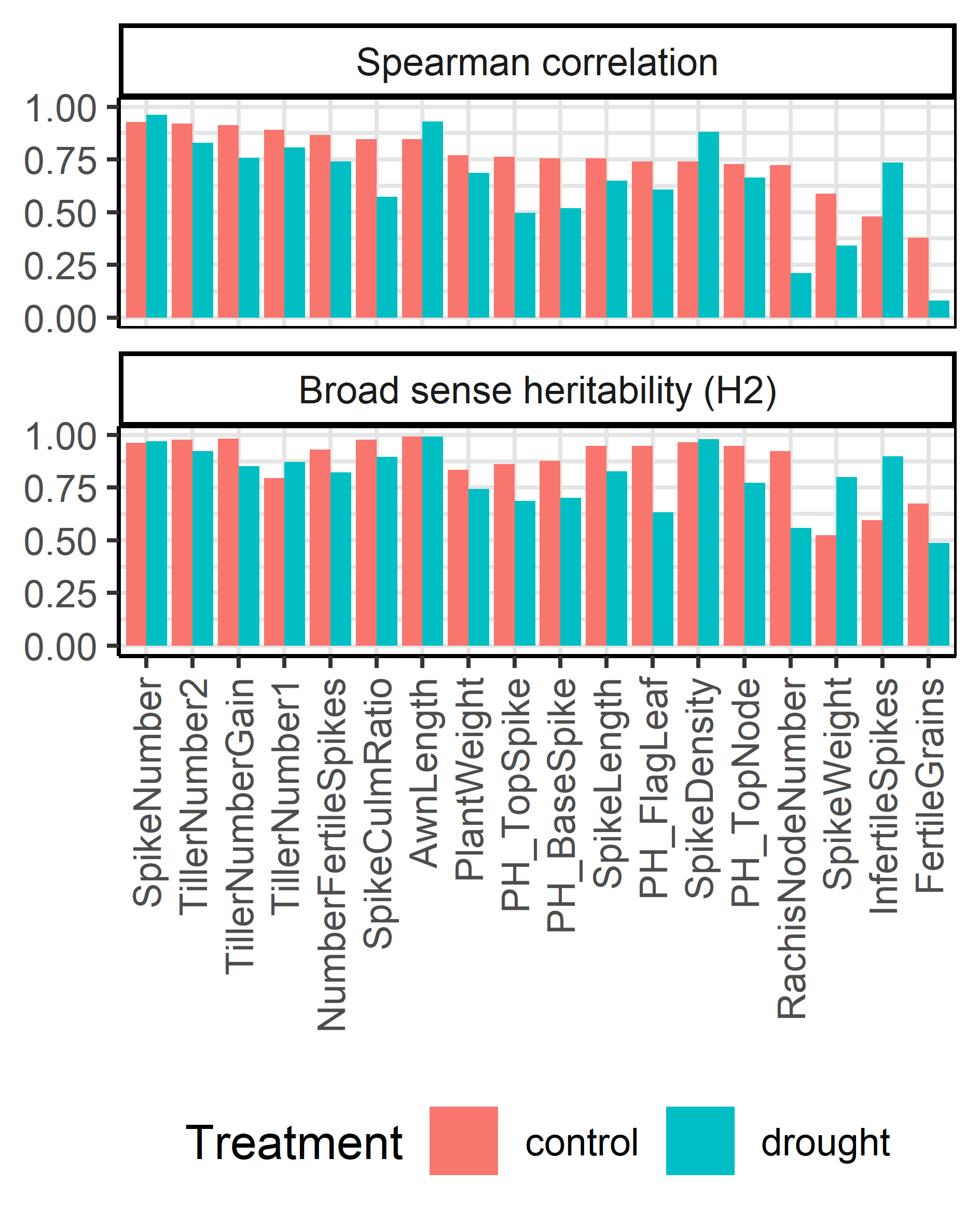

Supplement: Supplementary file 9 — Additional file 9. Spearman’s ρ and broad sense heritability (H2) for traits evaluated after harvest across experiments, separately for each treatment. [file 12870_2025_6914_MOESM9_ESM.tiff]

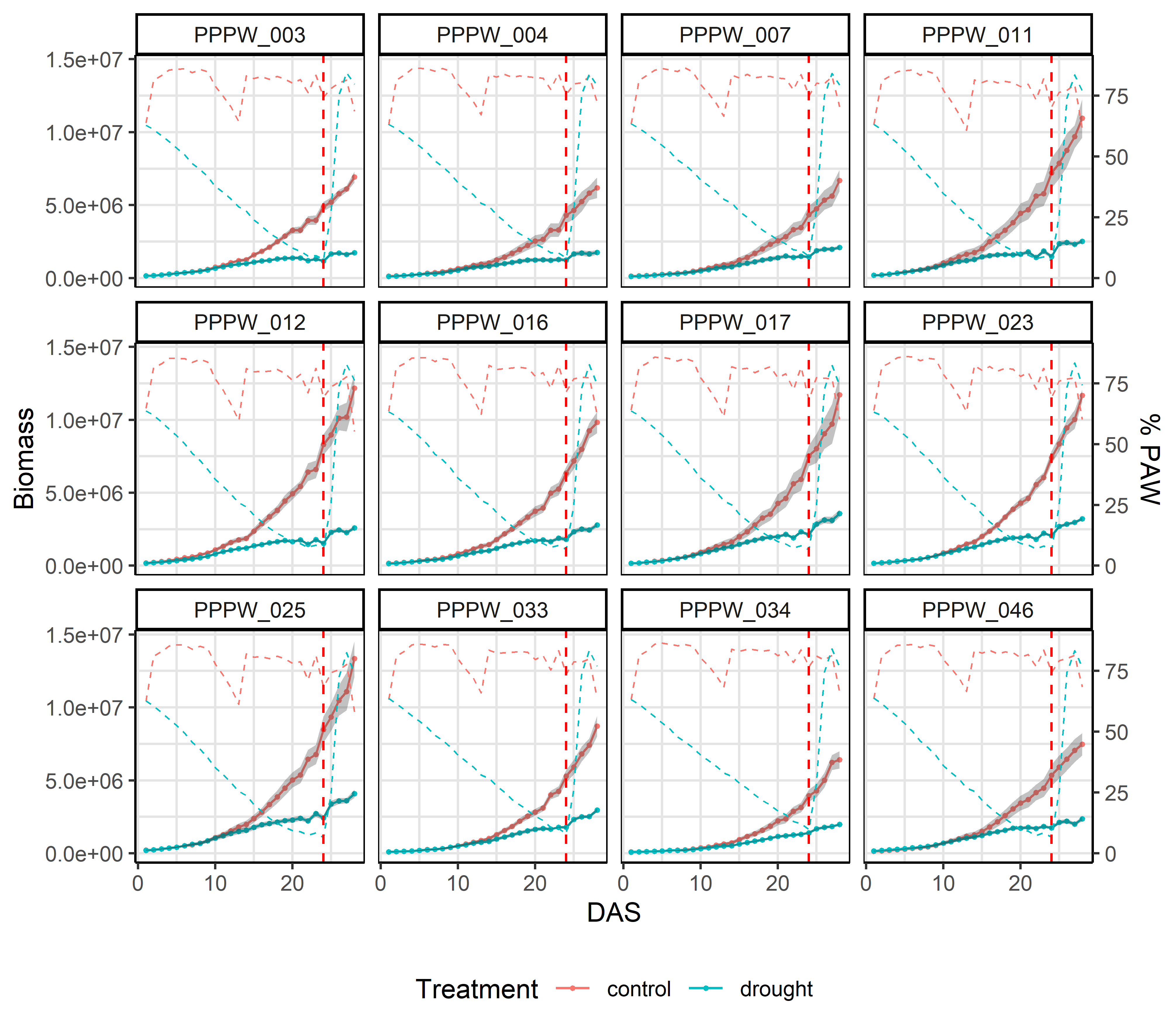

Supplement: Supplementary file 10 — Additional file 10. Biomass accumulation (in voxels) over time for each genotype. Grey areas show 95% confidence intervals, and dashed curves indicate percent PAW over the experiment. Red dashed vertical line marks the end of the drought phase. The x-axis shows the days after the onset of drought (DAD). The y-axis shows biomass in voxels. [file 12870_2025_6914_MOESM10_ESM.tiff]

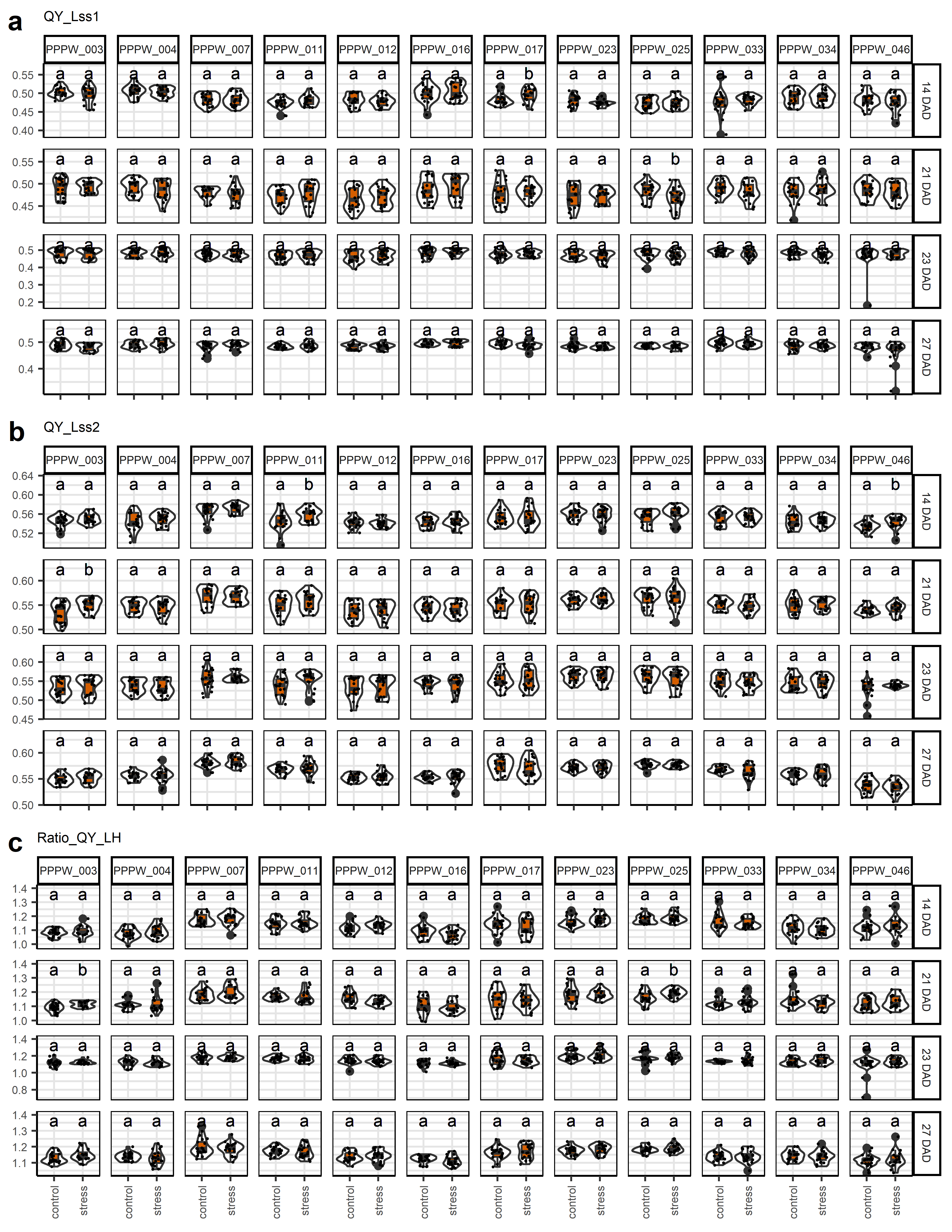

Supplement: Supplementary file 13 — Additional file 13. PSI measurements for each genotype and treatment at four time points (14, 21, 23, and 27. The x-axis shows the days after the onset of drought, DAD): a) Quantum yield at high light intensity (800 μm/m²/s), b) Quantum yield at low light intensity (80 μm/m²/s), c) Ratio of the two quantum yields. [file 12870_2025_6914_MOESM13_ESM.tiff]

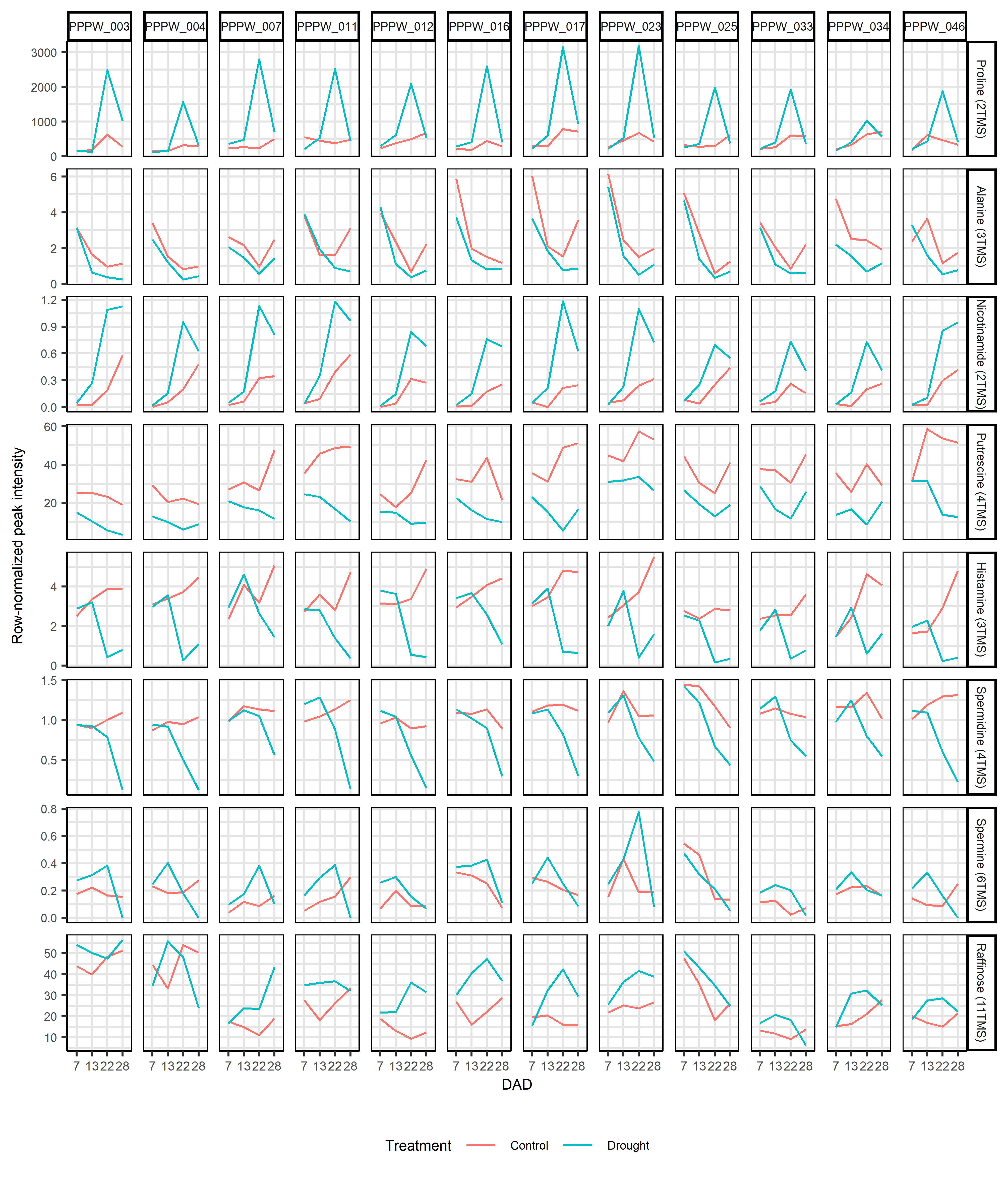

Supplement: Supplementary file 14 — Additional file 14. Row-normalized peak intensities of known drought-responsive metabolites at the four sampling time points (7, 13, 22, and 28). The x-axis shows the days after the onset of drought, DAD) for each genotype and treatment. [file 12870_2025_6914_MOESM14_ESM.tiff]

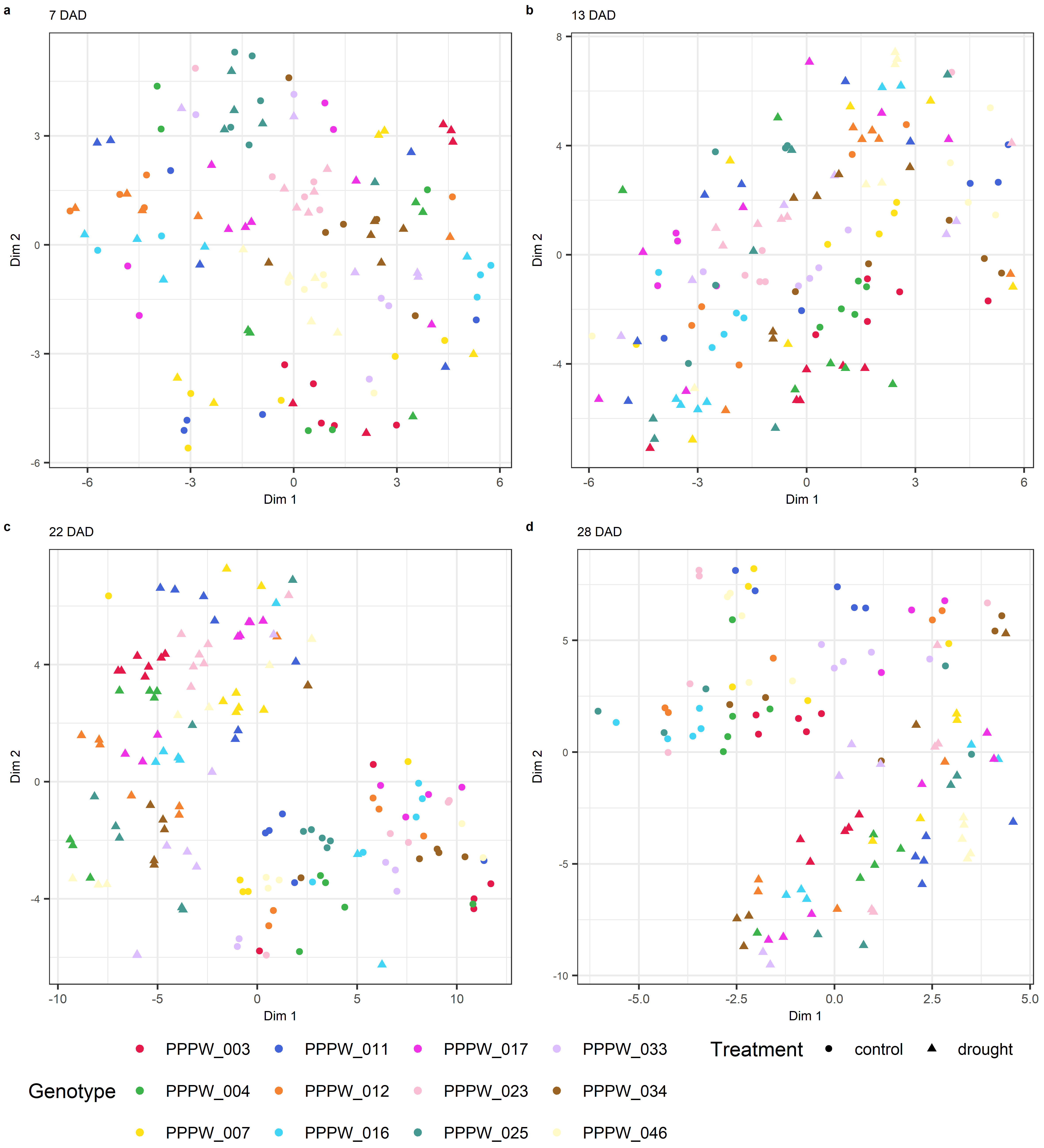

Supplement: Supplementary file 15 — Additional file 15. T-distributed stochastic neighbor embedding (t-SNE) analysis of metabolic profiles by individual plant and treatment. a) 7 days after the onset of drought (DAD), b) 13 DAD, c) 22 DAD and d) 28 DAD. Colours indicate biological replicates per genotype, shapes indicate treatment. [file 12870_2025_6914_MOESM15_ESM.tiff]

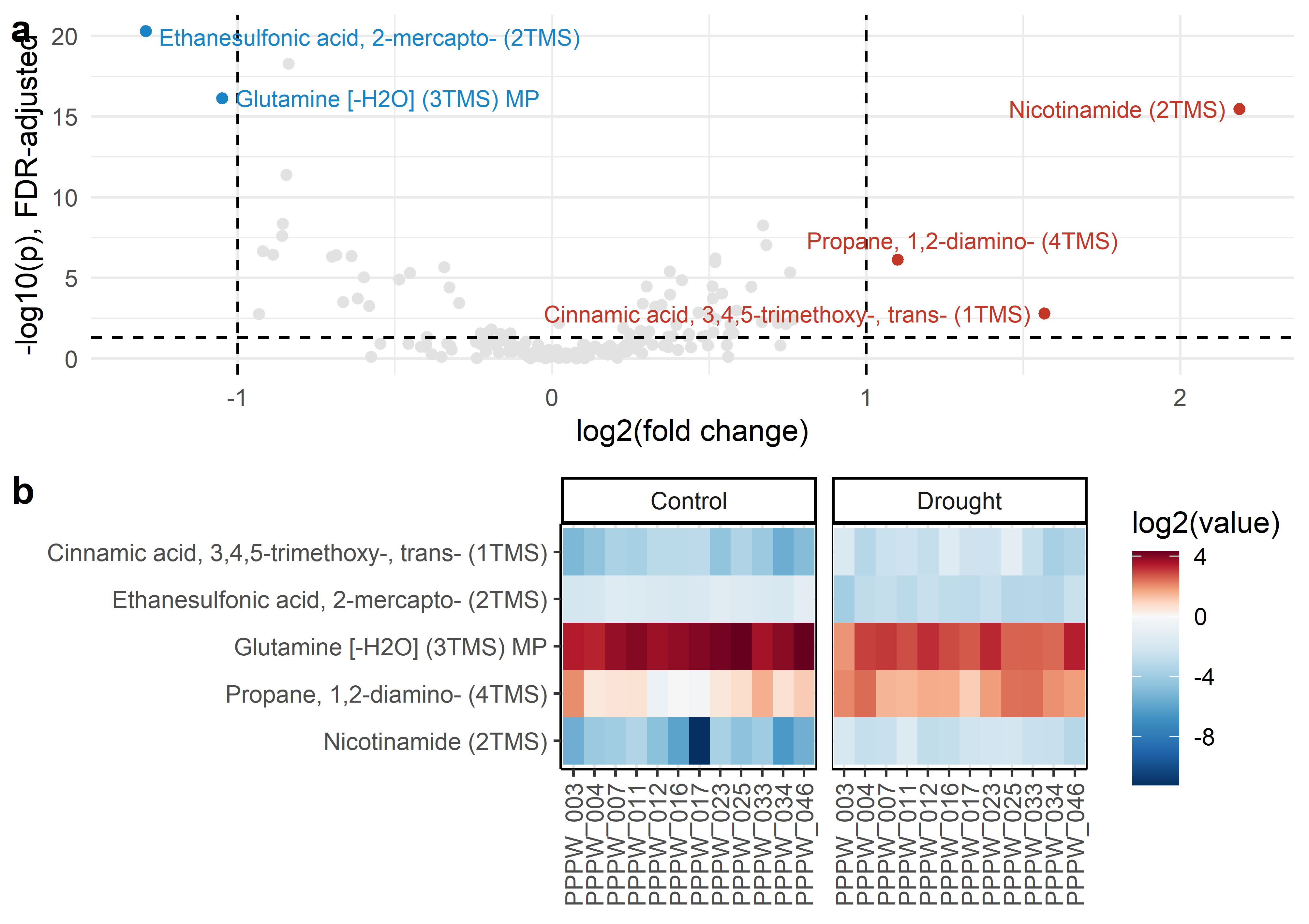

Supplement: Supplementary file 17 — Additional file 17. Metabolic responses to drought at 13 days after the onset of drought (DAD). a) Volcano plot showing differentially accumulated metabolites (DAMs) accumulated (red) reduced (blue) under drought compared to control treatment (T-test, FDR-adjusted p< 0.05, -1 < fold change < 1). b) Log2-transformed, row-normalized intensities of metabolites. [file 12870_2025_6914_MOESM17_ESM.tiff]

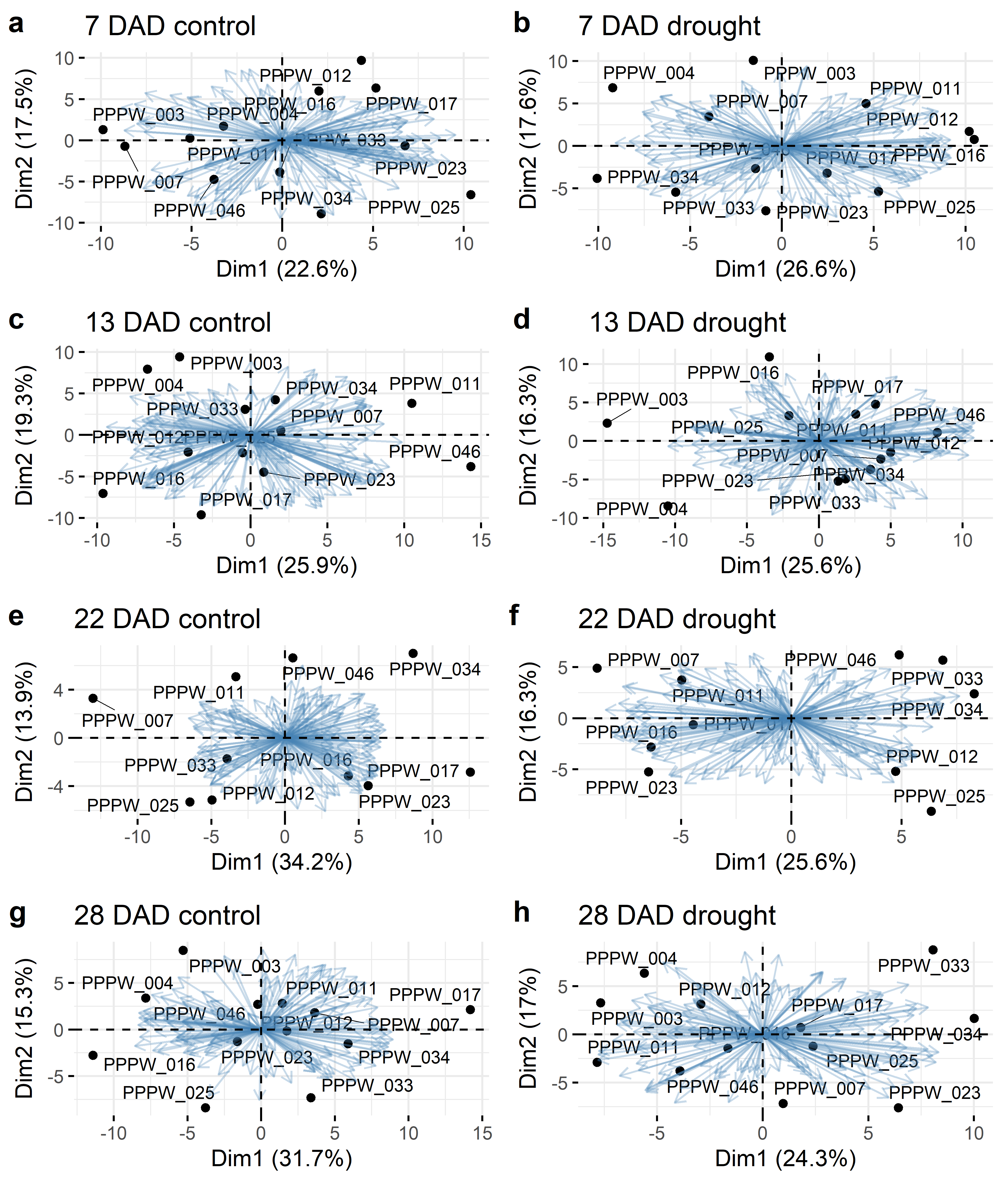

Supplement: Supplementary file 18 — Additional file 18. Biplots placing the genotypes based on their metabolic profile at different time points (7, 13, 22, and 28 days after the onset of drought, DAD) under different conditions. Arrows represent metabolites, with length and direction indicating their impact. [file 12870_2025_6914_MOESM18_ESM.tiff]

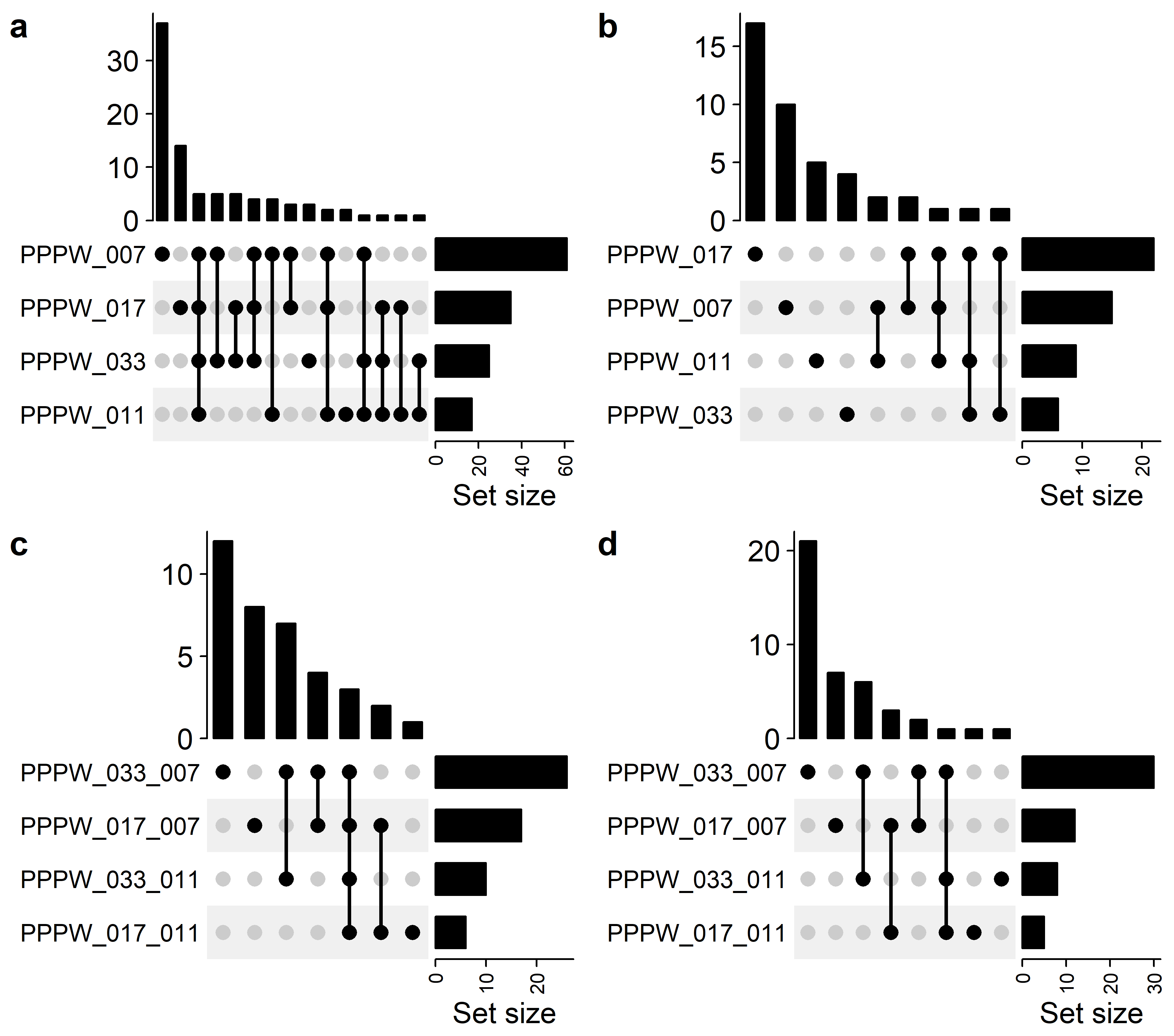

Supplement: Supplementary file 19 — Additional file 19. Differentially accumulated metabolites in tolerant and non-tolerant genotypes. Panels a and b: Metabolites differentially accumulated in tolerant (PPPW_017 and PPPW_033) and non-tolerant (PPPW_007 and PPPW_011) genotypes under drought compared to control conditions at 22 days after the onset of drought (DAD). Upset plots show the distribution of metabolites increased (a) and reduced (b) under drought in tolerant and non-tolerant genotypes. Panels c and d: Metabolites differentially accumulated between tolerant and non-tolerant genotypes under drought at 22 DAD. Upset plots show the number of metabolites increased (c) and reduced (d) under drought in tolerant vs. non-tolerant pairwise comparisons. [file 12870_2025_6914_MOESM19_ESM.tiff]

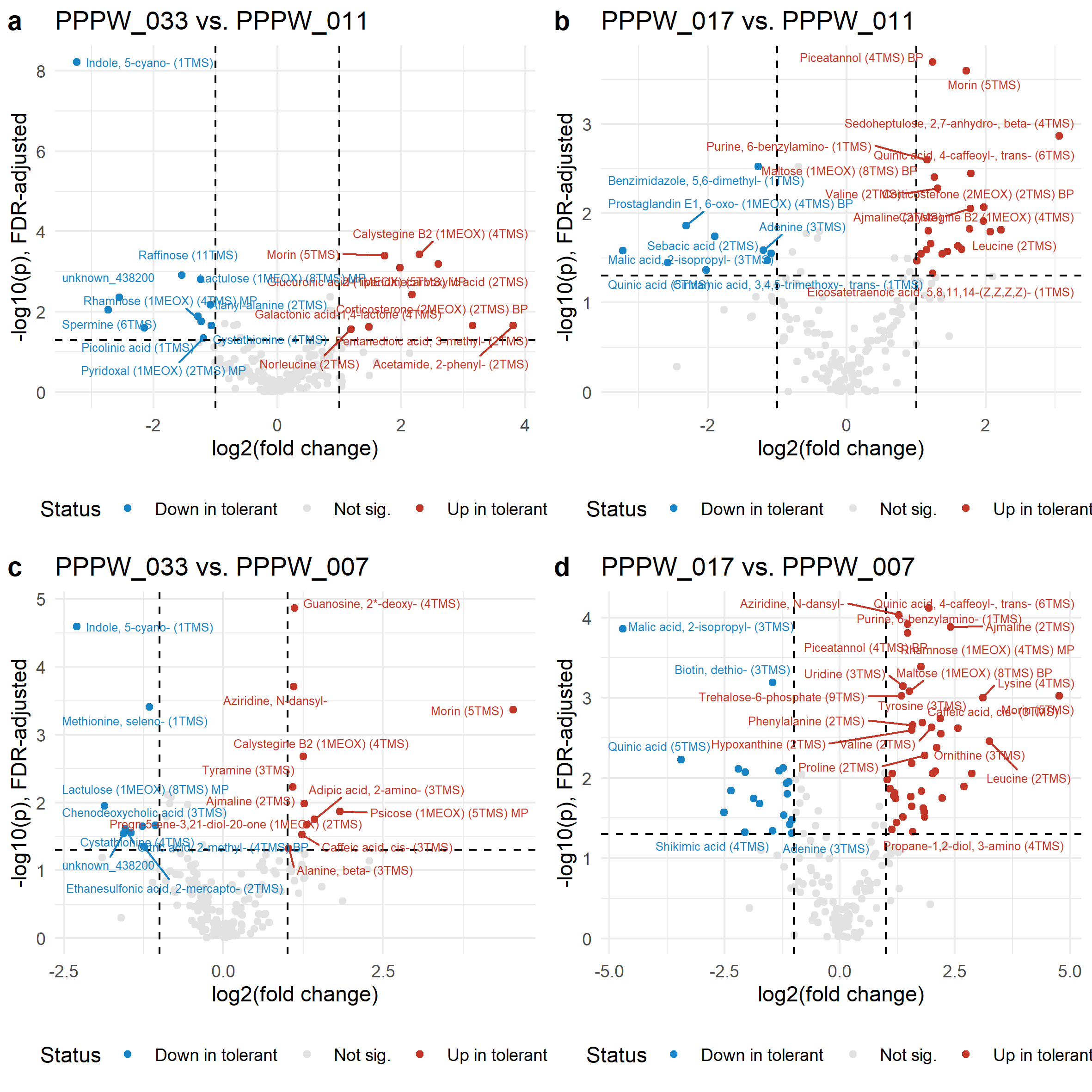

Supplement: Supplementary file 20 — Additional file 20. Differentially accumulated metabolites in pairwise comparisons between tolerant and non-tolerant genotypes. Volcano plot showing differentially accumulated metabolites (DAMs) accumulated (red) reduced (blue) in the tolerant genotype (T-test, FDR-adjusted p < 0.05, -1 < fold change< 1). A) PPPW_003 vs. PPPW_011, b) PPPW_017 vs. PPPW_011, c) PPPW_033 va.PPPW_007, d) PPPW_017 vs. PPPW_007. [file 12870_2025_6914_MOESM20_ESM.tiff]

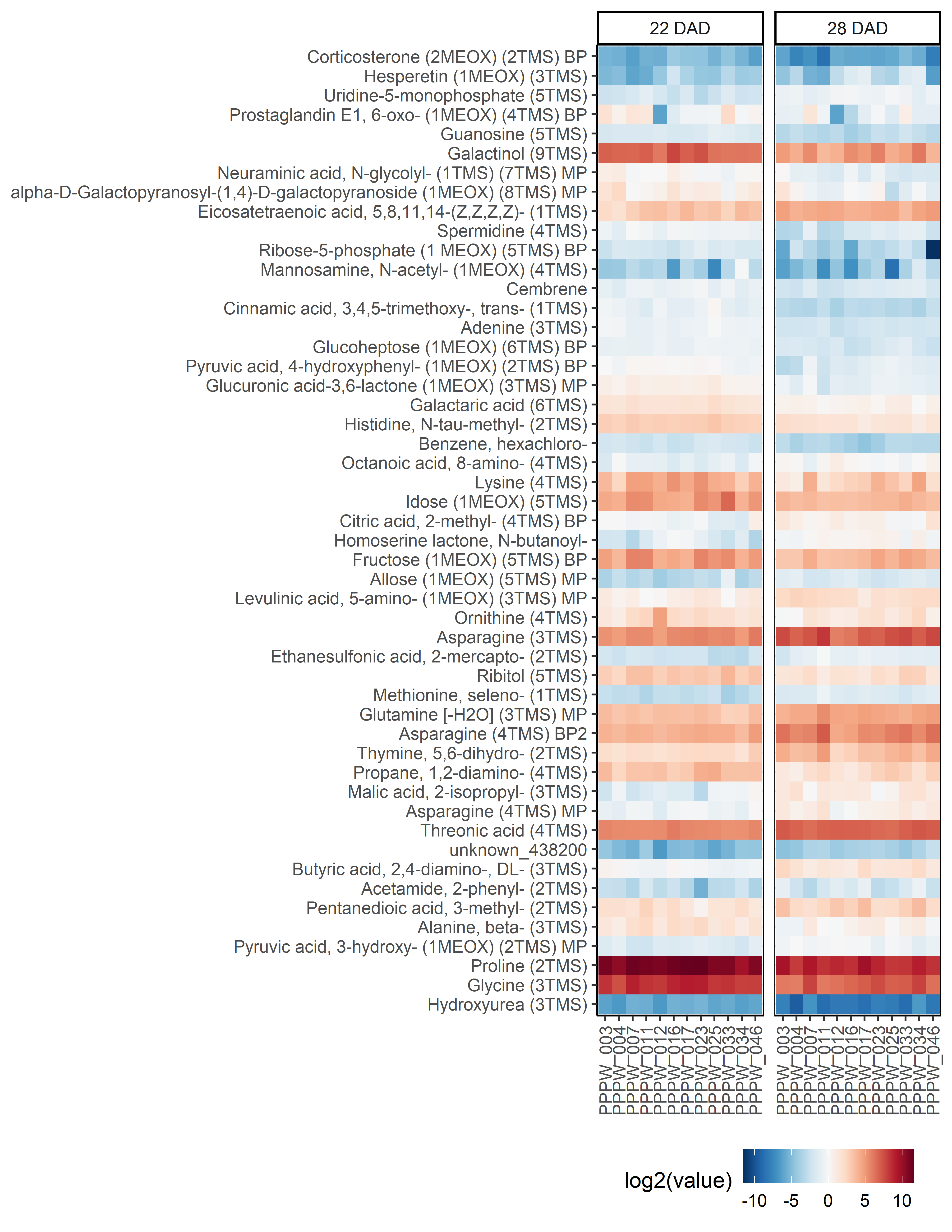

Supplement: Supplementary file 21 — Additional file 21. Log2-transformed, row-normalized intensities of metabolites identified in Fig. 11a (differentially accumulated metabolites between 22 days after the onset of drought (DAD) and 28 DAD, after rewatering). [file 12870_2025_6914_MOESM21_ESM.tiff]
